# Clinical effectiveness of internet-delivered self-help Aacceptance and Commitment Therapy for family carers of people with dementia (iACT4CARERS): a multicentre, parallel, randomised controlled trial

**DOI:** 10.1016/j.lanepe.2026.101766

**Published:** 2026-07-11

**Authors:** Naoko Kishita, Rebecca L. Gould, Mizanur Khondoker, Lance M. McCracken, Megan Riggey, Ana Paula Trucco, David Howe, Abbey Couchman, Emma Flanagan, Ramesh Vishwakarma, Cecile Guillard, Polly-Anna Ashford, David Turner, Andrés Losada-Baltar, Isabel Cabrera-Lafuente, Laura Gallego-Alberto, Erica Richmond, Barbara Czyznikowska, Matthew Hammond, Aditya Nautiyal, Morag Farquhar

**Affiliations:** a School of Health Sciences, University of East Anglia, Norwich, UK; b Division of Psychiatry, University College London, London, UK; c Norwich Medical School, University of East Anglia, Norwich, UK; d Department of Psychology, Uppsala University, Uppsala, Sweden; e Norwich Clinical Trials Unit, University of East Anglia, Norwich, UK; f Departamento de Psicología, Universidad Rey Juan Carlos, Madrid, Spain; g Departamento de Psicología Biológica y de la Salud, Universidad Autónoma de Madrid, Madrid, Spain; h Interface Psychology Team, Norfolk and Suffolk NHS Foundation Trust, Norwich, UK; i Centre for Ethnic Health Research, University of Leicester, Leicester, UK; j The Clarkson Surgery, Wisbech, UK

**Keywords:** Dementia, Caregivers, Anxiety, Self-help, Online therapy, Acceptance and Commitment Therapy

## Abstract

**Background:**

As dementia prevalence rises globally, unpaid family carers provide most care and face high mental health risk. Existing psychological interventions offer limited benefit for anxiety in this population. We evaluated the effectiveness of an internet-delivered, self-help Acceptance and Commitment Therapy intervention with minimal non-expert therapist support (iACT4CARERS) to reduce anxiety in dementia family carers.

**Methods:**

We conducted a multi-site, single-blind, parallel-group, randomised controlled trial across UK national healthcare services. Eligible participants were family carers of a person with dementia and reported anxiety symptoms (Generalised Anxiety Disorder-7 [GAD-7] score ≥5). Participants were allocated (1:1) to iACT4CARERS plus treatment-as-usual or treatment-as-usual alone, using computer-generated randomisation with minimisation by ethnicity and baseline anxiety. The intervention comprised an eight-session self-help programme guided by non-expert therapists. Outcome assessors and the lead statistician were blinded to allocation. The primary outcome was anxiety symptoms, assessed using the GAD-7 at 12-weeks post-randomisation. Analyses followed the intention-to-treat principle using linear regression models. The trial was pre-registered with ISRCTN (45995725).

**Findings:**

Between November 1, 2023, and January 14, 2025, 902 individuals were referred: 567 (63%) were assessed for eligibility and 496 (87%) were randomised (249 to intervention; 247 to control). 397/496 (80%) were female. Primary outcome data were available for 398/496 participants (80%). After adjustment for baseline anxiety, ethnicity, and education, iACT4CARERS plus treatment-as-usual significantly reduced anxiety at 12 weeks compared with treatment-as-usual alone (adjusted mean difference −2.62, 95% CI −3.47 to −1.77; p < 0.0001; d = 0.53), with participants in the intervention group showing clinically meaningful improvement. Three non-intervention-related adverse events were reported; no serious adverse events occurred.

**Interpretation:**

iACT4CARERS is an effective intervention for reducing anxiety symptoms and addressing a major unmet mental health need in this population, with strong potential for scalable implementation.

**Funding:**

National Institute for Health and Care Research Health Technology Assessment.

**Research in context**

**Evidence before this study**

A 2023 systematic review of Acceptance and Commitment Therapy (ACT) for unpaid carers of people with long-term conditions identified ten studies in family carers of people with dementia, including seven single-arm feasibility studies. Three randomised controlled trials (RCTs) evaluated individual ACT delivered in person or via video call by trained therapists compared with active controls or brief psychoeducation, reporting effect sizes (SMD) of 0.35–0.64 for anxiety symptoms, but with small sample sizes (n = 19–66). We updated the search (MEDLINE, PsycInfo, CINAHL; inception to December 20 2025) using the terms “acceptance and commitment therapy”, “dementia”, and “carer∗”/“caregiver∗”, identifying seven additional studies: one single case study, three single-arm feasibility studies, and three small RCTs (n = 33, 64, 92) of one-to-one ACT delivered by trained therapists via telephone or video call versus standard care or brief psychoeducation. To date, no adequately powered RCT has evaluated ACT for family carers of people with dementia, regardless of delivery format, and no RCTs have examined self-help ACT, which does not require highly qualified therapists.

**Added value of this study**

We conducted the first adequately powered, multicentre RCT (n = 496) evaluating the effectiveness of internet-delivered self-help ACT (iACT4CARERS) for reducing anxiety in family carers of people with dementia, guided by non-expert therapists, plus treatment-as-usual versus treatment-as-usual alone. The intervention tested a minimally therapist-supported, fully remote delivery model across England to maximise scalability and accessibility. After adjustment for baseline anxiety score, ethnicity, and education, iACT4CARERS plus treatment-as-usual significantly reduced anxiety (primary outcome) compared with treatment-as-usual alone at 12- and 24-weeks post-randomisation. Similar benefits were observed for all secondary outcomes, including depressive symptoms, psychological flexibility, and experiential avoidance in caregiving. No serious adverse events were reported, supporting the safety of the intervention.

**Implications of all the available evidence**

Given the rising global prevalence of dementia and the substantial economic contributions of unpaid family carers, establishing accessible, cost-effective, and scalable psychological interventions to support carers’ mental health is a public health priority. Evidence from widely studied cognitive behavioural therapy for dementia carers has generally not shown clear benefit for anxiety. We report the first adequately powered RCT demonstrating that internet-delivered self-help ACT, with minimal non-expert therapist support, effectively reduces both anxiety and depressive symptoms. This multidomain effect is particularly important, given the high prevalence of psychiatric comorbidity among dementia carers which—alongside low support and social isolation—may increase risk of suicidal behaviour. These results suggest that self-help ACT, specifically adapted for dementia carers and requiring minimal non-expert therapist input, could be scaled up for broad community implementation.

## Introduction

Over 55 million people worldwide are currently living with dementia, with a new case diagnosed every 3 s. This figure is projected to rise to 139 million by 2050. The majority of dementia care is provided by family members, representing an estimated global economic contribution of USD 651.4 billion annually.^1^ Systematic reviews indicate that approximately 32% of such dementia family carers experience symptoms of anxiety and depression, with some evidence suggesting anxiety may be even more prevalent.^2^^,^^3^ Despite this high burden, many carers receive little or no psychological support and frequently report feelings of isolation, compounded by barriers such as resource constraints within health and social services.^4^ A remote, home-based, self-help psychological intervention present practical and scalable access to support for this underserved population.

A systematic review of 131 randomised controlled trials (RCTs) of nonpharmacological interventions for dementia family carers, of which 73% used non-active controls, found that psychotherapeutic interventions yield the largest reductions in depressive symptoms.^5^ Most studied interventions have involved manualised cognitive behaviour therapy (CBT), typically delivered in person. Previous meta-analyses targeting dementia family carers demonstrated small effects of face-to-face or telephone CBT on depressive symptoms (Cohen's d = 0.34) and no significant effect on anxiety,^6^ while internet- or DVD-delivered self-help CBT showed even smaller effects (d = 0.27), with anxiety outcomes not assessed.^7^ A more recent review of broader online interventions for dementia family carers identified 17 RCTs, of which three reported anxiety outcomes.^8^ Two evaluated low-intensity multicomponent psychoeducation, and one an intensive programme including online discussion groups, weekly check-ins, and two-way messaging with specialists. Analysed sample sizes were 299, 175, and 25, with a pooled reported effect size (SMD) of 0.33 for anxiety symptoms. These findings highlight the need to identify alternative psychological interventions for this population, particularly with respect to anxiety symptoms.

While conventional CBT primarily focuses on reducing anxious thoughts and feelings, Acceptance and Commitment Therapy (ACT) offers an alternative perspective by recognising that pathologizing anxiety and struggling to control it can, unwittingly, make matters worse. Grounded in functional contextualism, ACT aims to enhance psychological flexibility through three key skills: stepping back from restrictive thoughts and allowing painful emotions (OPEN); focusing on the present (AWARE); and clarifying and acting on what matters most while building effective patterns of values-based action (ENGAGED).^9^ These processes reduce the influence of anxious thoughts on emotional and behavioural responses, lessen unhelpful struggle with anxiety, and free energy for other priorities, thereby increasing engagement in meaningful, emotionally rich activities and ultimately enhancing wellbeing.

A recent systematic review of ACT interventions for unpaid carers of people with long-term conditions identified ten studies targeting dementia carers.^10^ Seven were feasibility or pilot single-arm pre-post studies showing that ACT—delivered via telephone, video conferencing, group therapy, or guided self-help—was feasible and acceptable. The remaining three were small RCTs, with analysed sample sizes of 19–66 participants, reporting effect sizes (SMD) of 0.35–0.64 for anxiety symptoms. These RCTs provided preliminary evidence that in-person or video-conference ACT can reduce anxiety and depressive symptoms, although uncertainties remain due to the small sample sizes. To date, no adequately powered trial has evaluated the clinical effectiveness of internet-delivered self-help ACT for dementia carers. Evidence from systematic reviews of broader populations indicates that internet-delivered ACT, both guided and unguided, can effectively reduce anxiety and depressive symptoms, suggesting that it may also benefit dementia family carers.^11^^,^^12^

This RCT builds on our previous feasibility and acceptability work on internet-delivered self-help ACT for carers of people living with dementia (iACT4CARERS).^13^, ^14^, ^15^, ^16^ The feasibility study met all pre-specified progression criteria, recruiting more than 30 participants across three sites in six months, with over 70% completing at least seven out of eight online sessions, and showed preliminary improvements in anxiety and depressive symptoms, as well as in psychological flexibility,^13^ the hypothesised mechanism of change in ACT.^9^ Therefore, this RCT aimed to evaluate the clinical effectiveness of iACT4CARERS plus treatment-as-usual versus treatment-as-usual alone for reducing anxiety in family carers of people living with dementia experiencing anxiety symptoms.

## Methods

### Study design

The iACT4CARERS trial was a multi-site, single-blind, parallel-group, two-arm RCT of iACT4CARERS plus treatment-as-usual compared to treatment-as-usual alone. The intervention was delivered at 20 secondary care services (mental health or planned/elective care) within the National Health Service (NHS) across England, selected to ensure broad geographic representation.

The trial was pre-registered with the ISRCTN Registry (45995725) on 12 April 2023. The trial protocol has been published and is available open access.^17^ Trial oversight was provided by a Trial Management Group, a Trial Steering Committee, and an independent Data Monitoring and Ethics Committee, which included public advisory members.

### Participants

Eligible participants were adults (≥18 years), including in-laws and unmarried partners, providing unpaid care to a family member with a clinician-verified dementia diagnosis. Inclusion criteria were: a Generalised Anxiety Disorder 7-item scale (GAD-7)^18^ score ≥5 (which corresponds to at least mild anxiety symptoms), a self-reported desire for support with anxiety, and access to a device with internet access. Exclusion criteria included lacking capacity to provide fully informed consent, current engagement in formal psychological therapy at randomisation (e.g., CBT), severe medical or mental health conditions preventing participation, and active suicidal intent. Participants were not required to refrain from receiving psychotherapy during the trial. Individuals on a waiting list at randomisation were also eligible, irrespective of when therapy commenced.

Participants were recruited from 20 NHS sites delivering the intervention, plus six NHS primary care and four NHS secondary care sites involved in recruitment only. Community-based strategies included newspaper advertisements, radio interviews and promotions, the national Join Dementia Research platform, and social media posts via partner charities (e.g., Dementia UK), with targeted outreach to underserved populations. Both clinician and self-referrals were accepted.

### Randomisation and masking

Participants were randomly assigned (1:1) to iACT4CARERS plus treatment-as-usual or treatment-as-usual alone, using minimisation by self-identified ethnicity (Asian, Black, White, Mixed, Other) and baseline GAD-7 score (mild [5–9], moderate [10–14], severe [15+]). Allocation was computer-generated via a centralised system managed by the Norwich Clinical Trials Unit (NCTU), ensuring allocation concealment prior to randomisation.

Allocation was implemented by an unmasked trial manager at NCTU. Intervention participants received automated access to the iACT4CARERS platform via email sent by the centralised system. Participants recruited through trial sites were assigned to a site therapist, while self-referred participants were randomly allocated to trial sites with available therapists. Therapists were automatically notified of allocations via the centralised system. Outcome assessors and the trial statistician were masked to group assignment until primary analyses were completed. Participants and therapists were not masked to allocation.

### Procedures

Following clinician or self-referral, the central study team contacted potential participants and provided the Participant Information Sheet by email or post. Interested individuals attended a remote screening session via video or phone call. Written informed consent was obtained online after which participants completed the screening assessment, including eligibility statements, demographics (e.g., age, self-reported gender and ethnicity), and the GAD-7, through the NCTU-managed central database. Eligible participants were prompted to complete baseline outcome measures online. Those who opted to use paper forms received all documents by post and returned them in a prepaid envelope; data were then manually entered, and eligibility confirmed. Randomisation occurred within one week of screening. Follow-up assessments at 12- and 24-weeks post-randomisation were completed online via automated email prompts. Non-responders were contacted by blinded outcome assessors via phone, with participants instructed not to disclose allocation. Those preferring postal follow-up received questionnaires and prepaid envelopes from the trial manager and receipt confirmed by phone one week later.

iACT4CARERS comprised eight self-guided online sessions in which participants learned and practised core ACT skills through video and audio exercises. Sessions were released sequentially, with each becoming available five days after completion of the previous one. Each session took approximately 30–50 min to complete, depending on the number of self-directed activities. At the end of each session, participants were encouraged to reflect on what they found helpful and identify a small, values-based action to undertake. After each session, therapists provided individually tailored written feedback to support the development of ACT skills and to encourage continued practice. Participants were informed that access to the online programme would end after 12 weeks. During this period, they were also offered the option to book up to two 30-min one-to-one telephone or video sessions with a therapist. These sessions aimed to: (a) explore emotional needs and experiences; (b) address challenges in using a digital intervention; and (c) clarify expectations regarding weekly reflections and therapist feedback to help tailor support. Session-by-session details are provided in Appendix 21 and in the open-access study protocol.^17^ Participants in both arms continued to receive treatment-as-usual during the trial. The modified Client Service Receipt Inventory (CSRI)^19^ was used to assess treatment-as-usual during the trial. Participants completed a resource-use diary to aid self-report.

Of the 65 therapists involved, 19 held clinical qualifications (e.g., nurse, occupational therapist, physiotherapist), but none had formal qualifications in psychological therapies, such as clinical psychology. The remaining therapists did not hold clinical qualifications (e.g., dementia support workers, assistant psychologists, occupational therapy associates). Therapists primarily supported participants in completing self-directed learning by facilitating reflection and continued practice of skills between sessions, rather than delivering ACT; therefore, clinical qualifications were not required. The minimum criterion was prior experience working directly with healthcare service users. All therapists completed a two-day video-call training course covering ACT principles, providing online feedback, and using the manual for one-to-one sessions, which included a topic guide and example scripts. Drop-in group supervision was offered fortnightly via video call throughout the trial. Therapist-written feedback was digitally recorded, and a single session per participant, randomly selected, was rated by independent ACT experts using the ACT Fidelity Measure (ACT-FM),^20^ evaluating the extent to which therapist responses were consistent or inconsistent with core ACT principles. Therapist response time (i.e., the time between a participant's comment and the therapist's reply) was also recorded for each session.

### Outcomes

The primary outcome was anxiety symptoms, assessed using the GAD-7,^18^ with the primary endpoint at 12 weeks post-randomisation. Secondary outcomes included GAD-7 scores at 24 weeks, depressive symptoms assessed by the Patient Health Questionnaire-9 (PHQ-9),^21^ psychological flexibility assessed by the Comprehensive Assessment of Acceptance and Commitment Therapy processes (CompACT),^22^ and experiential avoidance assessed by the Experiential Avoidance in Caregiving Questionnaire (EACQ),^23^ measured at both 12 and 24 weeks post-randomisation. The CompACT comprises three subscales reflecting key aspects of psychological flexibility: openness to experience, behavioural awareness, and valued action. The EACQ includes three subscales assessing distinct facets of experiential avoidance in caregiving: active avoidant behaviours, intolerance of negative thoughts and emotions towards the relative, and apprehension regarding negative internal experiences related to caregiving. Total and subscale scores on the CompACT and EACQ were included as secondary outcomes.

The EQ-5D-5L,^24^ a measure of health status, the ICEpop CAPability measure for Older people (ICECAP-O),^25^ which assesses a broader aspect of wellbeing than health status, and the modified CSRI,^19^ which captures use of health and social care resources, were also collected at three timepoints to inform the economic (cost-utility) analysis, which is reported elsewhere. This paper therefore presents only the baseline characteristics of these variables, except for the CSRI, for which 12-week data are included to characterise treatment-as-usual (Appendices 1–5). Treatment satisfaction in the intervention group was assessed at 12 weeks using the Satisfaction With Therapy and Therapist Scale-Revised (STTS-R).^26^ Potential sources of bias were evaluated at baseline using two 5-point numerical rating items measuring treatment expectations and two 5-point items assessing intervention preference, adapted from measures used in previous trials (see Appendix 22 for full questionnaire items).^27^ Adverse events were monitored and recorded throughout the trial period.

### Statistical analysis

Systematic reviews of RCTs of self-help ACT and mindfulness-based interventions (primarily targeting individuals with mental or physical health conditions) have reported a pooled effect size of d = 0.35 for anxiety symptoms.^28^^,^^29^ To detect an effect size of d = 0.35 between the two trial arms with 90% power and a two-tailed significance level of 5%, 173 participants were required per arm. In our feasibility study of iACT4CARERS, 27% of participants were lost to follow-up. Assuming a 30% attrition rate, the final recruitment target for the trial was 496 carers.

Statistical analyses were pre-specified in the Statistical Analysis Plan (SAP), which was reviewed and approved by the Trial Steering Committee and the Data Monitoring and Ethics Committee. The plan was uploaded to the trial registry prior to completion of data collection (https://doi.org/10.1186/ISRCTN45995725). All analyses were conducted using Stata version 19.

All randomised participants were included in the analyses in the group to which they were allocated, in accordance with the intention-to-treat (ITT) principle. Descriptive statistics summarised demographic characteristics, baseline measures, and treatment adherence, using appropriate summary measures selected according to the type and distribution of each variable. To evaluate the clinical effectiveness on the primary and secondary outcomes, we initially fitted linear mixed-effects models with random intercepts for site and therapist, with therapist nested within site. As neither random effect significantly improved model fit (Appendix 6), indicating no meaningful clustering, these terms were excluded in accordance with the pre-specified SAP, and linear regression models were used for all analyses. Regression models were estimated using maximum likelihood (ML) method with the glm command in Stata, assuming a Gaussian family and identity link function. ML estimation was chosen over ordinary least squares to enhance robustness against potential bias from missing outcome data.

All models were adjusted, as specified in the SAP, for baseline GAD-7 score and ethnicity (minimisation factor). Ethnicity was collapsed into a binary variable (1 = White, 0 = non-White) owing to small counts within some categories. Participant education level was included as an additional covariate due to an observed imbalance between the trial arms, with the primary school category merged with the secondary O-level (GCSE) category due to small number of participants in the primary school group. All secondary outcomes were analysed using analogous regression models, with baseline values of the respective outcome measures included as an additional covariate.

The distribution of residuals from regression models were assessed using histograms and normal probability plots along with skewness and kurtosis statistics (Appendix 7). These diagnostics indicated approximate normality, supporting the validity of the model assumptions.

Several sensitivity analyses were also conducted for the primary outcome, GAD-7. Potential impacts of missing outcome data were examined using multiple imputation by chained equations. With 20–26% of data missing at 12 and 24 weeks, 30 imputed datasets were generated in line with current methodological guidance.^30^^,^^31^ The multiple imputation model included baseline variables associated with missingness (including ethnicity, age, education level, employment status, referral route, length of caregiving, and extra-trial treatment), as well as treatment allocation, baseline GAD-7, and outcome measures at all time points, to support the Missing At Random assumption. A Complier Average Causal Effect (CACE) analysis within the ITT population was performed to evaluate whether inadequate treatment compliance influenced the estimated treatment effect for the primary outcome. The CACE estimation was implemented using the generalised structural equation modelling (gsem) procedure in Stata.^32^ The compliance in the intervention arm was fixed at the observed rate (78.7% completing six or more online sessions). Compliance in the control arm, which is a counterfactual, was estimated probabilistically using a latent class model within the gsem framework which included baseline GAD-7 score, ethnicity, treatment expectation, treatment preference, and the indicator of receiving extra-trial treatments as predictors. The indicator of extra-trial treatment was defined as receiving medication for mental health, psychotherapy, or both within the past three months. Finally, additional sensitivity analyses assessed the effects of referral route (self-referred or clinician-referred) and of treatment expectation and preference (measured before randomisation) as covariates in the primary ITT analysis.

In addition, further exploratory analyses examined potential factors influencing treatment response within the intervention arm, including therapist fidelity (assessed via the ACT-FM and therapist response time) and number of completed online sessions, as specified in the SAP. Correlations between these variables and GAD-7 scores at 12 weeks were assessed. Associations between changes in psychological flexibility (measured by the CompACT) from baseline to 12 and 24 weeks and GAD-7 scores at the corresponding time points were also examined.

Three additional exploratory analyses, not pre-specified in the SAP, were conducted with Trial Steering Committee approval. In addition to exploratory correlations, causal mediation analysis examined whether changes in psychological flexibility (CompACT) from baseline to 12 weeks mediated the intervention effect on anxiety (GAD-7) at 24 weeks. A regression-based parametric causal mediation model was fitted using Stata's mediate command. Linear models were specified for both the outcome and mediator, adjusting for baseline anxiety, ethnicity, and education. The model included a treatment–mediator interaction, allowing the mediator effect on the outcome to differ by treatment group (Appendix 18). Indirect, direct, and total effects of the intervention on GAD-7 at 24 weeks were estimated, along with the proportion of the total effect mediated via change in CompACT. Standard errors and 95% confidence intervals were derived using non-parametric bootstrap with 1000 replications and the percentile method. Clinical meaningfulness was assessed by the proportion of participants achieving a Minimal Clinically Important Difference (MCID) of 20% improvement in GAD-7 scores^33^ from baseline to 12 and 24 weeks. The MCID represents the smallest change perceived as important by patients, consistent with a patient-centred definition of feeling better. GAD-7 severity ranges were also used to demonstrate shifts in severity categories (e.g., from severe to moderate) at each time point for both groups.

### Ethics statement

Ethical approval was granted by the NHS London-Queen Square Research Ethics Committee on 30 March 2023 (REC reference: 23/LO/0188). All participants provided written informed consent, and all data were de-identified before analysis.

### Role of the funding source

The funder of the study had no role in study design, data collection, data analysis, data interpretation, or writing of the report.

## Results

**Fig. 1:**
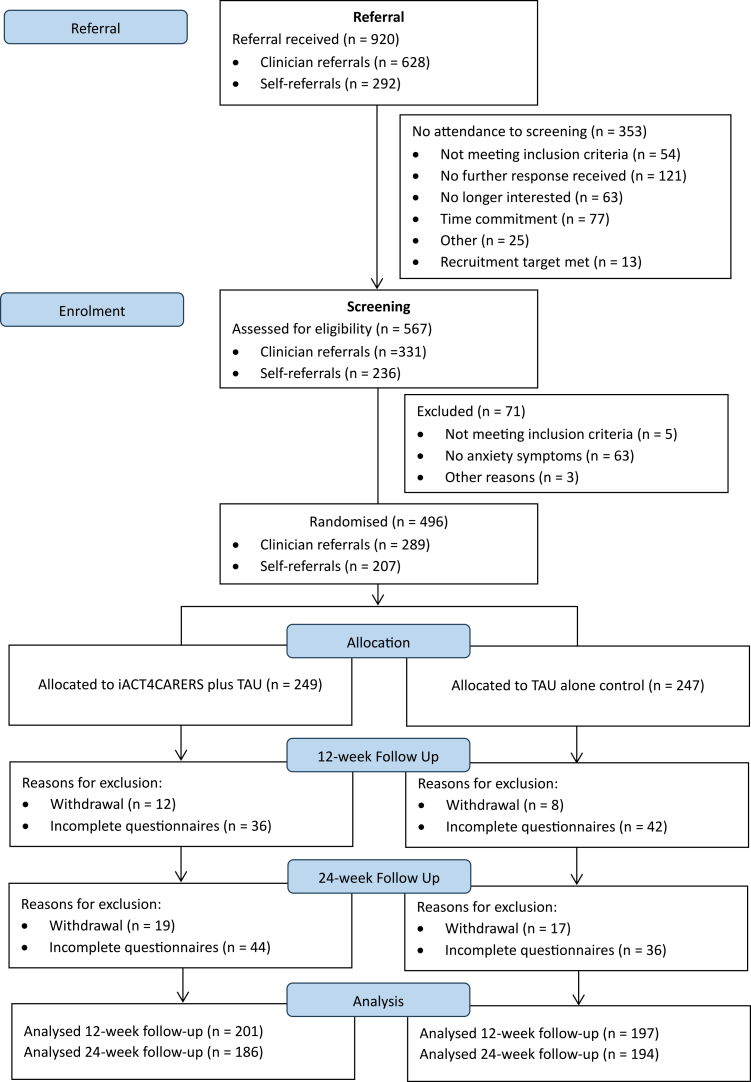
**Trial profile.** iACT4CARERS = Internet-delivered Self-help Acceptance and Commitment Therapy for Family Carer of People Living with Dementia. TAU = Treatment as Usual.

Participants were recruited between Nov 1, 2023, and Jan 14, 2025, six months shorter than planned due to early achievement of the recruitment target. Overall, 902 individuals were referred, of whom 567 (63%) were assessed for eligibility. Of these, 496 (87%) met the inclusion criteria and were randomised: 249 to iACT4CARERS plus treatment-as-usual and 247 to treatment-as-usual alone (Fig. 1). Fewer than half of participants across groups received dementia advice or education as treatment-as-usual; 20% accessed carer support groups, and 2% received psychotherapy outside iACT4CARERS (Appendices 4 and 5). The number of participants who withdrew from the trial or did not complete questionnaires at 12 and 24 weeks was similar across groups. Reasons for withdrawal (Appendix 8) were similar across groups, with no clear pattern suggesting differential withdrawal related to outcomes. Withdrawals were few in both arms and reflected changes in the condition of the person with dementia, participants’ health, or practical circumstances. Data for the primary outcome measure (GAD-7 at 12 weeks) were available for 398 (80%) of 496 participants.

The key baseline characteristics of participants and their care recipients are presented in Tables 1 and 2. Baseline characteristics were well balanced between the two groups, except for education level. Education level, along with the minimisation variables (baseline GAD-7 score and ethnicity), was included as a potential confounder in the analyses of primary and secondary outcomes. Most participants were female (80%) and identified as the sole primary carer responsible for the care recipient (79%). A minority were from non-White ethnic groups (8%) and only 1% reported primary school as their highest level of education. With respect to potential sources of bias, baseline expectations of symptom improvement and preference for receiving iACT4CARERS over treatment-as-usual were similar across groups.

**Table 1:** Baseline characteristics of family carers.

	iACT4CARERS (n = 249)	TAU only (n = 247)
Age, years		
Mean (SD)	61.93 (11.58)	62.95 (10.68)
Range	22–83	29–91
Gender		
Female	202 (81.1%)	195 (79.0%)
Male	47 (18.9%)	52 (21.0%)
Ethnicity		
Asian	10 (4.0%)	9 (3.6%)
Black	5 (2.0%)	6 (2.4%)
Mixed	4 (1.6%)	3 (1.2%)
White	229 (92.0%)	227 (91.9%)
Other ethnic groups	1 (0.4%)	2 (0.8%)
Relationship		
Wife	88 (35.3%)	84 (34.0%)
Husband	24 (9.6%)	33 (13.4%)
Partner	7 (2.8%)	9 (3.6%)
Daughter	97 (39.0%)	100 (40.5%)
Daughter-in-law	4 (1.6%)	4 (1.6%)
Son	20 (8.0%)	16 (6.5%)
Son-in-law	2 (0.8%)	0 (0%)
Other	7 (2.8%)	1 (0.4%)
Education		
Primary school	2 (0.8%)	3 (1.2%)
Secondary school O′ level/GCSE	33 (13.3%)	56 (22.7%)
Secondary school A′ level	31 (12.5%)	24 (9.7%)
Vocational diploma	59 (23.7%)	47 (19.0%)
Bachelor's degree	76 (30.5%)	74 (30.0%)
Postgraduate degree	48 (19.3%)	43 (17.4%)
Current work status		
Part-time job	50 (20.1%)	45 (18.2%)
Full-time job	46 (18.5%)	43 (17.4%)
Not currently working	21 (8.4%)	25 (10.1%)
Retired	132 (53.0%)	134 (54.3%)
Type of care		
Sole primary carer	196 (78.7%)	195 (79.0%)
Shared (co-primary) carer	53 (21.3%)	52 (21.1%)
Length of caregiving in months		
Median (IQR)	38 (33)	41 (37)
Range	2–289	4–473
Expectations about treatment total score (possible range 0–8)		
Median (IQR)	4 (2)	4 (2)
Range	0–8	0–8
Treatment preference total score (possible range 0–8)		
Median (IQR)	6 (3)	7 (3)
Range	2–8	2–8
Extra trial treatment at baseline^a^		
Received	53 (21.3%)	51 (20.7%)
Not received	196 (78.7%)	196 (79.4%)
Extra trial treatment at 12 weeks^b^		
Received	44 (22.0%)	40 (20.7%)
Not received	156 (78.0%)	153 (79.2%)
STTS-R Satisfaction with therapy (possible range 6–30)^c^		
Median (IQR)	25.5 (6)	–
Range	11–30	–
STTS-R Satisfaction with the therapist (possible range 6–30)^c^		
Median (IQR)	23.0 (6)	–
Range	9–30	–

iACT4CARERS = Internet-delivered Self-help Acceptance and Commitment Therapy for Family Carer of People Living with Dementia. IQR = Interquartile range. TAU = Treatment as Usual. STTS-R = Satisfaction with Therapy and Therapist Scale-Revised.

Scores range from 6 to 30, with higher scores indicating greater satisfaction; a total of 18 generally reflects overall satisfaction, despite variation across items (i.e., most or all items not falling into the ‘strongly disagree’ or ‘disagree’ categories).

a Extra trial treatment is defined as receiving medication, psychotherapy, or both for mental health problems within the past three months.

b Data on extra trial treatment at 12 weeks were available for 200 and 193 participants in the treatment and control arms, respectively; these numbers were used as denominators.

c Only participants allocated to the treatment arm completed the STTS-R (n = 200).

**Table 2:** Baseline characteristics of care recipients.

	iACT4CARERS (n = 249)	TAU only (n = 247)
Age, years		
Mean (SD)	78.95 (8.55)	78.72 (9.12)
Range	53–102	53–98
Gender		
Female	121 (48.6%)	131 (53.0%)
Male	128 (51.4%)	116 (47.0%)
Dementia diagnosis		
Alzheimer's disease	129 (51.8%)	106 (42.9%)
Vascular dementia	35 (14.1%)	28 (11.3%)
Dementia with Lewy bodies	13 (5.2%)	9 (3.6%)
Frontotemporal dementia	5 (2.0%)	15 (6.1%)
Mixed dementia	51 (20.5%)	67 (27.1%)
Other type of dementia	5 (2.0%)	13 (5.3%)
Dementia type unknown	11 (4.4%)	9 (3.6%)
Living condition		
In their own home alone	66 (26.5%)	61 (24.7%)
Living in the same household as the carer (study participant)	134 (53.8%)	137 (55.5%)
Living with other family member(s)	31 (12.5%)	25 (10.1%)
In a care home	17 (6.8%)	22 (8.9%)
In a hospital	1 (0.4%)	2 (0.8%)

iACT4CARERS = Internet-delivered Self-help Acceptance and Commitment Therapy for Family Carer of People Living with Dementia. TAU = Treatment as Usual.

Of the 249 participants allocated to iACT4CARERS, 196 (79%) completed six or more sessions within the intended timeframe and were classified as completers per the pre-defined threshold (session-by-session completion rates are shown in Appendix 9). A total of 174 (70%) attended at least one optional telephone or video session with a therapist (see Appendix 9 for further details). Mean STTS-R scores for satisfaction with therapy and therapist were high (Table 1); among 200 respondents, 98% rated the therapy and 86% rated the therapist as satisfactory (≥18/30).

Primary and secondary outcomes at all timepoints are presented in Table 3, with data completeness shown in Appendix 10. Missing data occurred mainly at the participant level, consistent with withdrawal or loss to follow-up. Logistic regression of missingness indicator identified several observed covariates (including ethnicity, age, education level, employment status, referral route, length of care, and extra trial treatment at baseline) associated with missingness, supporting the plausibility of the Missing At Random assumption (Appendix 11). The primary outcome analysis indicated that iACT4CARERS plus treatment-as-usual was significantly more effective than treatment-as-usual alone in reducing anxiety symptoms at 12 weeks post-randomisation (adjusted mean difference on the GAD-7 −2.62, 95% CI −3.47 to −1.77; d = 0.53; p < 0.0001). This between-group difference remained evident at 24 weeks (adjusted mean difference −2.12, 95% CI −3.01 to −1.20; d = 0.42; p < 0.0001). Similar patterns of significant between-group differences were observed for all remaining clinical outcomes, including depressive symptoms (PHQ-9), psychological flexibility (CompACT total and subscale scores), and experiential avoidance (EACQ total and subscale scores), at both 12- and 24-week follow-up (Table 3).

**Table 3:** Primary and secondary outcome measures at 12 weeks and 24 weeks post-randomisation for iACT4CARERS plus treatment as usual and treatment as usual alone groups.

	iACT4CARERS		TAU only		Mean difference (95% CI)	Cohen's *d* (95% CI)	Adjusted mean difference (95% CI)	p values
	n	Mean (SD)	n	Mean (SD)				
GAD-7 (possible range 0–21)								
Baseline	249	11.55 (4.43)	247	11.56 (4.45)	−0.02 (−0.77, 0.80)	–	–	–
12 weeks	201	6.71 (4.61)	197	9.30 (5.07)	−2.59 (−3.55, −1.64)	0.53 (0.34, 0.74)	−2.62 (−3.47, −1.77)	<0.0001
24 weeks	186	6.88 (4.94)	194	9.00 (5.18)	−2.12 (1.10, 3.14)	0.42 (0.21, 0.62)	−2.11 (−3.01, −1.20)	<0.0001
PHQ-9 (possible range 0–27)								
Baseline	249	9.87 (5.40)	247	10.41 (5.26)	−0.54 (−1.48, 0.40)	–	–	–
12 weeks	201	6.42 (5.10)	197	8.78 (5.68)	−2.36 (−3.42, −1.30)	0.44 (0.24, 0.64)	−2.12 (−3.03, −1.21)	<0.0001
24 weeks	186	6.53 (5.57)	194	8.78 (5.74)	−2.26 (−3.40, 1.11)	0.40 (0.20, 0.60)	−1.94 (−2.91, −0.97)	<0.0001
CompACT (total possible range 0–138)								
Baseline	249	70.57 (18.55)	246	67.96 (17.46)	2.61 (−0.57, 5.80)	–	–	–
12 weeks	201	82.76 (21.00)	194	72.20 (20.49)	10.56 (6.45, 14.66)	0.51 (0.31, 0.71)	8.56 (5.16, 11.97)	<0.0001
24 weeks	185	82.87 (22.77)	193	71.90 (20.98)	10.97 (6.55, 15.40)	0.50 (0.30, 0.71)	9.71 (6.04, 13.38)	<0.0001
CompACT OE (possible range 0–60)								
Baseline	249	25.61 (8.91)	247	25.72 (8.54)	0.89 (−0.65, 2.43)	–	–	–
12 weeks	201	33.63 (9.69)	194	28.45 (9.73)	5.17 (3.25, 7.09)	0.53 (0.33, 0.73)	4.47 (2.88, 6.06)	<0.0001
24 weeks	185	33.79 (10.81)	193	28.67 (10.55)	5.12 (2.96, 7.28)	0.48 (0.27, 0.68)	4.64 (2.82, 6.47)	<0.0001
CompACT BA (possible range 0–30)								
Baseline	249	12.90 (7.12)	246	12.57 (6.15)	0.33 (−0.84, 1.51)	–	–	–
12 weeks	201	16.34 (7.32)	194	13.89 (6.65)	2.45 (1.07, 3.84)	0.35 (0.15, 0.55)	2.25 (1.15, 3.36)	<0.0001
24 weeks	185	16.34 (7.47)	193	13.64 (7.11)	2.70 (1.22, 4.17)	0.37 (0.17, 0.57)	2.76 (1.55, 3.97)	<0.0001
CompACT VA (possible range 0–48)								
Baseline	249	31.06 (8.23)	247	29.74 (8.70)	1.32 (−0.17, 2.81)	–	–	–
12 weeks	201	32.79 (9.06)	194	29.86 (9.01)	2.93 (1.14, 4.72)	0.32 (0.13, 0.52)	2.06 (0.48, 3.65)	0.0107
24 weeks	185	32.74 (9.08)	193	29.58 (8.94)	3.15 (1.33, 4.98)	0.35 (0.15, 0.55)	2.49 (0.91, 4.08)	0.0021
EACQ total (possible range 15–75)								
Baseline	249	41.39 (7.46)	247	42.91 (7.71)	−1.52 (−2.86, −0.18)	–	–	–
12 weeks	201	36.89 (8.29)	192	42.70 (7.95)	−5.81 (−7.42, −4.20)	0.72 (0.51, 0.92)	−4.11 (−5.43, −2.78)	<0.0001
24 weeks	184	37.01 (8.86)	190	41.52 (8.58)	−4.52 (−6.29, −2.74)	0.52 (0.31, 0.72)	−2.79 (−4.26, −1.32)	0.0002
EACQ AAB (possible range 6–30)								
Baseline	249	17.17 (4.08)	247	18.00 (4.41)	−0.83 (−1.58, −0.82)	–	–	–
12 weeks	201	15.32 (4.51)	192	17.70 (4.39)	−2.37 (−3.26, −1.49)	0.53 (0.33, 0.73)	−1.72 (−2.51, −0.93)	<0.0001
24 weeks	184	15.54 (4.56)	190	17.19 (4.51)	−1.65 (−2.57, −0.72)	0.36 (0.16, 0.57)	−0.98 (−1.81, −0.15)	0.0206
EACQ INTE (possible range 4–20)								
Baseline	249	12.79 (3.48)	247	13.25 (3.48)	−0.46 (−1.08, 0.15)	–	–	–
12 weeks	201	11.37 (3.37)	194	13.82 (3.41)	−2.45 (−3.12, −1.78)	0.72 (0.52, 0.93)	−1.82 (−2.38, −1.26)	<0.0001
24 weeks	184	11.18 (3.73)	191	13.05 (3.29)	−1.87 (−2.58, −1.15)	0.53 (0.33, 0.74)	−1.33 (−1.96, −0.70)	<0.0001
EACQ ACNIE (possible range 5–25)								
Baseline	249	11.43 (3.06)	247	11.65 (3.21)	−0.22 (−0.78, 0.33)	–	–	–
12 weeks	201	10.20 (3.01)	194	11.21 (3.34)	−1.01 (−1.64, −0.39)	0.32 (0.12, 0.52)	−0.75 (−1.31, −0.19)	0.0092
24 weeks	184	10.28 (2.93)	190	11.30 (3.08)	−1.02 (−1.63, −0.41)	0.34 (0.14, 0.54)	−0.81 (−1.35, −0.28)	0.0030

CompACT = Comprehensive Assessment of Acceptance and Commitment Therapy processes. CompACT OE = CompACT Openness to Experience. CompACT BA = CompACT Behavioural Awareness. CompACT VA = CompACT Valued Action. EACQ = Experiential Avoidance in Caregiving Questionnaire. EACQ AAB = EACQ Active Avoidant Behaviours. EACQ ACNIE = EACQ Apprehension Concerning Negative Internal Experiences Related to Caregiving. EACQ INTE = EACQ Intolerance of Negative Thoughts and Emotions Towards the Relative. GAD-7 = Generalized Anxiety Disorder-7. iACT4CARERS = Internet-delivered Self-help Acceptance and Commitment Therapy for Family Carer of People Living with Dementia. PHQ-9 = Patient Health Questionnaire-9. TAU = Treatment as Usual.

Effect sizes for most primary and secondary outcomes were in the medium range at 12 weeks (approximately 0.50). Smaller effects were observed for the awareness and valued action subscales of the CompACT and the caregiving-related apprehension subscale of the EACQ (0.32–0.35), whereas the EACQ total score and the intolerance of negative thoughts and emotions towards the relative subscale showed the largest effects (0.72). Effect sizes were broadly similar at 24 weeks, although outcomes with large effects at 12 weeks declined to the medium range.

No serious adverse events were reported during the trial. Among 496 participants randomised, three non-intervention-related adverse events occurred: two cases of suicidal ideation without intent and one hospitalisation for a medical condition (Appendix 13).

The primary outcome (GAD-7 at 12 weeks) remained robust in sensitivity analyses using multiple imputation for missing data and CASE estimation accounting for treatment compliance, referral route, and treatment expectation and preference, supporting the effectiveness of iACT4CARERS in reducing anxiety symptoms compared with treatment-as-usual (Appendices 14 and 17).

Exploratory analyses showed no statistically significant association between therapist fidelity variables, including ACT-FM consistency (n = 200; r = −0.06, p = 0.4010), inconsistency (n = 200; r = 0.05, p = 0.4552), and therapist response time (n = 201; r = 0.03, p = 0.6354), and GAD-7 scores at 12 weeks in the treatment group. Median ACT-FM consistency and inconsistency scores for randomly selected online feedback scripts were 1.75 (IQR 2.00) and 0 (IQR 1.00), respectively (maximum 36 per subscale). The mean therapist response time was 4.22 days (SD 1.64), where a score of 1 indicated that feedback was provided on the same day participants submitted their online comments. Of the 1643 pieces of therapist feedback submitted, 93% were provided within the intended 7-day period. In contrast, the number of online sessions completed was significantly associated with GAD-7 scores at 12 weeks (n = 201; r = −0.21, p = 0.0031), with more sessions linked to lower anxiety symptoms. Changes in CompACT total and subscale scores from baseline to 12 and 24 weeks were associated with GAD-7 scores at corresponding timepoints in both groups (Table 4). Increases in psychological flexibility and its components (openness, awareness, and valued action) over time were associated with lower anxiety.

**Table 4:** Associations between changes in psychological flexibility and its components and anxiety symptoms in both groups.

	Change scores from baseline to 12 weeks						Correlations with GAD-7 at 12 weeks					
	iACT4CARERS		TAU only		Difference with covariates		iACT4CARERS			TAU only		
	n	Mean (SD)	n	Mean (SD)	Adjusted mean difference (95% CI)	p value	n	r	p value	n	r	p value
CompACT total	201	−12.23 (20.35)	193	−4.41 (15.46)	−1.80 (−2.59, −1.01)	<0.0001	200	0.38	<0.0001	193	0.36	<0.0001
CompACT OE	201	−6.82 (9.97)	194	−2.64 (7.25)	−1.92 (−2.75, −1.09)	<0.0001	200	0.31	<0.0001	194	0.30	<0.0001
CompACT BA	201	−3.52 (6.52)	193	−1.19 (5.39)	−1.91 (−2.70, −1.11)	<0.0001	200	0.34	<0.0001	193	0.27	0.0001
CompACT VA	201	−1.89 (9.43)	194	−0.57 (8.31)	−2.39 (−3.21, −1.57)	<0.0001	200	0.25	0.0003	194	0.23	0.0011

	Change scores from baseline to 24 weeks						Correlations with GAD-7 at 24 weeks					
	iACT4CARERS		TAU only		Difference with covariates		iACT4CARERS			TAU only		
	n	Mean (SD)	n	Mean (SD)	Adjusted mean difference (95% CI)	p value	n	r	p value	n	r	p value
CompACT total	185	−12.17 (21.90)	192	−3.28 (15.98)	−1.34 (−2.20, −0.48)	0.0023	185	0.37	<0.0001	192	0.32	<0.0001
CompACT OE	185	−6.84 (10.88)	193	−2.66 (8.58)	−1.53 (−2.4, −0.65)	0.0007	185	0.32	<0.0001	193	0.29	0.0001
CompACT BA	185	−3.51 (6.98)	192	−0.75 (5.77)	−1.44 (−2.31, −0.57)	0.0012	185	0.30	<0.0001	192	0.27	0.0002
CompACT VA	185	−1.82 (9.15)	193	0.18 (8.24)	−1.90 (−2.79, −1.01)	<0.0001	185	0.28	0.0486	193	0.14	0.0001

CompACT = Comprehensive Assessment of Acceptance and Commitment Therapy processes. CompACT OE = CompACT Openness to Experience. CompACT BA = CompACT Behavioural Awareness. CompACT VA = CompACT Valued Action. GAD-7 = Generalized Anxiety Disorder-7. iACT4CARERS = Internet-delivered Self-help Acceptance and Commitment Therapy for Family Carer of People Living with Dementia. TAU = Treatment as Usual.

Mediation analysis showed a significant indirect effect of the intervention on 24-week GAD-7 scores through change in CompACT from baseline to 12 weeks (−0.56 points; bootstrap SE 0.18; 95% bootstrap CI −0.90 to −0.21; p = 0.002), indicating that improvements in psychological flexibility statistically mediated treatment effects (Appendix 18). The estimated proportion of the total effect mediated was 20.2% (bootstrap SE 6.67; 95% bootstrap CI 7.16% to 33.32%; p = 0.002). Using a MCID defined as at least a 20% reduction in GAD-7 scores from baseline, the intervention was associated with a substantially higher probability of achieving the MCID than control at both 12 weeks and 24 weeks (77.1% [95% CI 70.8–82.4] versus 51.8% [95% CI 44.8–58.7] at 12 weeks; Appendix 19). Consistent with these findings, the distribution of GAD-7 severity categories was more favourable in the intervention group than in the control group, with a greater proportion of participants shifting towards lower severity at 12 weeks and a sustained shift in a favourable direction at 24 weeks (severe baseline symptoms remaining in the severe category at 12 weeks: 16.0% versus 41.7%; Appendix 20).

## Discussion

This is the first adequately powered RCT of ACT for family carers of people living with dementia. Internet-delivered ACT tailored for dementia carers, delivered as self-help with minimal therapist support (iACT4CARERS) plus treatment-as-usual, reduced anxiety symptoms compared with treatment-as-usual alone at 12 weeks post-randomisation, with effects maintained at 24 weeks (effect sizes 0.53 and 0.42, respectively). The primary outcome (anxiety at 12 weeks) remained robust across all sensitivity analyses. Beyond statistical significance, the findings were clinically meaningful, with a greater proportion of participants in the intervention group achieving clinically important reductions in anxiety symptoms. The trial did not include an actual bona fide alternative treatment as a comparator; therefore, the extent to which the observed effects are specific to ACT or shared across other therapeutic approaches cannot be directly determined. However, reductions in anxiety were associated with a greater number of sessions completed and increased psychological flexibility from baseline to 12 and 24 weeks, a core process targeted in ACT, suggesting that both treatment engagement and changes in underlying psychological processes contributed to improvements, as theoretically hypothesised.

Our findings provide robust evidence that iACT4CARERS effectively reduces anxiety in this population, a notable contrast to a recent meta-analysis of online interventions that identified only three multicomponent psychoeducational programmes targeting anxiety and reported a small effect relative to non-active controls.^8^^,^^34^ Furthermore, a meta-analysis of self-help online ACT across diverse populations reported a small effect on anxiety symptoms compared with non-active controls (g = 0.30).^35^ Although this effect should be interpreted cautiously, as the meta-analysis included both guided and unguided interventions, the effect sizes observed in the current trial are comparable to, or exceed, those reported in broader populations in the ACT literature, supporting the clinical meaningfulness of the effect observed in this study. This finding is clinically and socially important given the high prevalence of anxiety among family carers of people living with dementia.

Similar benefits were observed for the secondary outcome of depressive symptoms at 12- and 24-weeks post-randomisation (effect sizes 0.44 and 0.40). Previous meta-analyses of dementia carer interventions, largely focused on conventional CBT, have shown consistent effects on depression but not anxiety.^6^^,^^7^ The multidomain effects observed in this trial are therefore particularly important, given the high prevalence of comorbid anxiety and depression,^36^ and the increased risk of suicide associated with such comorbidity among family carers of people living with dementia.^37^

Our findings provide high-quality evidence for the clinical effectiveness of ACT for family carers of people with dementia, building on previously underpowered studies. Current UK National Institute for Health and Care Excellence (NICE) dementia guidelines note carers’ increased risk of depression but do not address anxiety and recommend applying general guidance for treating depression without tailoring it for dementia carers. While CBT has demonstrated efficacy for depressive symptoms in this population, there is no clear evidence for other therapeutic approaches referenced in such general guidance (e.g., counselling).

In our feasibility study,^13^ therapists spent a mean of 20 min per session providing written feedback; including two optional 30-min calls, maximum therapist input was estimated at 3.6 h per participant, although not all participants completed all sessions or calls. This is likely less resource-intensive than standard face-to-face psychological therapies delivered by highly trained clinicians. AI-assisted feedback drafting could further reduce therapist input, although safety requires further evaluation. iACT4CARERS also offers flexible delivery within routine clinical workflows while providing meaningful benefits to carers. This trial provides the first robust evidence that ACT can reduce both anxiety and depressive symptoms in family carers of people living with dementia, even when delivered as low-intensity self-help, addressing a key gap in clinical guidance. This explanatory RCT recruited self-referring community participants beyond standard clinician referral routes, with intervention delivery, therapist support, and clinical oversight coordinated centrally. Completion rates were high, and therapists from diverse backgrounds across 20 NHS sites delivered the intervention with timely feedback. These findings suggest the potential value of a centralised delivery model within a national healthcare system to support family carers, particularly in remote or underserved areas in the UK and elsewhere. Future research in real-world settings should focus on sustainable centralised funding models to enable implementation at scale.

This trial has several limitations. The sample self-identified predominantly as White, female, and highly educated, with 8% from non-White ethnic groups. While this broadly reflects the current UK dementia population, where around 6% of people with recorded dementia are from non-White backgrounds,^38^ male carers, carers from ethnic minority groups, and those with lower educational attainment were less well represented, suggesting that remote delivery alone may not ensure equitable reach. Further research is needed to improve access and engagement among these subgroups of carers to support successful scale-up. Follow-up was limited to 24 weeks, limiting insights into longer-term effects. Given carers’ fluctuating needs, particularly at key transition points (e.g., transition to residential care), future studies should assess long-term outcomes and the potential role of booster sessions. In exploratory analyses, therapist fidelity, assessed using the ACT-FM (a measure designed for in-person therapy), was not associated with anxiety symptoms at 12 weeks. Scores were low, as expected, since written feedback focused on engagement rather than therapy delivery, likely explaining the non-significant correlations. Future research could explore measures of therapist interaction quality specifically tailored to guided self-help interventions. Finally, safety data should be interpreted cautiously, as intervention participants had weekly therapist contact, whereas controls were contacted only for follow-up, and the trial did not capture safety issues beyond adverse event monitoring, such as temporary deterioration.

In conclusion, iACT4CARERS plus treatment-as-usual was effective in reducing anxiety and depressive symptoms among family carers of people living with dementia at both 12- and 24-weeks post-randomisation, compared with treatment-as-usual alone. The significant reduction in anxiety symptoms—the primary outcome—was clinically meaningful and was associated with treatment engagement and changes in underlying psychological processes, specifically psychological flexibility. This accessible, low-resource self-help intervention, requiring minimal non-expert therapist input, has strong potential for scalable community implementation and for addressing a major unmet mental health need in this population; further evaluation is warranted.

## Contributors

NK, RLG, MK, LM, DT, ER, BC, MH, AN, and MF contributed to trial conceptualisation and funding acquisition. NK, RLG, MK, LM, PA, DT, MH, and MF contributed to methodology development. NK, EF, PA, and BC contributed to project administration. NK, RLG, PA, MH, and MF contributed to supervision. NK, MR, APT, DH, and AC contributed to investigation. NK, EF, and CG contributed to data curation. NK, RLG, LM, ALB, ICL, and LGM contributed to therapist fidelity investigation. CG contributed to programming of the database. MK and RV contributed to formal analysis and verified the underlying data. NK contributed to writing of the original manuscript. All authors contributed to manuscript review and editing. All authors had full access to all study data and confirm that they take responsibility for the decision to submit it for publication.

## Data sharing statement

De-identified participant data will be made available upon request following publication of the study results. Requests should be directed to the corresponding author and include the specific data fields required and the purpose of the request, ideally accompanied by a detailed protocol or, at minimum, a research plan. The data dictionary can also be provided. The open-access study protocol has been published, and the statistical analysis plan is available on the trial registry website. Requests will be reviewed on a case-by-case basis, and requestors will be required to complete a data-sharing agreement with the study sponsor prior to data transfer.

## Declaration of interests

NK, RLG, MK, LM, DT, ER, BC, MH, AN, and MF declare institutional financial support from the grant for the submitted work (National Institute for Health and Care Research Health Technology Assessment NIHR150071). All other authors declare no competing interests.

## Appendix A Supplementary data

Appendix

Protocol
