# Supplementary material for: Clinical effectiveness of internet-delivered self-help Aacceptance and Commitment Therapy for family carers of people with dementia (iACT4CARERS): a multicentre, parallel, randomised controlled trial
Source: Lancet Reg Health Eur. 2026 Jul 11;67:101766. doi: 10.1016/j.lanepe.2026.101766 (PMC13453953; doi:10.1016/j.lanepe.2026.101766)
Supplement: Supplementary file 1 [file mmc1.pdf]

iACT4CARERS Appendices

Clinical effectiveness of internet-delivered self-help Acceptance and Commitment Therapy for family carers of people with dementia (iACT4CARERS): a multicentre, parallel, randomised controlled trial

Open access study protocol: https://doi.org/10.1016/j.cct.2024.107685

Open access Statistical Analysis Plan (SAP)*: https://www.isrctn.com/ISRCTN45995725

Open access Health Economic Analysis Plan (HEAP)*: https://www.isrctn.com/ISRCTN45995725

*See the registry's ***Additional Files*** section.

iACT4CARERS Appendices

Contents

Appendix 1: Baseline health-related quality of life measure (EQ-5D-5L) for health economic analysis 2
Appendix 2: Baseline capability measure (ICECAP-O) for health economic analysis 3
Appendix 3: Baseline modified Client Service Receipt Inventory (CSRI) measure – Caring activities 4
Appendix 4: Baseline and 12-week modified CSRI measure – Use of health and social care services 5
Appendix 5: Baseline and 12-week modified CSRI measure – Use of dementia related support and advice 6
Appendix 6: Likelihood ratio (LR) tests for random effects for the study outcomes 7
Appendix 7: Residual distributions from adjusted regression models 8
Appendix 8: Reason for withdrawal from the study 15
Appendix 9: Treatment completion data 15
Appendix 10: Outcome completeness 16
Appendix 11: Extent and patterns of missing data 16
Appendix 12: Figures demonstrating mean outcome scores over time by group 17
Appendix 13: Safety data 18
Appendix 14: Sensitivity to missing data 18
Appendix 15: Sensitivity to inadequate compliance 19
Appendix 16: Sensitivity to referral route 19
Appendix 17: Sensitivity to treatment expectation/treatment preference 20
Appendix 18: Mediation analysis of the effect of the intervention on anxiety symptoms via change in psychological flexibility 20
Appendix 19: Minimal Clinically Important Difference (MCID) analysis 21
Appendix 20: Analysis of participant shifts across GAD-7 severity categories 22
Appendix 21: Content of each session of iACT4CARERS 23
Appendix 22: Treatment expectations and intervention preference questionnaires 24
Appendix 23: PRECIS-2 assessment of trial design 25

Appendix 1: Baseline health-related quality of life measure (EQ-5D-5L) for health economic analysis

		iACT4CARERS (N=249)	TAU only (N=247)
EQ-5D-5L Mobility	Level 1	199 (79.9%)	184 (74.5%)
	Level 2	27 (10.8%)	40 (16.2%)
	Level 3	19 (7.6%)	15 (6.1%)
	Level 4	4 (1.6%)	8 (3.2%)
	Level 5	0 (0%)	0 (0%)
EQ-5D-5L Self-care	Level 1	239 (96.0%)	227 (92.0%)
	Level 2	9 (3.6%)	16 (6.5%)
	Level 3	1 (0.4%)	4 (1.6%)
	Level 4	0 (0%)	0 (0%)
	Level 5	0 (0%)	0 (0%)
EQ-5D-5L Usual activities	Level 1	150 (60.2%)	130 (52.6%)
	Level 2	47 (18.9%)	65 (26.3%)
	Level 3	42 (16.9%)	32 (13.0%)
	Level 4	7 (2.8%)	14 (5.7%)
	Level 5	3 (1.2%)	6 (2.4%)
EQ-5D-5L Pain/discomfort	Level 1	104 (41.8%)	96 (38.9%)
	Level 2	91 (36.6%)	99 (40.1%)
	Level 3	44 (17.7%)	39 (15.8%)
	Level 4	8 (3.2%)	13 (5.3%)
	Level 5	2 (0.8%)	0 (0%)
EQ-5D-5L Anxiety/depression	Level 1	108 (43.4%)	115 (46.6%)
	Level 2	95 (38.2%)	92 (37.3%)
	Level 3	23 (9.2%)	22 (8.9%)
	Level 4	13 (5.2%)	14 (5.7%)
	Level 5	10 (4.0%)	4 (1.6%)
EQ-5D-5L VAS: Median (IQR), Min - Max		73.0 (26), 20 - 100	71.0 (30), 1 - 100

iACT4CARERS=Internet-delivered Self-help Acceptance and Commitment Therapy for Family Carer of People Living with Dementia. TAU=Treatment as Usual. Level 1 indicates no problems in carrying out the specified activities, such as self-care, or no pain/symptoms, while Level 5 indicates being unable to carry out the specified activities or experiencing extreme pain/symptoms.

Appendix 2: Baseline capability measure (ICECAP-O) for health economic analysis

		iACT4CARERS (N=249)	TAU only (N=247)
ICECAP-O Total tariff score: Mean (SD), Min - Max		0.65 (0.16), 0.11 – 0.99	0.64 (0.16), 0.11 - 0.99
ICECAP-O Love and friendship	Level 1	5 (2.0%)	11 (4.5%)
	Level 2	38 (15.3%)	31 (12.6%)
	Level 3	107 (43.0%)	92 (37.3%)
	Level 4	99 (39.8%)	113 (45.8%)
ICECAP-O Thinking about future	Level 1	4 (1.6%)	3 (1.2%)
	Level 2	13 (5.2%)	17 (6.9%)
	Level 3	87 (34.9%)	89 (36.0%)
	Level 4	145 (58.2%)	138 (55.9%)
ICECAP-O Feeling valued	Level 1	10 (4.0%)	6 (2.4%)
	Level 2	9 (3.6%)	14 (5.7%)
	Level 3	81 (32.5%)	83 (33.6%)
	Level 4	149 (59.8%)	144 (58.3%)
ICECAP-O Enjoyment and pleasure	Level 1	0 (0.0%)	2 (0.8%)
	Level 2	13 (5.2%)	10 (4.1%)
	Level 3	70 (28.1%)	64 (25.9%)
	Level 4	166 (66.7%)	171 (69.2%)
ICECAP-O Independence	Level 1	21 (8.4%)	25 (10.1%)
	Level 2	32 (12.9%)	29 (11.7%)
	Level 3	95 (38.2%)	82 (33.2%)
	Level 4	101 (40.6%)	111(44.9%)

iACT4CARERS=Internet-delivered Self-help Acceptance and Commitment Therapy for Family Carer of People Living with Dementia. ICECAP-O=ICEpop CAPability measure for Older people. TAU=Treatment as Usual. Level 1 reflects the lowest perceived capacity (unable to have the love and friendship they want), while Level 4 reflects the highest perceived capacity (feeling able to have all the love and friendship they want).

Appendix 3: Baseline modified Client Service Receipt Inventory (CSRI) measure – Caring activities

	iACT4CARERS (N=249)	TAU only (N=247)
**Daily caregiving activities**		
Personal care	116 (46.6%)	134 (54.3%)
Helping with finances	226 (90.8%)	216 (87.5%)
Practical help	214 (85.9%)	216 (87.5%)
Taking them to appointments	209 (83.9%)	212 (85.8%)
Medications	190 (76.3%)	195 (79.0%)
Keeping them company	208 (83.5%)	232 (93.9%)
Making sure they are safe	179 (71.9%)	201 (81.4%)
Other	48 (19.3%)	47 (19.0%)
**Hours of caring per day**		
No help in a typical day	0 (0%)	4 (1.6%)
Up to 1 hour	14 (5.6%)	4 (1.6%)
More than 1 hour but up to 2 hours	25 (10.0%)	24 (9.7%)
More than 2 hours but up to 3 hours	35 (14.1%)	39 (15.8%)
More than 3 hours but up to 5 hours	40 (16.1%)	30 (12.2%)
More than 5 hours but up to 10 hours	34 (13.7%)	35 (14.2%)
More than 10 hours but not overnight	11 (4.4%)	16 (6.5%)
More than 10 hours and/including overnight	68 (27.3%)	74 (30.0%)
Other	22 (8.8%)	21 (8.5%)
**Unpaid support from others**		
Currently receiving regular help from friends/relatives (Yes)	105 (42.2%)	119 (48.2%)
Number of hours of help from them per week: Median (IQR), Min-Max	7 (19), 1 - 168	10 (21), 1 - 168
**Time taken off from paid work in the last four weeks**		
I had to take time off work	51 (20.5%)	43 (17.4%)
I did not have to take time off work	49 (19.7%)	48 (19.4%)
I do not work	149 (59.8%)	156 (63.2%)
Number of whole days taken time off work: Median (IQR), Min-Max	2 (4), 0 - 28	2 (3), 0 -28
Number of time part of a day taken off work: Median (IQR), Min-Max	2 (3), 0 - 20	3 (5), 0 - 28

iACT4CARERS=Internet-delivered Self-help Acceptance and Commitment Therapy for Family Carer of People Living with Dementia. CSRI= Client Service Receipt Inventory. TAU=Treatment as Usual.

Appendix 4: Baseline and 12-week modified CSRI measure – Use of health and social care services

Carers' use of health and social care services over the last three months

	Baseline		12 weeks	
	**iACT4CARERS (N=249)**	**TAU only (N=247)**	**iACT4CARERS (N=200)**	**TAU only (N=193)**
**Inpatient and outpatient hospital use (% Yes)**				
Stayed in hospital overnight	6 (2.4%)	7 (2.8%)	3 (1.5%)	3 (1.6%)
Been to accident and emergency (casualty)	14 (5.6%)	18 (7.3%)	7 (3.5%)	17 (8.8%)
Attended a hospital outpatient appointment	77 (30.9%)	86 (34.8%)	66 (33.0%)	63 (32.6%)
None of the above	163 (65.5%)	153 (61.9%)	129 (64.5%)	117 (60.6%)
**Other outpatient service use (% Yes)**				
GP at surgery/by phone/online	141 (56.6%)	141 (57.1%)	112 (56.0%)	106 (54.9%)
GP at home	0 (0%)	0 (0%)	0 (0%)	0 (0%)
Practice nurse at surgery/by phone/online	38 (15.3%)	49 (19.6%)	35 (17.5%)	48 (24.9%)
Mental health nurse	5 (2.0%)	4 (1.6%)	4 (2.0%)	5 (2.6%)
Psychiatrist	1 (0.4%)	0 (0%)	1 (0.5%)	0 (0%)
Psychologist	1 (0.4%)	1 (0.4%)	3 (1.5%)	0 (0%)
Social worker or care manager	4 (1.6%)	8 (3.2%)	9 (4.5%)	11 (5.7%)
Occupational therapist	0 (0%)	4 (1.6%)	3 (1.5%)	3 (1.6%)
District nurse/community nurse	5 (2.0%)	2 (0.8%)	3 (1.5%)	3 (1.6%)
Counsellor	4 (1.6%)	2 (0.8%)	7 (3.5%)	4 (2.1%)
Mental health team worker	6 (2.4%)	4 (1.6%)	8 (4.0%)	9 (4.7%)
Other	29 (11.7%)	23 (9.3%)	27 (13.5%)	24 (12.4%)
None of the above	84 (33.7%)	85 (34.4%)	63 (31.5%)	62 (32.1%)
**Mental health treatment (% Yes)**				
Medications for mental health	51 (20.5%)	49 (19.8%)	41 (20.5%)	37 (19.2%)
Psychotherapy received – Cognitive behaviour therapy	2 (0.8%)	1 (0.4%)	1 (0.5%)	1 (0.5%)
Psychotherapy received – Counselling	1 (0.4%)	1 (0.4%)	1 (0.5%)	3 (1.6%)
Psychotherapy received – Other	0 (0%)	0 (0%)	2 (1.0%)	0 (0%)
Number of sessions received: Median (IQR), Min-Max	5.0 (4), 2 - 6	5.5 (9), 1 - 10	3.0 (3), 2 - 6	3.0 (3), 2-5

iACT4CARERS=Internet-delivered Self-help Acceptance and Commitment Therapy for Family Carer of People Living with Dementia. CSRI= Client Service Receipt Inventory. TAU=Treatment as Usual.

Appendix 5: Baseline and 12-week modified CSRI measure – Use of dementia related support and advice

Use of dementia related support and advice over the last three months

	Baseline		12 weeks	
	**iACT4CARERS (N=249)**	**TAU only (N=247)**	**iACT4CARERS (N=200)**	**TAU only (N=193)**
**Dementia advice and education from professionals**				
GP	12 (4.8%)	21 (8.5%)	9 (4.5%)	11 (5.7%)
Community psychiatric nurse (memory nurse)	26 (10.4%)	18 (7.3%)	18 (9.0%)	11 (5.7%)
Admiral nurse	29 (11.7%)	23 (9.3%)	17 (8.5%)	16 (8.3%)
Social worker	10 (4.0%)	19 (7.7%)	12 (6.0%)	13 (6.7%)
Other	57 (22.9%)	52 (21.1%)	42 (21.0%)	30 (15.5%)
None of the above	141 (56.6%)	151 (61.1%)	126 (63.0%)	127 (65.8%)
**Dementia support received**				
Respite care (short term stay in a care home)	7 (2.8%)	12 (4.9%)	10 (5.0%)	13 (6.7%)
Day care centre (respite without overnight stay)	17 (6.8%)	28 (11.3%)	19 (9.5%)	22 (11.4%)
Sitting service at home (no personal care)	23 (9.2%)	19 (7.7%)	23 (11.5%)	24 (12.4%)
Support worker at home (providing personal care)	29 (11.7%)	20 (8.1%)	23 (11.5%)	18 (9.3%)
Carer support group	49 (19.7%)	43 (17.4%)	35 (17.5%)	36 (18.7%)
Dementia café	41 (16.5%)	50 (20.2%)	32 (16.0%)	46 (23.8%)
Lunch club	11 (4.4%)	7 (2.8%)	14 (7.0%)	8 (4.1%)
Other	53 (21.3%)	50 (20.2%)	53 (26.5%)	23 (11.9%)
None of the above	104 (41.8%)	103 (41.7%)	76 (38.0%)	74 (38.3%)

iACT4CARERS=Internet-delivered Self-help Acceptance and Commitment Therapy for Family Carer of People Living with Dementia. CSRI= Client Service Receipt Inventory. TAU=Treatment as Usual.

Appendix 6: Likelihood ratio (LR) tests for random effects for the study outcomes

Study Outcomes	Random effect	Variance (SD)	ICC	LR Chi-squared (df)	p-value
GAD-7 at 12 weeks	Site	<0.001 (<0.001)	<0.001	0 (1)	>0.9999
	Therapist (within site)	1.14 (1.07)	0.06	1.62 (1)	0.2031
GAD-7 at 24 weeks	Site	<0.001 (<0.001)	<0.001	0 (1)	>0.9999
	Therapist (within site)	<0.001 (<0.001)	<0.001	0 (1)	>0.9999
PHQ-9 total score at 12 weeks	Site	<0.001 (<0.001)	<0.001	0 (1)	>0.9999
	Therapist (within site)	0.17(1.19)	0.008	0.03 (1)	0.8703
PHQ-9 total score at 24 weeks	Site	<0.001 (<0.001)	<0.001	0 (1)	>0.9999
	Therapist (within site)	0.05 (1.02)	0.002	0 (1)	>0.9999
CompACT total score at 12 weeks	Site	<0.001 (<0.001)	<0.001	0 (1)	>0.9999
	Therapist (within site)	<0.001 (<0.001)	<0.001	0 (1)	>0.9999
CompACT total score at 24 weeks	Site	<0.001 (<0.001)	<0.001	0 (1)	>0.9999
	Therapist (within site)	<0.001 (<0.001)	<0.001	0 (1)	>0.9999
CompACT OE score at 12 weeks	Site	<0.001 (<0.001)	<0.001	0 (1)	>0.9999
	Therapist (within site)	<0.001 (<0.001)	<0.001	0 (1)	>0.9999
CompACT OE score at 24 weeks	Site	<0.001 (<0.001)	<0.001	0 (1)	>0.9999
	Therapist (within site)	<0.001 (<0.001)	<0.001	0 (1)	>0.9999
CompACT BA score at 12 weeks	Site	0.38 (0.56)	0.013	0.63 (1)	0.4270
	Therapist (within site)	<0.001 (<0.001)	0.013	0 (1)	>0.9999
CompACT BA score at 24 weeks	Site	<0.001 (<0.001)	<0.001	0 (1)	0.9744
	Therapist (within site)	<0.001 (<0.001)	<0.001	0 (1)	0.9994
CompACT VA score at 12 weeks	Site	<0.001 (<0.001)	<0.001	0 (1)	>0.9999
	Therapist (within site)	<0.001 (<0.001)	<0.001	0 (1)	>0.9999
CompACT VA score at 24 weeks	Site	<0.001 (<0.001)	<0.001	0 (1)	>0.9999
	Therapist (within site)	<0.001 (<0.001)	<0.001	0 (1)	>0.9999
EACQ total score at 12 weeks	Site	<0.001 (<0.001)	<0.001	0 (1)	>0.9999
	Therapist (within site)	<0.001 (<0.001)	<0.001	0 (1)	>0.9999
EACQ total score at 24 weeks	Site	<0.001 (<0.001)	<0.001	0 (1)	>0.9999
	Therapist (within site)	2.10 (3.50)	0.043	0.40 (1)	0.5286
EACQ score AAB at 12 weeks	Site	<0.001 (<0.001)	<0.001	0 (1)	>0.9999
	Therapist (within site)	<0.001 (<0.001)	<0.001	0 (1)	>0.9999
EACQ score AAB at 24 weeks	Site	<0.001 (<0.001)	<0.001	0 (1)	>0.9999
	Therapist (within site)	0.82 (1.03)	0.053	0.69 (1)	0.4051
EACQ score INTE at 12 weeks	Site	<0.001 (<0.001)	<0.001	0 (1)	>0.9999
	Therapist (within site)	<0.001 (<0.001)	<0.001	0 (1)	>0.9999
EACQ score INTE at 24 weeks	Site	<0.001 (<0.001)	<0.001	0 (1)	>0.9999
	Therapist (within site)	<0.001 (<0.001)	<0.001	0 (1)	>0.9999
EACQ score ACNIE at 12 weeks	Site	0.03 (0.13)	0.004	0.06 (1)	0.8143
	Therapist (within site)	<0.001 (<0.001)	0.004	0 (1)	>0.9999
EACQ score ACNIE at 24 weeks	Site	0.006 (0.113)	0.0009	0 (1)	0.9579
	Therapist (within site)	<0.001 (<0.001)	0.0009	0 (1)	>0.9999

CompACT= Comprehensive Assessment of Acceptance and Commitment Therapy processes. CompACT OE=CompACT Openness to Experience. CompACT BA=CompACT Behavioural Awareness. CompACT VA=CompACT Valued Action. EACQ=Experiential Avoidance in Caregiving Questionnaire. EACQ AAB=EACQ Active Avoidant Behaviours. EACQ ACNIE=EACQ Apprehension Concerning Negative Internal Experiences Related to Caregiving. EACQ INTE=EACQ Intolerance of Negative Thoughts and Emotions Towards the Relative. GAD-7=Generalized Anxiety Disorder-7.

Appendix 7: Residual distributions from adjusted regression models

Residual distributions from adjusted regression models with skewness and kurtosis statistics for primary and secondary outcomes

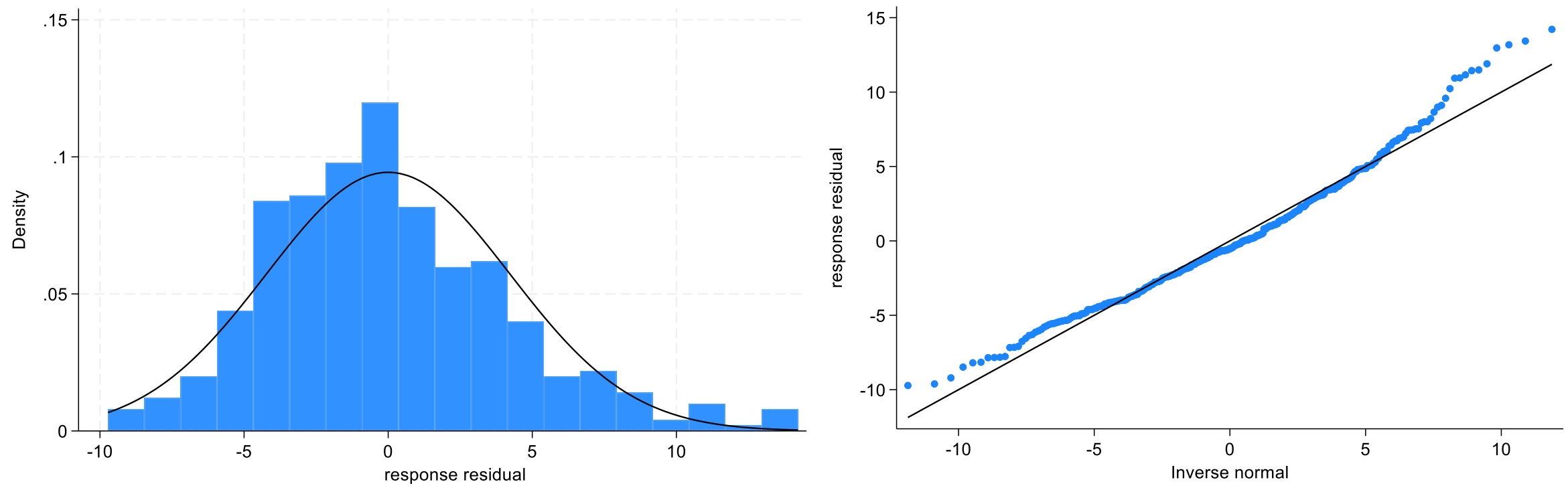

Figure 7.1: Graphical distribution of residuals from the adjusted regression models for GAD-7 at 12 weeks using histograms and normal probability plots.
Residual summary: Skewness = 0.62; Kurtosis = 3.56

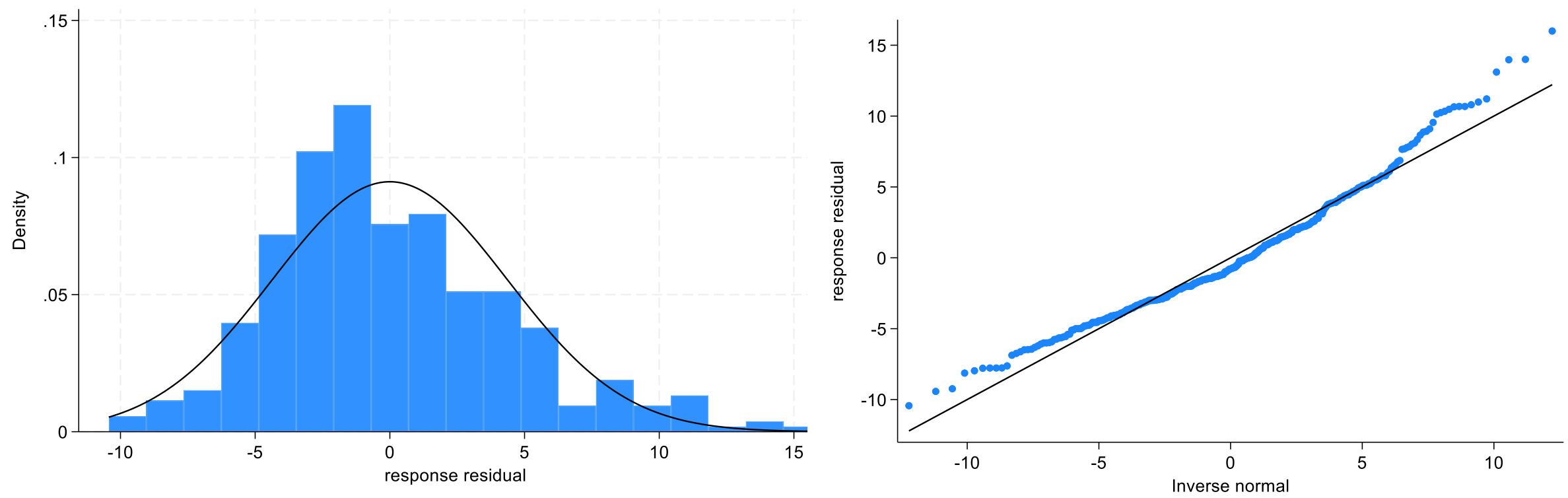

Figure 7.2: Graphical distribution of residuals from the adjusted regression models for GAD-7 at 24 weeks using histograms and normal probability plots.
Residual summary: Skewness = 0.70; Kurtosis = 3.61

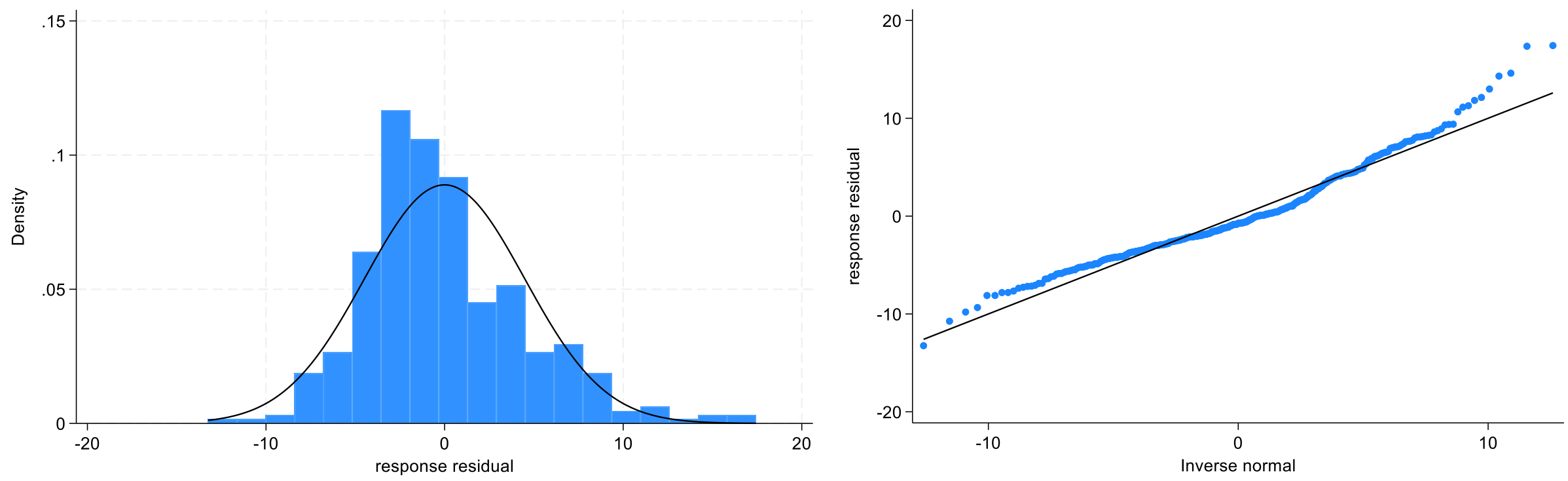

Figure 7.3: Graphical distribution of residuals from the adjusted regression models for PHQ-9 at 12 weeks using histograms and normal probability plots.
Residual summary: Skewness = 0.77; Kurtosis = 4.13

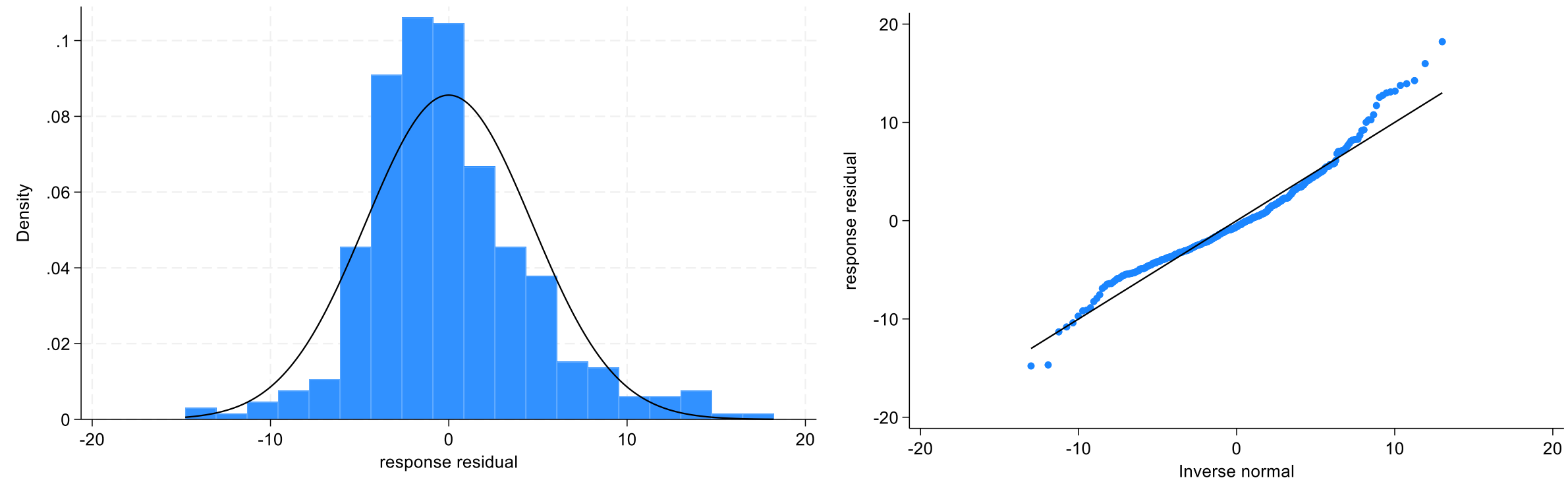

Figure 7.4: Graphical distribution of residuals from the adjusted regression models for PHQ-9 at 24 weeks using histograms and normal probability plots.
Residual summary: Skewness = 0.66; Kurtosis = 4.54

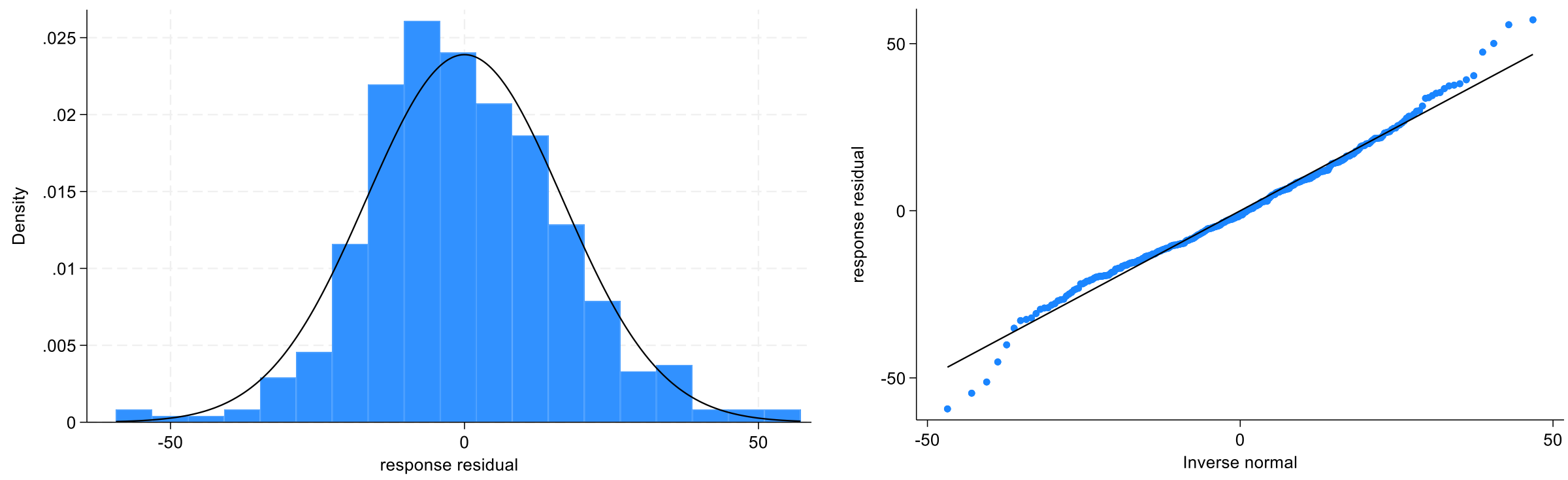

Figure 7.5: Graphical distribution of residuals from the adjusted regression models for CompACT total score at 12 weeks using histograms and normal probability plots.
Residual summary: Skewness = 0.18; Kurtosis = 3.93

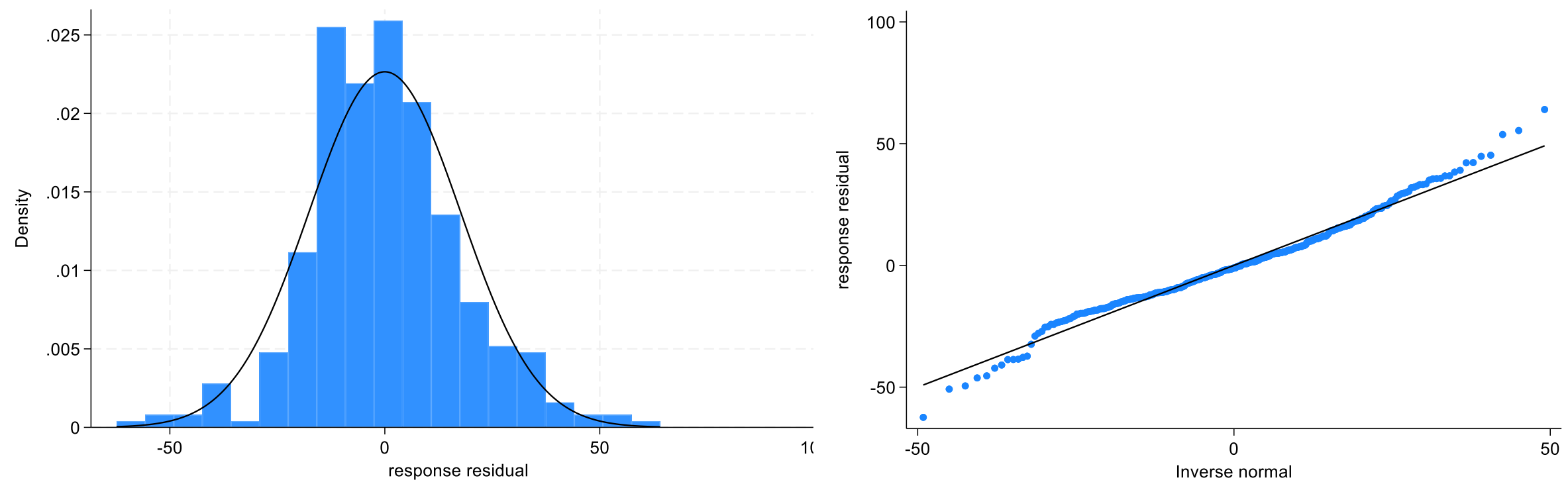

Figure 7.6: Graphical distribution of residuals from the adjusted regression models for ComPACT at 24 weeks using histograms and normal probability plots.
Residual summary: Skewness = 0.21; Kurtosis = 4.10

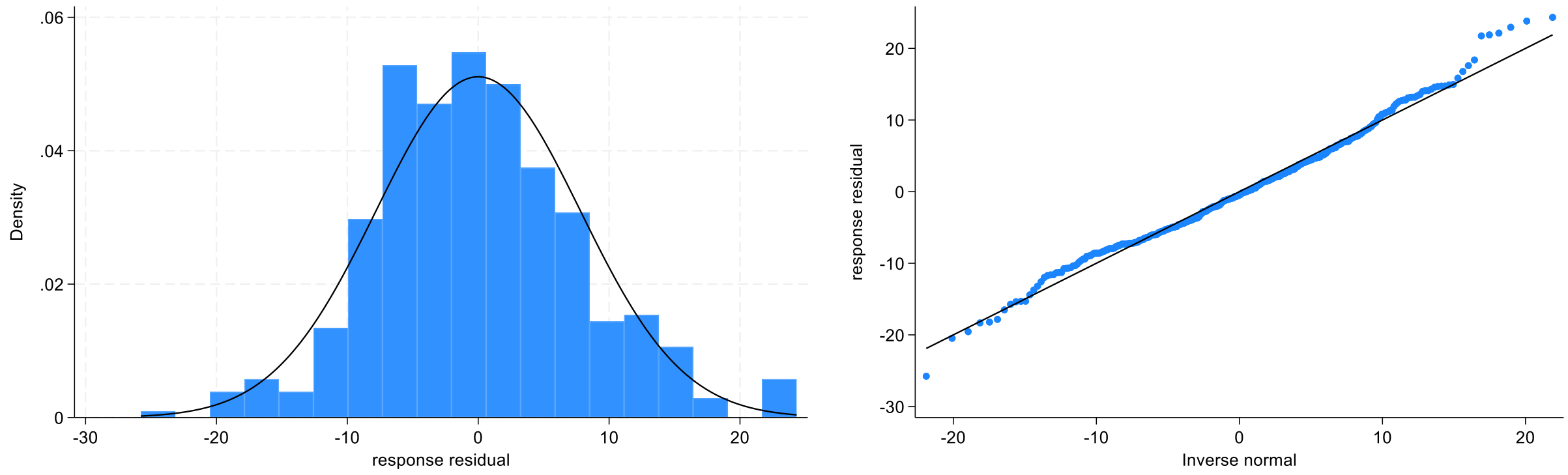

Figure 7.7: Graphical distribution of residuals from the adjusted regression models for CompACT Openness to Experience score at 12 weeks using histograms and normal probability plots.
Residual summary: Skewness = 0.28; Kurtosis = 3.47

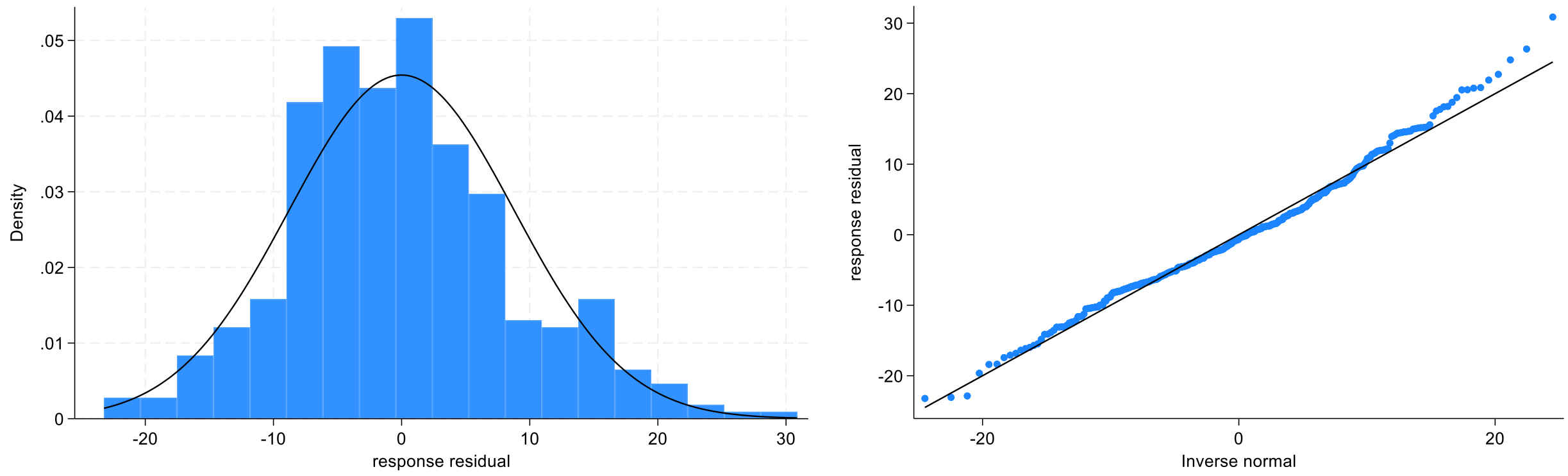

Figure 7.8: Graphical distribution of residuals from the adjusted regression models for CompACT Openness to Experience score at 24 weeks using histograms and normal probability plots.
Residual summary: Skewness = 0.37; Kurtosis = 3.42

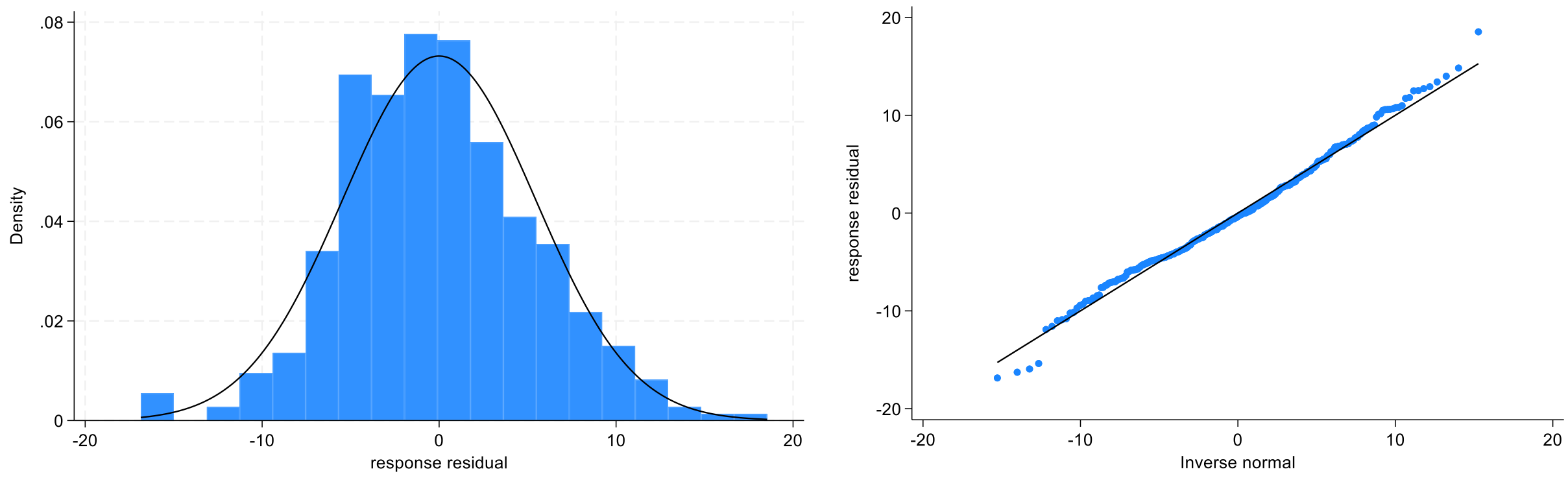

Figure 7.9: Graphical distribution of residuals from the adjusted regression models for CompACT Behavioural Awareness score at 12 weeks using histograms and normal probability plots.
Residual summary: Skewness = 0.18; Kurtosis = 3.39

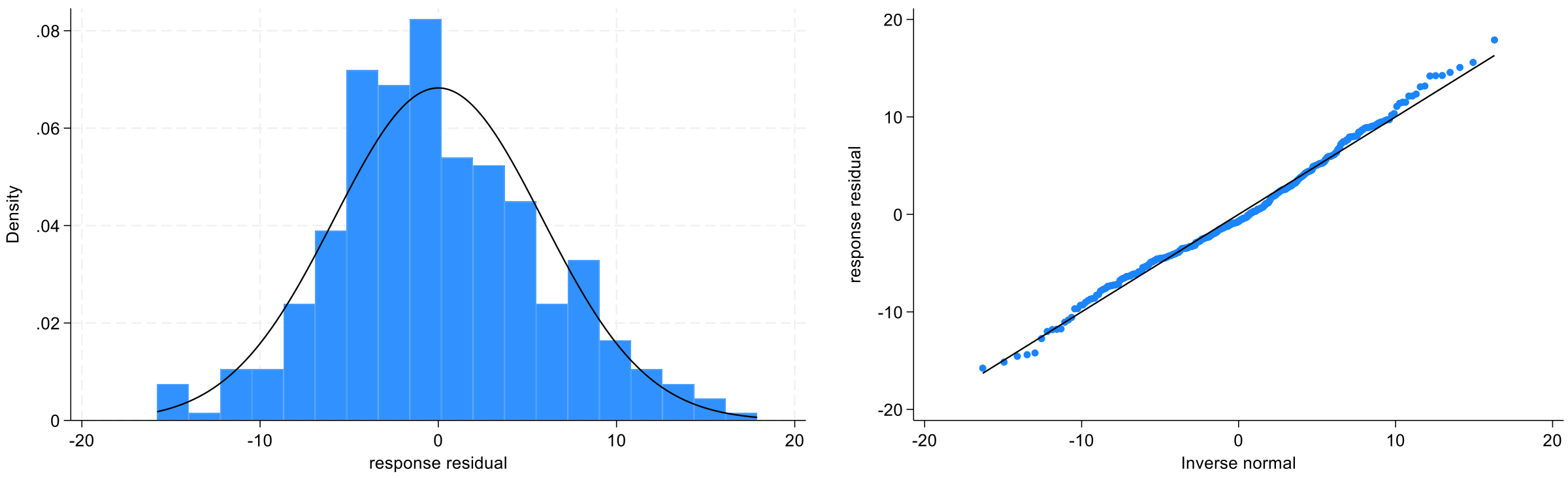

Figure 7.10: Graphical distribution of residuals from the adjusted regression models for CompACT Behavioural Awareness score at 24 weeks using histograms and normal probability plots.
Residual summary: Skewness = 0.25; Kurtosis = 3.10

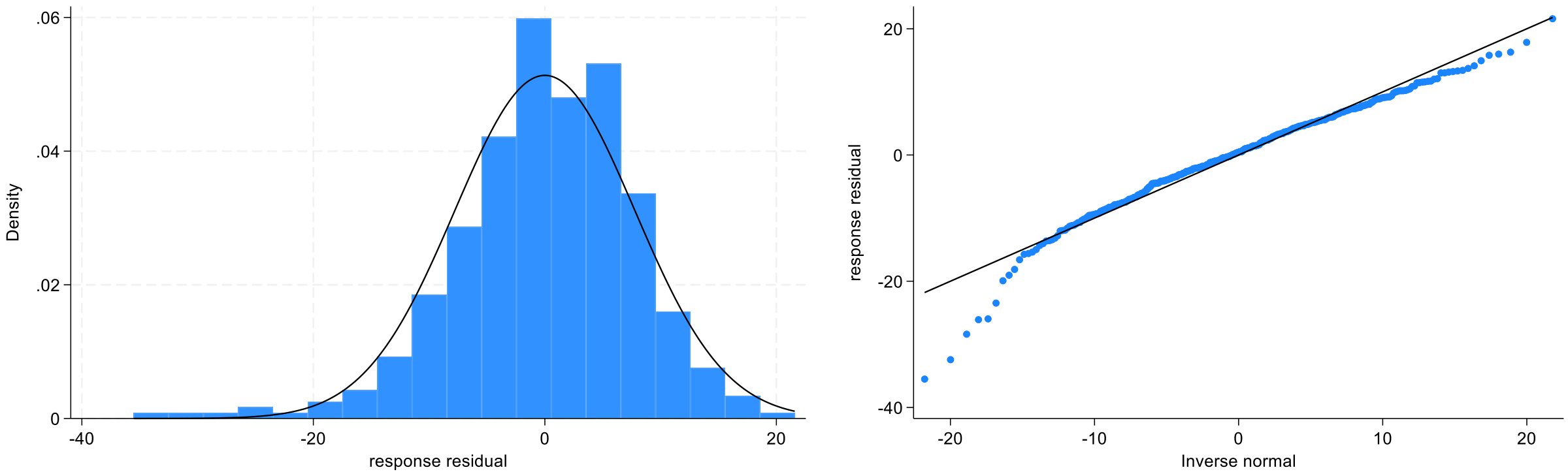

Figure 7.11: Graphical distribution of residuals from the adjusted regression models for CompACT Valued Action score at 12 weeks using histograms and normal probability plots.
Residual summary: Skewness = -0.79; Kurtosis = 5.04

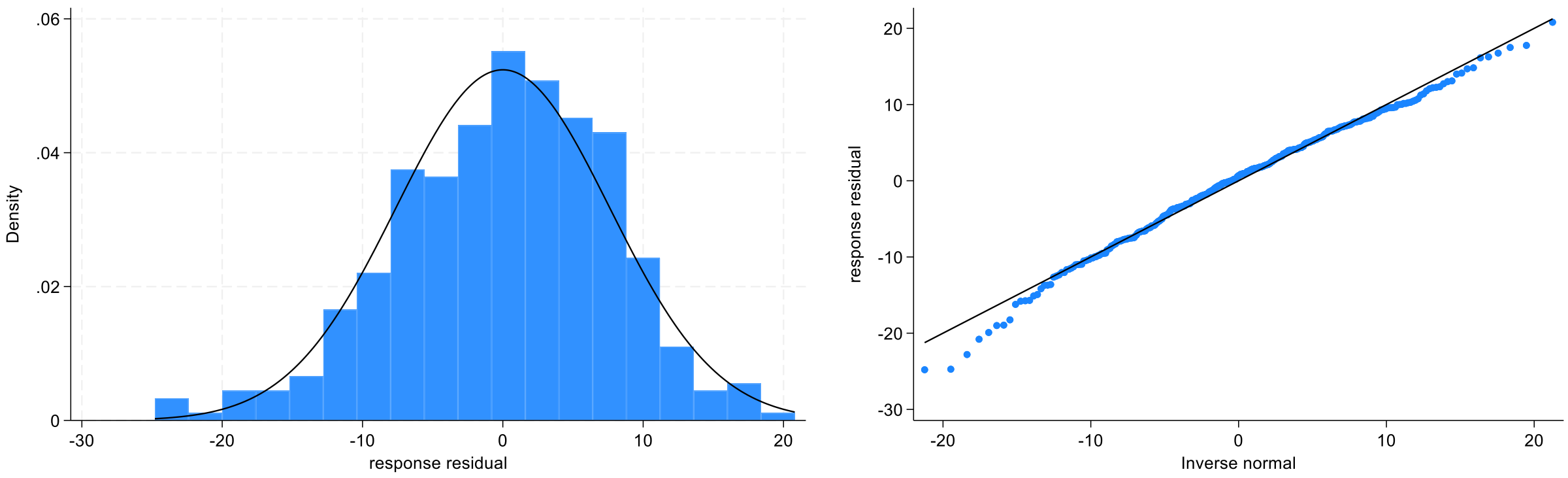

Figure 7.12: Graphical distribution of residuals from the adjusted regression models for CompACT Valued Action score at 24 weeks using histograms and normal probability plots.
Residual summary: Skewness = -0.36; Kurtosis = 3.25

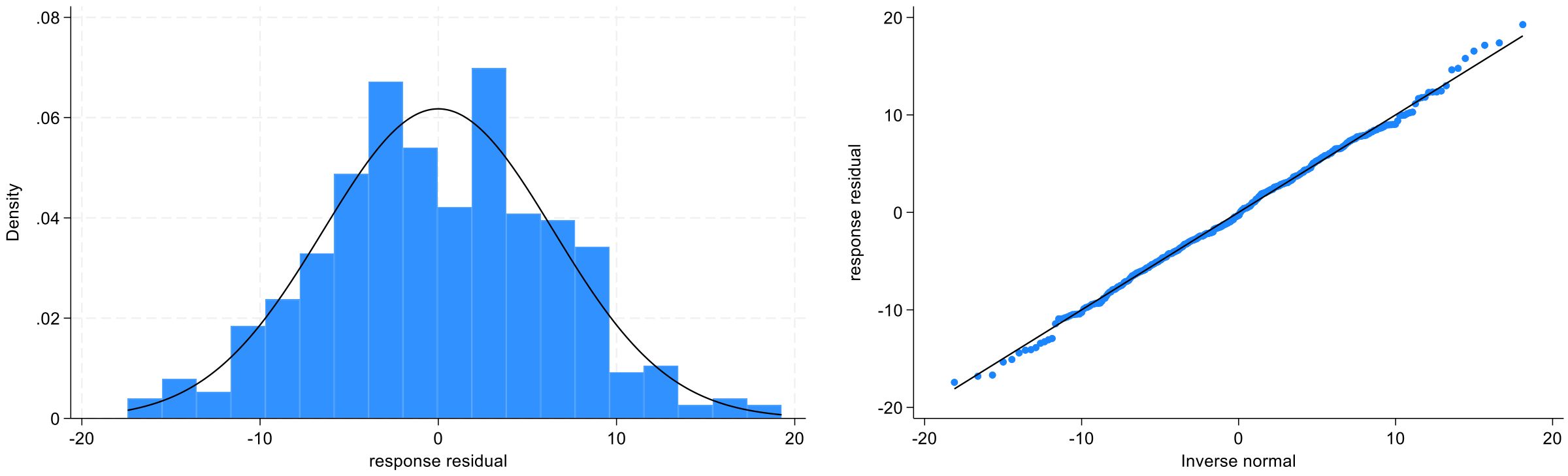

Figure 7.13: Graphical distribution of residuals from the adjusted regression models for EACQ total score at 12 weeks using histograms and normal probability plots.
Residual summary: Skewness = <0.001; Kurtosis = 2.95

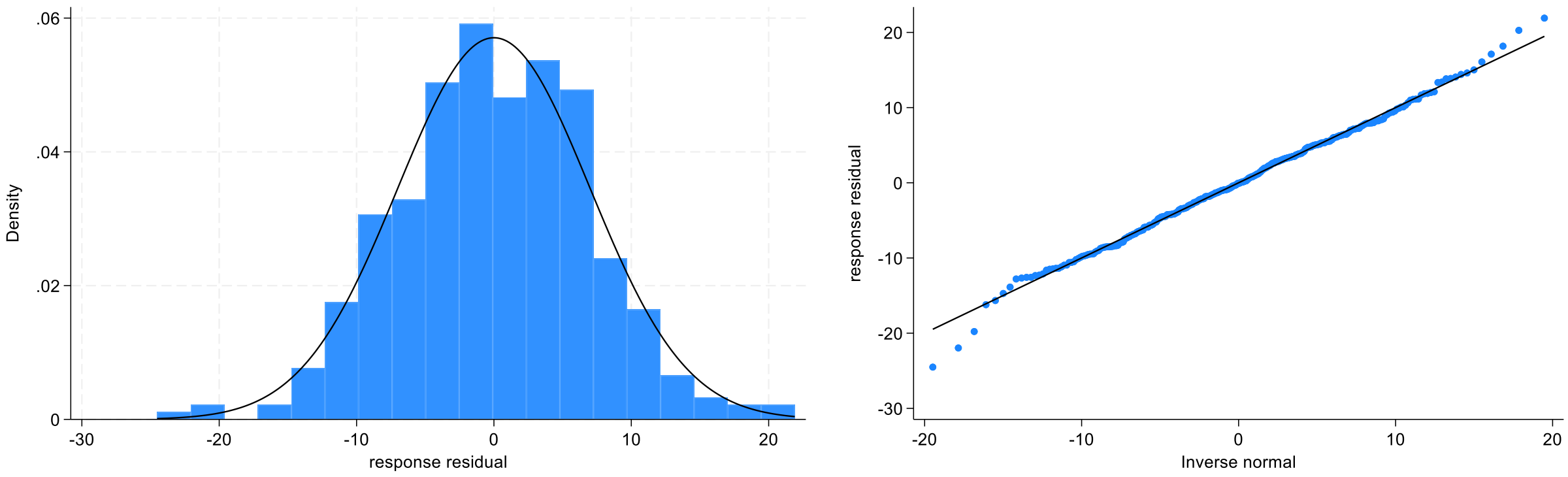

Figure 7.14: Graphical distribution of residuals from the adjusted regression models for EACQ total score at 24 weeks using histograms and normal probability plots.
Residual summary: Skewness = -0.05; Kurtosis = 3.31

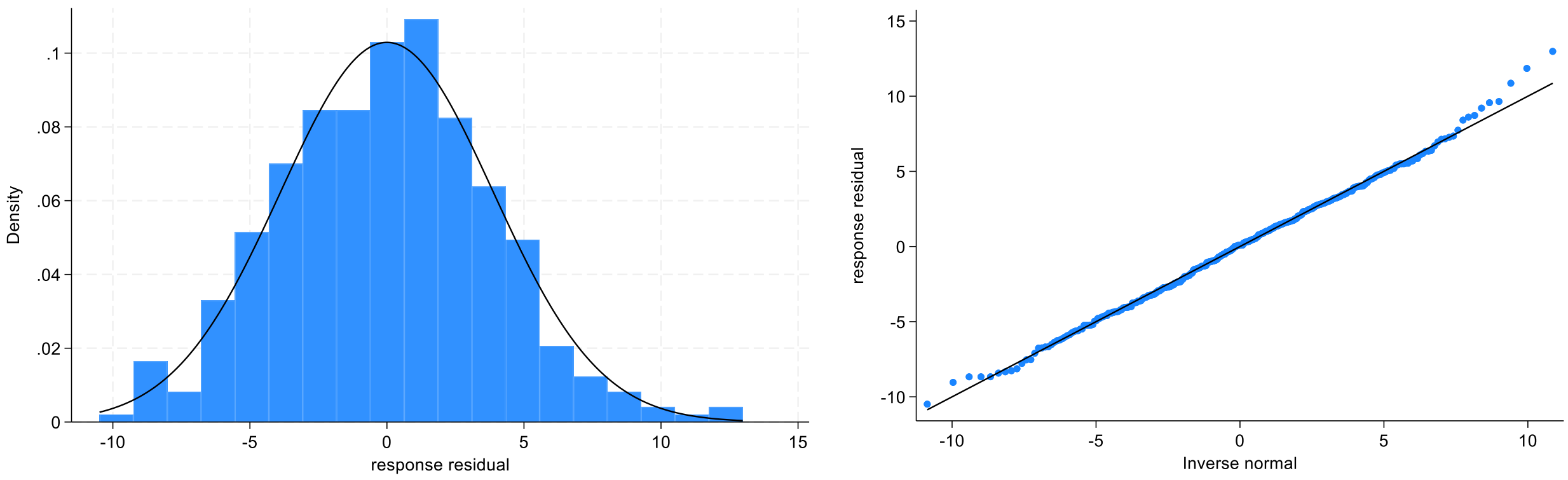

Figure 7.15: Graphical distribution of residuals from the adjusted regression models for EACQ Active Avoidant Behaviours score at 12 weeks using histograms and normal probability plots.
Residual summary: Skewness = 0.12; Kurtosis = 3.08

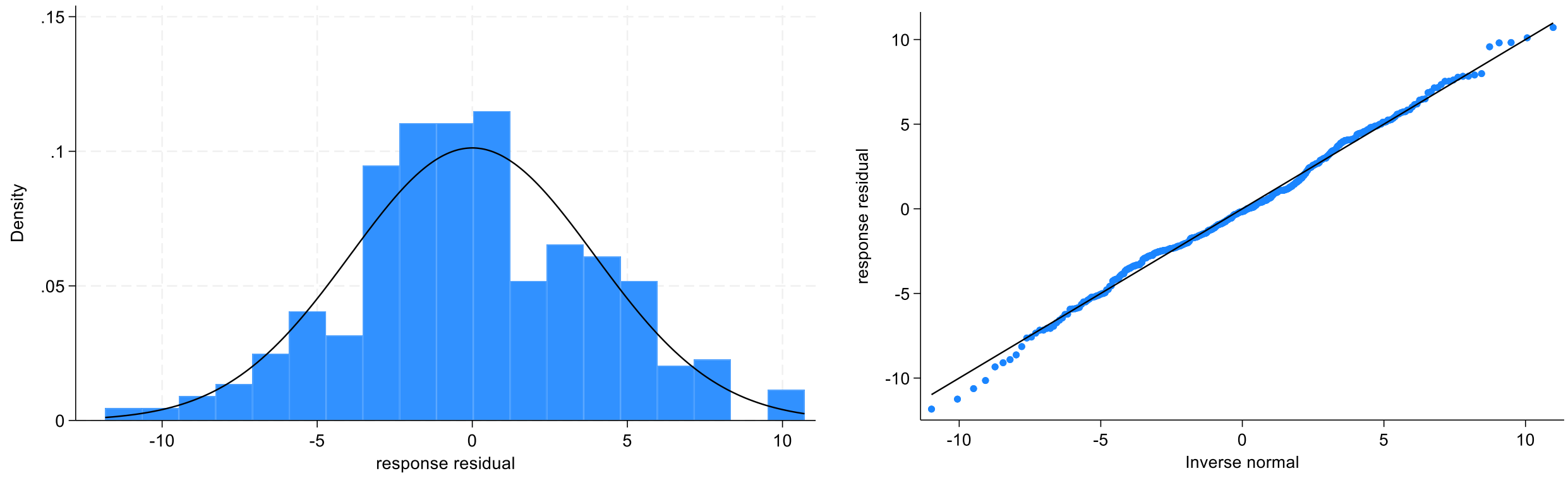

Figure 7.16: Graphical distribution of residuals from the adjusted regression models for EACQ Active Avoidant Behaviours score at 24 weeks using histograms and normal probability plots.
Residual summary: Skewness = -0.01; Kurtosis = 3.08

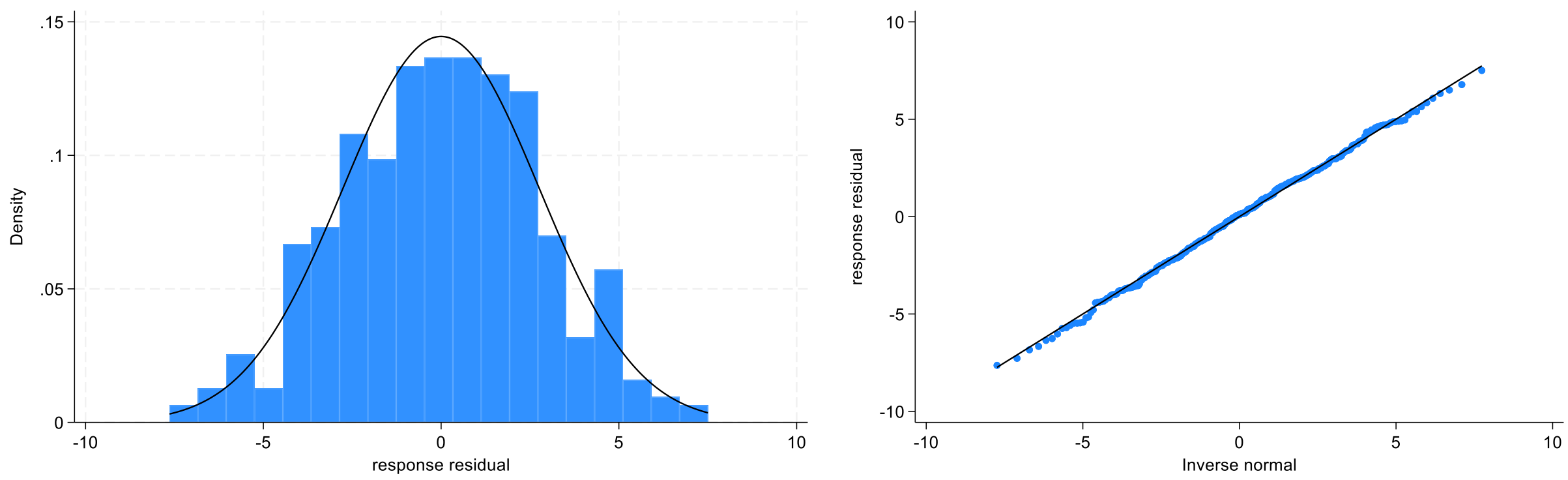

Figure 7.17: Graphical distribution of residuals from the adjusted regression models for EACQ Intolerance of Negative Thoughts and Emotions Towards the Relative score at 12 weeks using histograms and normal probability plots.
Residual summary: Skewness = -0.08; Kurtosis = 2.75

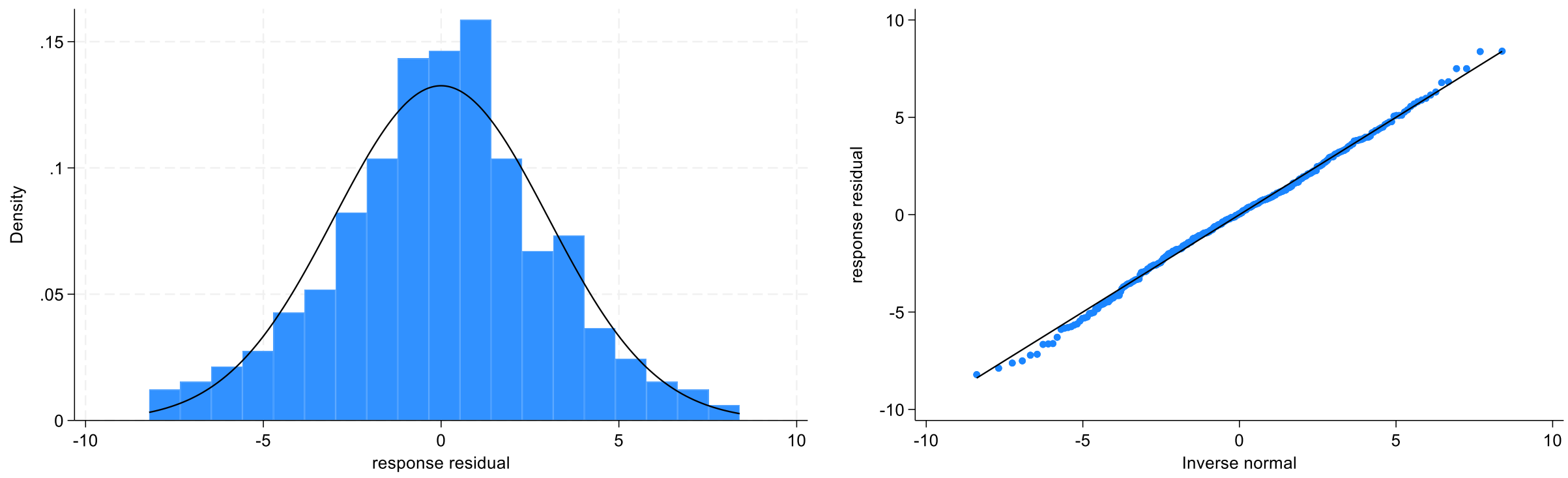

Figure 7.18: Graphical distribution of residuals from the adjusted regression models for EACQ Intolerance of Negative Thoughts and Emotions Towards the Relative score at 24 weeks using histograms and normal probability plots.
Residual summary: Skewness = -0.07; Kurtosis = 3.07

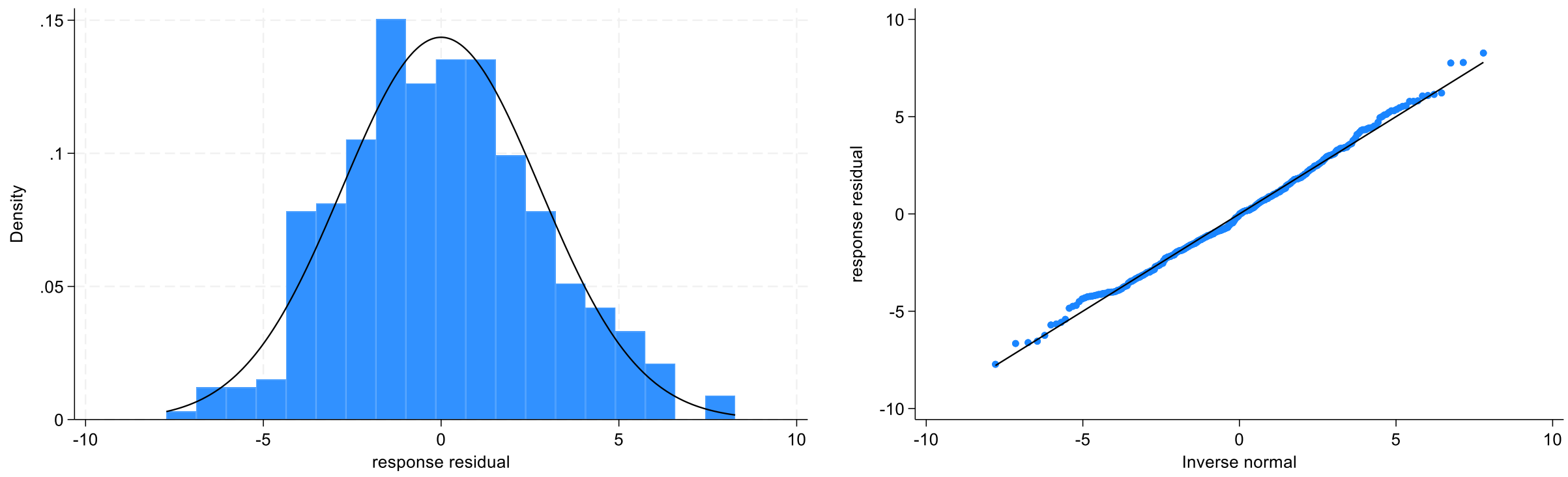

Figure 7.19: Graphical distribution of residuals from the adjusted regression models for EACQ Apprehension Concerning Negative Internal Experiences Related to Caregiving score at 12 weeks using histograms and normal probability plots.
Residual summary: Skewness = 0.19; Kurtosis = 2.86

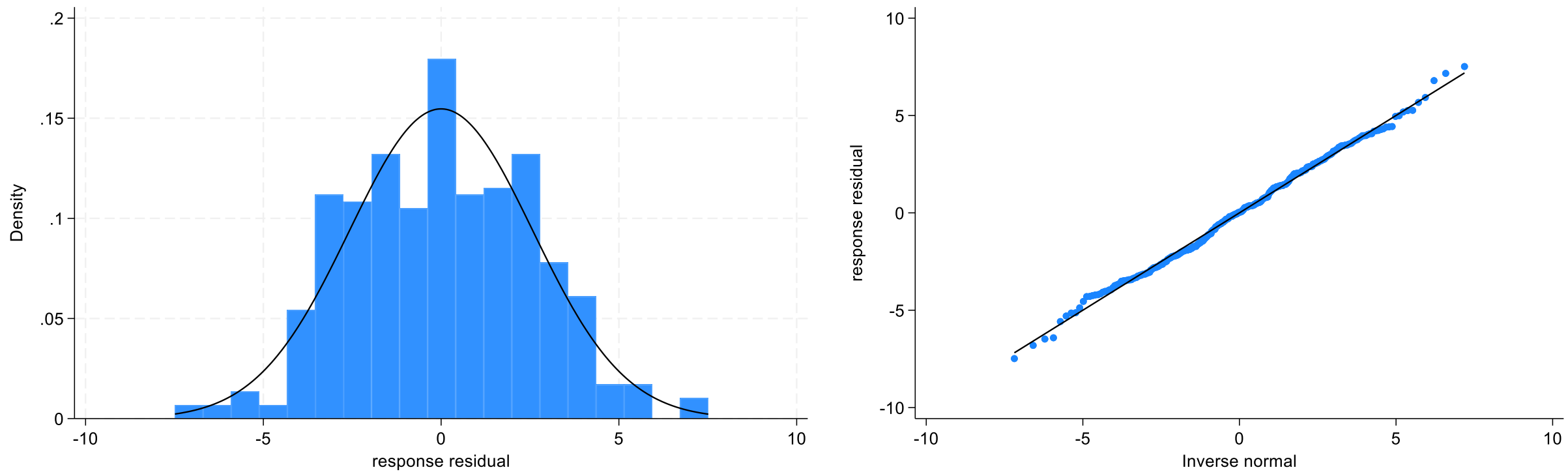

Figure 7.20: Graphical distribution of residuals from the adjusted regression models for EACQ Apprehension Concerning Negative Internal Experiences Related to Caregiving score at 24 weeks using histograms and normal probability plots.
Residual summary: Skewness = 0.04; Kurtosis = 2.74

Appendix 8: Reason for withdrawal from the study

Reason for withdrawal	iACT4CARERS (N=249)	TAU only (N=247)
Person with dementia deceased	3 (1.2%)	2 (0.8%)
Decline in the condition of the person with dementia	2 (0.8%)	1 (0.4%)
Decline in the physical health of the carer	2 (0.8%)	2 (0.8%)
Increased paid working hours	1 (0.4%)	0 (0%)
Increased caring responsibilities	0 (0%)	2 (0.8%)
Unable to engage with intervention	0 (0%)	0 (0%)
Other (including no reason given)	11 (4.4%)	10 (4.0%)

iACT4CARERS=Internet-delivered Self-help Acceptance and Commitment Therapy for Family Carer of People Living with Dementia. CSRI= Client Service Receipt Inventory. TAU=Treatment as Usual.

Appendix 9: Treatment completion data

Number and percentage of participants who attended various number of online sessions

Number of online sessions	Number of participants (N=249)	%
0	9	3.6
1	6	2.4
2	9	3.6
3	7	2.8
4	10	4.0
5	12	4.8
6	18	7.2
7	28	11.2
8	150	60.2

Mean (and SD), median, minimum, and maximum online sessions attended

**Mean (SD)**	6.62
**SD**	2.26
**Median**	8
**Minimum**	0
**Maximum**	8

Number and percentage of participants attending one or two one-to-one sessions

Number of face-to-face sessions	Number of participants (N=249)	%
0	75	30.1
1	94	37.8
2	80	32.1

Detailed reasons for withdrawal from the intervention

- Unable to interact with any of the audio exercises (n=1)
- Found the exercises difficult to engage with and felt uncomfortable during the exercises (n=1)
- Unable to continue due to health problems (n=1)
- The intervention materials did not feel relevant to the carer's situation (n=1)
- Did not have sufficient time to commit to the intervention (n=1)
- Did not enjoy the e-learning format (n=1)
- Felt pressured to complete the programme due to frequent text and email reminders (n=1)

Appendix 10: Outcome completeness

	Number completed at baseline (N= 496)	Number completed at 12 weeks post-randomisation	Number completed at 24 weeks post- randomisation
GAD-7	496 (100.0%)	398 (80.2%)	380 (76.6%)
PHQ-9	496 (100.0%)	398 (80.2%)	380 (76.6%)
CompACT	495 (99.8%)	395 (79.6%)	378 (76.2%)
EACQ	496 (100.0%)	393 (79.2%)	374 (75.4%)
STTS-R	··	200 (80.3%)	··
Expectations about treatment	496 (100.0%)	··	··
Treatment preference	496 (100.0%)	··	··
EQ-5D-5L	496 (100.0%)	395 (79.6%)	371 (74.8%)
ICECAP-O	496 (100.0%)	395 (79.6%)	373 (75.2%)
Modified CSRI	496 (100.0%)	392 (79.0%)	371 (74.8%)

Appendix 11: Extent and patterns of missing data

The summaries below refer to the primary and secondary outcomes specified for the clinical effectiveness analysis. At baseline, the extent of missing data was negligible. One participant was missing CompACT total and CompACT BA scores (0.2% each), while all other outcomes, demographics, and baseline covariates relevant for the clinical effectiveness analyses were fully observed. In particular, there was no missingness in the baseline covariates to be used in the analysis models including the minimisation factors (baseline GAD-7 score and ethnicity).

At 12-week follow-up, missingness increased consistently across outcomes, reflecting participant drop-out. For the primary outcome (GAD-7 at 12 weeks), data were unavailable for 98 of 496 participants (19.8%), with similar proportions in the intervention group (48/249, 19.3%) and the control group (50/247, 20.2%). The same pattern was observed for PHQ-9 (19.8%). Missingness was slightly higher for CompACT (20.4%), its subscales (20.4–20.8%), and the EACQ (20.8%).

At 24-week follow-up, the proportion of missing data increased further. For GAD-7, 116 participants (23.4%) had missing data (Intervention Arm: 25.3%; Control Arm: 21.5%). PHQ-9 had the same overall proportion (23.4%), while CompACT and its subscales ranged between 23.8% and 24.6%, and EACQ was missing for 24.6% overall (Intervention Arm: 23.1%; Control Arm: 26.1%). These figures suggest broadly similar levels of attrition between the two arms, though Intervention Arm had marginally higher missingness by 24 weeks.

The tabular inspection of the missing data patterns (table below) indicated that missingness was concentrated at the participant level (i.e. whole forms not returned at follow-up), consistent with withdrawal or loss to follow-up, rather than sporadic item-level missingness. Logistic regression models with a missingness indicator as the dependent variable showed that several observed covariates, including ethnicity, age, education level, current work status, referral route, extra-trial treatment and length of care, were predictive of missingness. This supports the plausibility of the Missing At Random (MAR) assumption, under which outcome missingness can be explained by observed data.

Missing data summary in primary and secondary clinical outcomes at different time points (Baseline, 12-week follow-up and 24-week follow-up)

Outcomes	*Total N*	*Overall missing n (%)*			*Intervention Arm (N=249) missing n (%)*			*Control Arm (N=247) missing n (%)*		
		Baseline	*12 weeks*	*24 weeks*	*Baseline*	*12 weeks*	*24 weeks*	*Baseline*	*12 weeks*	*24 weeks*
GAD7	496	0 (0.0)	98 (19.8)	116 (23.4)	0 (0.0)	48 (19.3)	63 (25.3)	0 (0.0)	50 (20.2)	53 (21.5)
PHQ9	496	0 (0.0)	98 (19.8)	116 (23.4)	0 (0.0)	48 (19.3)	63 (25.3)	0 (0.0)	50 (20.2)	53 (21.5)
CompACT	496	1 (0.2)	101 (20.4)	118 (23.8)	0 (0.0)	48 (19.3)	64 (25.7)	1 (0.4)	53 (21.5)	54 (21.9)
CompACT OE	496	0 (0.0)	101 (20.4)	118 (23.8)	0 (0.0)	48 (19.3)	64 (25.7)	0 (0.0)	53 (21.5)	54 (21.9)
CompACT BA	496	1 (0.2)	101 (20.4)	118 (23.8)	0 (0.0)	48 (19.3)	64 (25.7)	1 (0.4)	53 (21.5)	54 (21.9
CompACT VA	496	0	101	118	0	48	64	0	53	54

		(0.0)	(20.4)	(23.8)	(0.0)	(19.3)	(25.7)	(0.0)	(21.5)	(21.9)
EACQ	496	0 (0.0)	103 (20.8)	122 (24.6)	0 (0.0)	48 (19.3	57 (23.1)	0 (0.0)	55 (22.3)	65 (26.1)
EACQ AAB	496	0 (0.0)	103 (20.8)	122 (24.6)	0 (0.0)	48 (19.3	57 (23.1)	0 (0.0)	55 (22.3)	65 (26.1)
EACQ ACNIE	496	0 (0.0)	101 (20.4)	121 (24.4)	0 (0.0)	48 (19.3)	65 (26.1)	0 (0.0)	53 (21.5)	56 (22.7)
EACQ INTE	496	0 (0.0)	101 (20.4)	122 (24.6)	0 (0.0)	48 (19.3)	57 (23.1)	0 (0.0)	53 (21.5)	65 (26.1)

CompACT= Comprehensive Assessment of Acceptance and Commitment Therapy processes. CompACT OE=CompACT Openness to Experience. CompACT BA=CompACT Behavioural Awareness. CompACT VA=CompACT Valued Action. EACQ=Experiential Avoidance in Caregiving Questionnaire. EACQ AAB=EACQ Active Avoidant Behaviours. EACQ ACNIE=EACQ Apprehension Concerning Negative Internal Experiences Related to Caregiving. EACQ INTE=EACQ Intolerance of Negative Thoughts and Emotions Towards the Relative. GAD-7=Generalized Anxiety Disorder-7.

Appendix 12: Figures demonstrating mean outcome scores over time by group

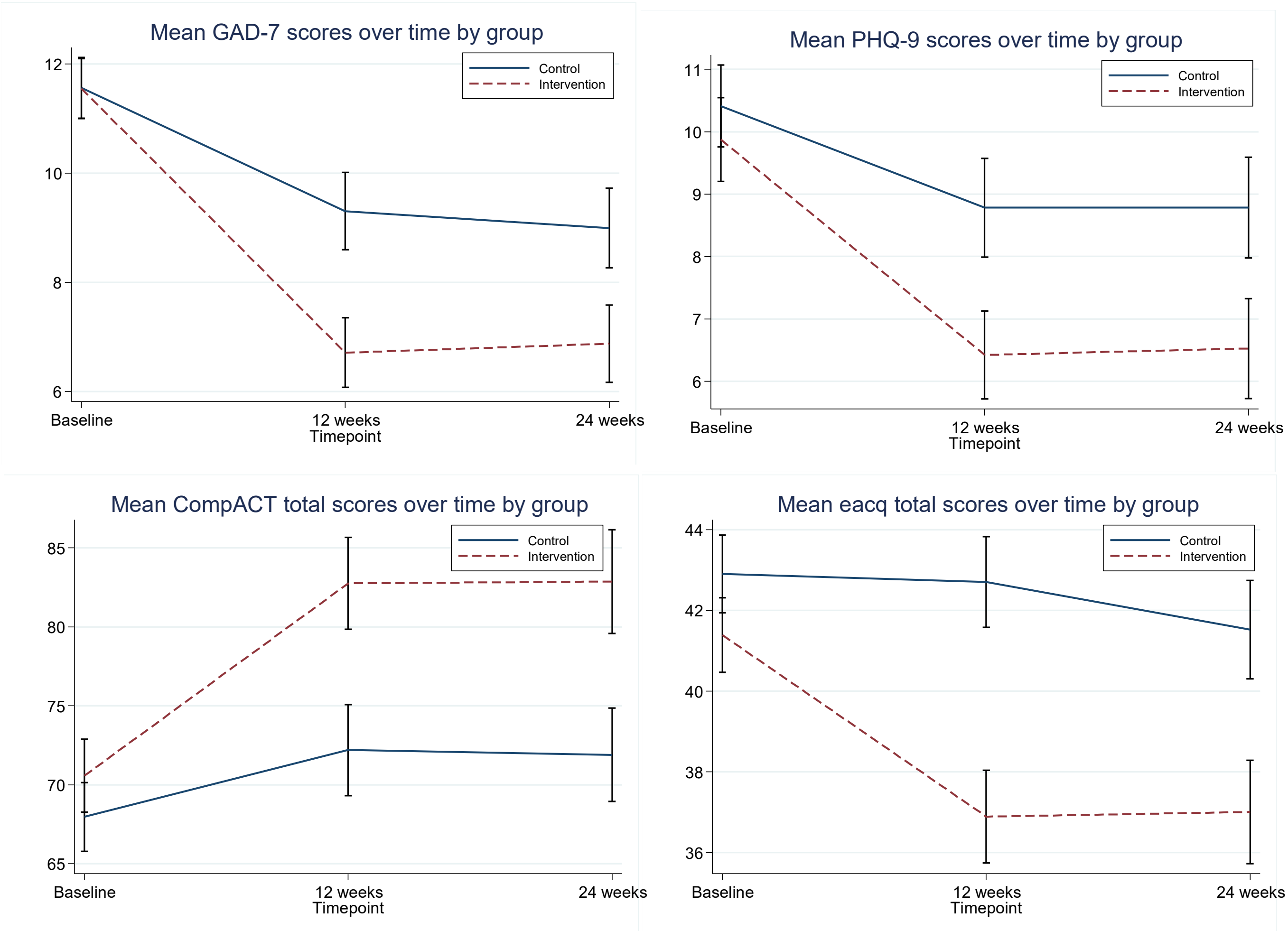

Figures demonstrating mean outcome scores over time by group (Intervention vs. Control), with 95% confidence intervals

Appendix 13: Safety data

Adverse events

	Intervention Arm Frequency (%) (N=249)		Control Arm Frequency (%) (N=247)	
	Trial related	Unrelated	Trial related	Unrelated
Clear evidence of high levels of distress making participants difficult to continue with the study	0	0	0	0
New reports of active suicidal ideation without active intent	0	2 (0.8%)	0	0
Stroke	0	1 (0.4%)	0	0

Serious adverse events

	Intervention Arm Frequency (%) (N=249)		Control Arm Frequency (%) (N=247)	
	Trial related	Unrelated	Trial related	Unrelated
New reports of active suicidal ideation with active intent	0	0	0	0
Death	0	0	0	0
Life threatening event	0	0	0	0
Event that requires hospitalisation or prolongs existing hospitalisation	0	0	0	0
Event that results in persistent or significant disability or incapacity	0	0	0	0
Other	0	0	0	0

Appendix 14: Sensitivity to missing data

Sensitivity analyses were conducted to assess the impact of missing outcome data using multiple imputation by chained equations. The imputation model included treatment allocation, baseline GAD-7, ethnicity, and auxiliary variables predictive of outcome or missingness. Logistic regression models with a missingness indicator as the dependent variable showed that several observed covariates, including ethnicity, age, education level, current work status, referral route, length of care and extra trial treatment at baseline, were predictive of missingness. This supports the plausibility of the Missing at Random (MAR) assumption, under which outcome missingness can be explained by observed data.

Given that approximately 20% to 26% of the outcome data were missing at 12- and 24-weeks outcomes, 30 imputed datasets were generated. This choice reflects current methodological guidance that the number of imputations should be at least as large as the percentage of incomplete cases.[1-2] Results from the multiple imputation sensitivity analysis were consistent with the primary ITT analysis.

Results of sensitivity analysis using multiple imputation for missing outcome data

Primary and secondary outcomes	Analysis of available data		Analysis of multiply imputed data	
	Adjusted mean difference (95% CI)	p value	Adjusted mean difference (95% CI)	p value
GAD7 Total score at 12 weeks	-2.62 (-3.47, -1.77)	<0.0001	-2.46 (-3.32, -1.59)	<0.00001
GAD7 Total score at 24 weeks	-2.11 (-3.01, -1.20)	<0.0001	-1.97 (-2.86, -1.07)	<0.0001
PHQ-9 total score at 12 weeks	-2.12 (-3.03, -1.21)	<0.0001	-2.00 (-2.89, -1.11)	<0.0001
PHQ-9 total score at 24 weeks	-1.94 (-2.91, -0.97)	<0.0001	-1.73 (-2.67, -0.79)	0.0001
CompACT total score at 12 weeks	8.56 (5.16, 11.97)	<0.0001	8.09 (4.76, 11.42)	<0.0001
CompACT total score at 24 weeks	9.71 (6.04, 13.38)	<0.0001	8.90 (5.03, 12.76)	<0.0001
CompACT Openness to Experience score at 12 weeks	4.47 (2.88, 6.06)	<0.0001	4.48 (2.93, 6.02)	<0.0001
CompACT Openness to Experience score at 24 weeks	4.64 (2.82, 6.47)	<0.0001	4.33 (2.55, 6.11)	<0.0001
CompACT Behavioural Awareness score at 12 weeks	2.25 (1.15, 3.36)	<0.0001	2.00 (0.92, 3.09)	<0.0001
CompACT Behavioural Awareness score at 24 weeks	2.76 (1.55, 3.97)	<0.0001	2.43 (1.25, 3.60)	<0.0001
CompACT Valued Action score at 12 weeks	2.06 (0.48, 3.65)	0.0107	1.80 (0.25, 3.35)	0.0230

CompACT Valued Action score at 24 weeks	2.49 (0.91, 4.08)	0.0021	2.17 (0.50, 3.83)	0.0106
EACQ total score at 12 weeks	-4.11 (-5.43, -2.78)	<0.0001	-4.14 (-5.41, -2.86)	<0.0001
EACQ total score at 24 weeks	-2.79 (-4.26, -1.32)	0.0002	-3.14 (-4.60, -1.68)	<0.0001
EACQ Active Avoidant Behaviours score at 12 weeks	-1.72 (-2.51, -0.93)	<0.0001	-1.69 (-2.51, -0.87)	<0.0001
EACQ Active Avoidant Behaviours score at 24 weeks	-0.98 (-1.81, -0.15)	0.0206	-1.04 (-1.88, -0.20)	0.0156
EACQ Intolerance score at 12 weeks	-1.82 (-2.38, -1.26)	<0.0001	-1.85 (-2.46, -1.24)	<0.001
EACQ Intolerance score at 24 weeks	-1.33 (-1.96, -0.70)	<0.0001	-1.43 (-2.08, -0.77)	<0.001
EACQ Apprehension score at 12 weeks	-0.75 (-1.31, -0.19)	0.0092	-0.78 (-1.33, -0.22)	0.0058
EACQ Apprehension score at 24 weeks	-0.81 (-1.35, -0.28)	0.0030	-0.90 (-1.44, -0.36)	0.0010

Appendix 15: Sensitivity to inadequate compliance

The compliance proportion in the intervention arm was just marginally below (79%) the threshold set (80%) for the planned sensitivity analysis for inadequate compliance. A CACE analysis was however performed as outlined in the Statistical Analysis Plan (SAP). The CACE estimation was implemented using the generalised structural equation modelling (gsem) procedure in Stata. Within the CACE analysis model, compliance indicator in the intervention arm was fixed at the observed level: 196/249 (78.7%) complied, 53/249 and (21.3%) not complied. Compliance within the control arm, which is a counterfactual, was estimated probabilistically using a latent class model within the gsem framework which included baseline GAD-7 score, ethnicity (white vs. non-white), indicator of receiving extra-trial treatments, treatment expectation score and treatment preference score as predictors. The outcome (GAD-7 at 12 weeks) within the CACE estimation (gsem) framework was specified as a linear model with a Gaussian family and identity link function which included the same covariates as for the primary analysis model.

The indicator of extra-trial treatment at baseline was defined based on receiving either medications for mental health or psychotherapy during the three months prior to randomisations summarised in Table 5.2.

The CACE estimate of the intervention effect (-2.86) was similar to that of the primary analysis (-2.62).

Results of CACE analysis for the primary outcome (GAD-7 at 12 weeks)

	**Estimate (SE)**	**95%CI**	**P value**
CACE estimate of intervention effect	-2.86 (0.57)	(-3.96, -1.75)	<0.0001

Appendix 16: Sensitivity to referral route

The referral route was coded as a binary indicator defined as: 1, if a participant was clinician referred, and 0, if self-referred.

The summary statistics of referral route indicator by the trial arms

**Referral routes**	**Intervention Arm (N=249)**	**Control Arm (N=247)**	**P value (Chi-Square)**
Self-referral (0)	101 (40.6%)	106 (42.9%)	0.5952
Clinician referrals (1)	148 (59.4%)	141 (57.1%)	

The distributions of self-referred and clinician referred participants were similar between the trial arms. Thus, the referral route is unlikely to be a confounder of the treatment effect. However, a sensitivity analysis was performed for the primary outcome (GAD7 total score at 12 weeks) by considering referral routes as one of the additional covariates. The intervention effect (adjusted mean difference) was estimated to be -2.62 with 95% confidence interval (-3.47, -1.77) and p-value <0.0001, which is almost identical to the intervention effect observed in the primary analysis.

The clinician referred participants had slightly lower GAD-7 score (regression Coeff = -0.24, 95% CI: -1.09, 0.62) compared the self-referred, but this difference was not statistically significant (p = 0.589).

Appendix 17: Sensitivity to treatment expectation/treatment preference

The distributions of treatment expectation and treatment preference are similar between the trial arms. Thus, the treatment expectation and treatment preference are unlikely to be a confounder of the treatment effect. However, a sensitivity analysis was performed for the primary outcome (GAD7 total score at 12 weeks) by considering referral routes as one of the additional covariates.

The intervention effect (adjusted mean difference) was estimated to be -2.56 with 95% confidence interval (-3.41, -1.70) and p-value <0.0001, which is almost identical to the intervention effect observed in the primary analysis.

	**Adjusted mean difference (95% CI) (multiple linear regression)**	**p value (multiple linear regression)**
GAD7 Total score at 12 weeks with		
Treatment Expectation	-2.55(-3.40, -1.69)	<0.0001
Treatment Preference	-2.62(-3.47, -1.77)	<0.0001
Treatment Expectation, Treatment Preference	-2.56(-3.41, -1.70)	<0.0001

Appendix 18: Mediation analysis of the effect of the intervention on anxiety symptoms via change in psychological flexibility

The mediation model

The simplified form of the regression-based mediation analysis undertaken is given below. The path **c** represents the total causal effect of X (Intervention) on the outcome Y (GAD-7 at 24 weeks). The path **a** represents the effect of X (Intervention) on the mediator M (CompACT change from baseline to 12 weeks). The path **b** represents the effect of mediator M (CompACT change from baseline to 12 weeks) on the outcome Y (GAD-7 at 24 weeks), adjusted for the effect of X (Intervention). The *total effect* (path **c**) can be partitioned into its *direct effect* (path $\boldsymbol{c'}$) and its *indirect effect* on the outcome Y (GAD-7 at 24 weeks) via the mediator M (CompACT change from baseline to 12 weeks).

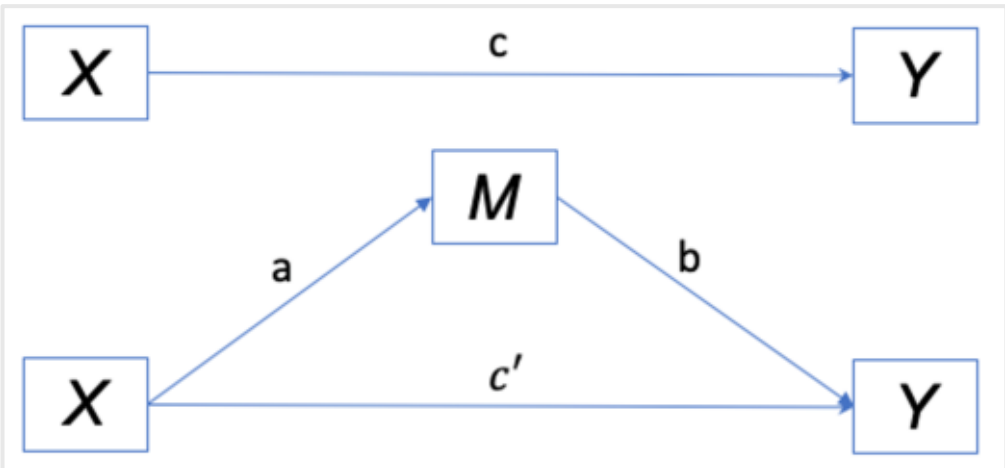

The indirect effect of X (Intervention) on the outcome Y (GAD-7 at 24 weeks) via the mediator M (CompACT change from baseline to 12 weeks) was quantified as the product of paths **a** and **b** (i.e., **a** × **b**). The total effect (**c**) was expressed as the sum of the direct ($\boldsymbol{c'}$) and indirect effects (**ab**): $\boldsymbol{c = c' + ab}$. Equivalently, the direct effects ($\boldsymbol{c'}$) was expressed as the difference between the total effect and the indirect effect: $\boldsymbol{c' = c - ab}$.

Mediation analysis of the effect of the intervention on GAD-7 at 24 weeks via change in CompACT (baseline to 12 weeks)

**Effect type**	**Estimate (Bootstrap SE)**	**95% Bootstrap CI**	**p-value**
Indirect effect (a×b)	-0.56 (0.176)	[-0.90, -0.21]	0.002
Direct effect (c')	-2.20 (0.445)	[-3.08, -1.33]	<0.0001
Total effect (c)	-2.76 (0.437)	[-3.62,1.33]	<0.0001
Proportion mediated (%)	20.2 (6.67)	[7.16, 33.32]	0.002

The intervention was associated with a 2.76-point reduction in GAD-7 overall (total effect, c = −2.76, bootstrap SE 0.437; 95% bootstrap CI −3.62 to −1.33; p<0.0001). The indirect effect via change in CompACT from baseline to 12 weeks was −0.56 points (bootstrap SE 0.176; 95% bootstrap CI −0.90 to −0.21; p=0.002), indicating that improvements in CompACT statistically mediated part of the intervention effect on 24-week GAD-7. The estimated proportion mediated was 20.2% (bootstrap SE 6.67; 95% bootstrap CI 7.16% to 33.32%; p=0.002), consistent with partial mediation, with approximately one-fifth of the total effect operating through CompACT change.

Appendix 19: Minimal Clinically Important Difference (MCID) analysis

Responder analysis for change from baseline to 12 weeks

Group	Non-Responder n (%), [95%CI]	Responder n (%), [95%CI]
Control (n=197)	95 (48.2%) [41.3, 55.2]	102 (51.8%) [44.8, 58.7]
Intervention (n=201)	46 (22.9%) [17.6, 29.2]	155 (77.1%) [70.8, 82.4]

Using the MCID definition as at least 20% improvement from baseline to 12 weeks, response rates differed markedly by group. In the control group, 102/197 participants were classified as responders (51.8%, 95% CI 44.8%–58.7%) and 95/197 as non-responders (48.2%, 95% CI 41.3%–55.2%). In contrast, in the intervention group 155/201 participants were responders (77.1%, 95% CI 70.8%–82.4%) and 46/201 were non-responders (22.9%, 95% CI 17.6%–29.2%). Overall, the intervention was associated with a substantially higher probability of achieving the MCID at 12 weeks than the control (absolute difference, 25.3 percentage points).

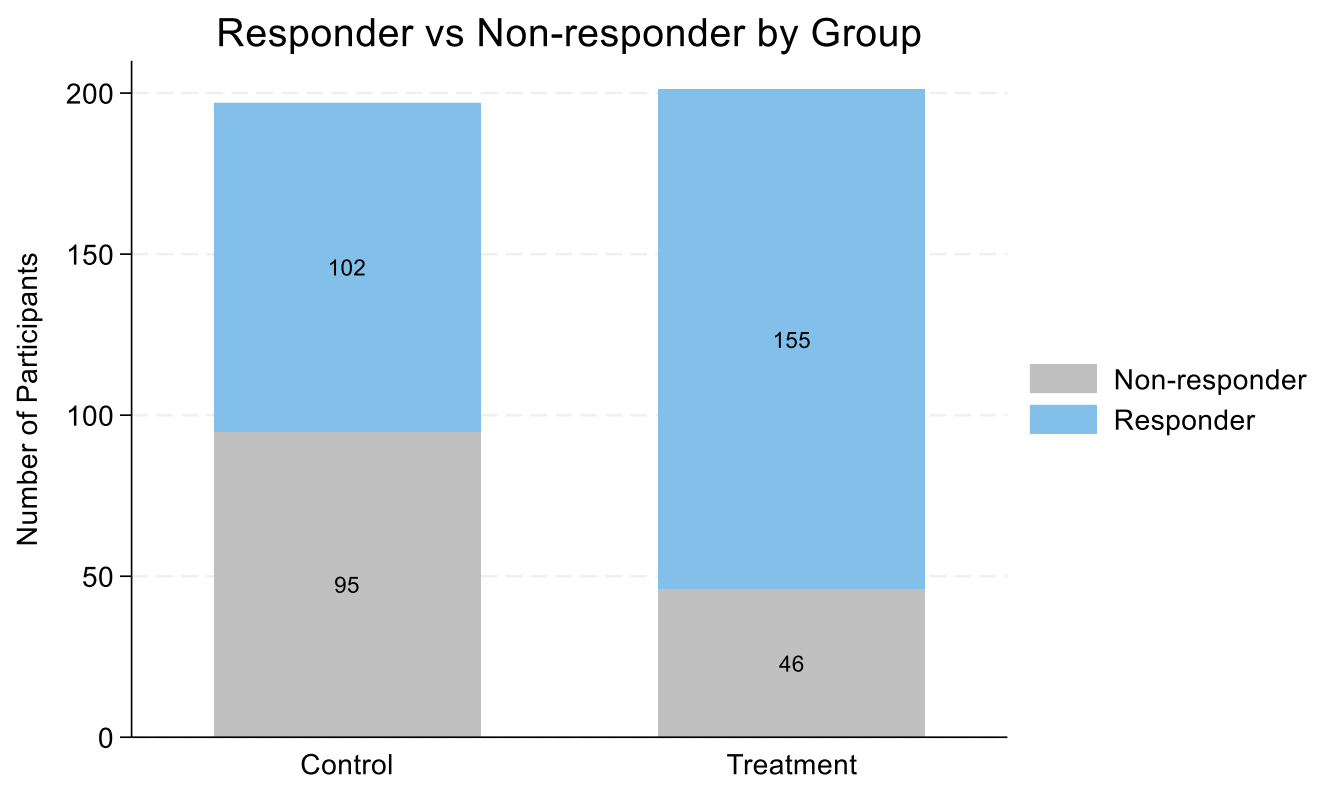

Responder analysis for change from baseline to 24 weeks

Group	Non-Responder n (%), [95%CI]	Responder n (%), [95%CI]
Control (n=194)	91 (46.9%) [40.0, 54.0]	103 (53.1%) [46.0, 60.0]
Intervention (n=186)	50 (26.9%) [21.0, 33.7]	136 (73.1%) [66.3, 79.0]

At 24 weeks, applying the MCID definition of at least 20% improvement from baseline, a larger proportion of participants in the intervention group achieved a clinically meaningful improvement in GAD-7 compared with the control group. Specifically, 136/186 (73.1%, 95% CI 66.3%–79.0%) in the intervention group met the responder definition, compared with 103/194 (53.1%, 95% CI 46.0%–60.0%) in the control group. Conversely, clinically non-meaningful change (non-response) was less common in the intervention group (26.9%, 95% CI 21.0%–33.7%) than in the control group (46.9%, 95% CI 40.0%–54.0%). Overall, these findings suggest that the intervention confers a sustained clinical benefit, increasing the likelihood of achieving a meaningful reduction in anxiety symptoms by approximately 20 percentage points at 24 weeks.

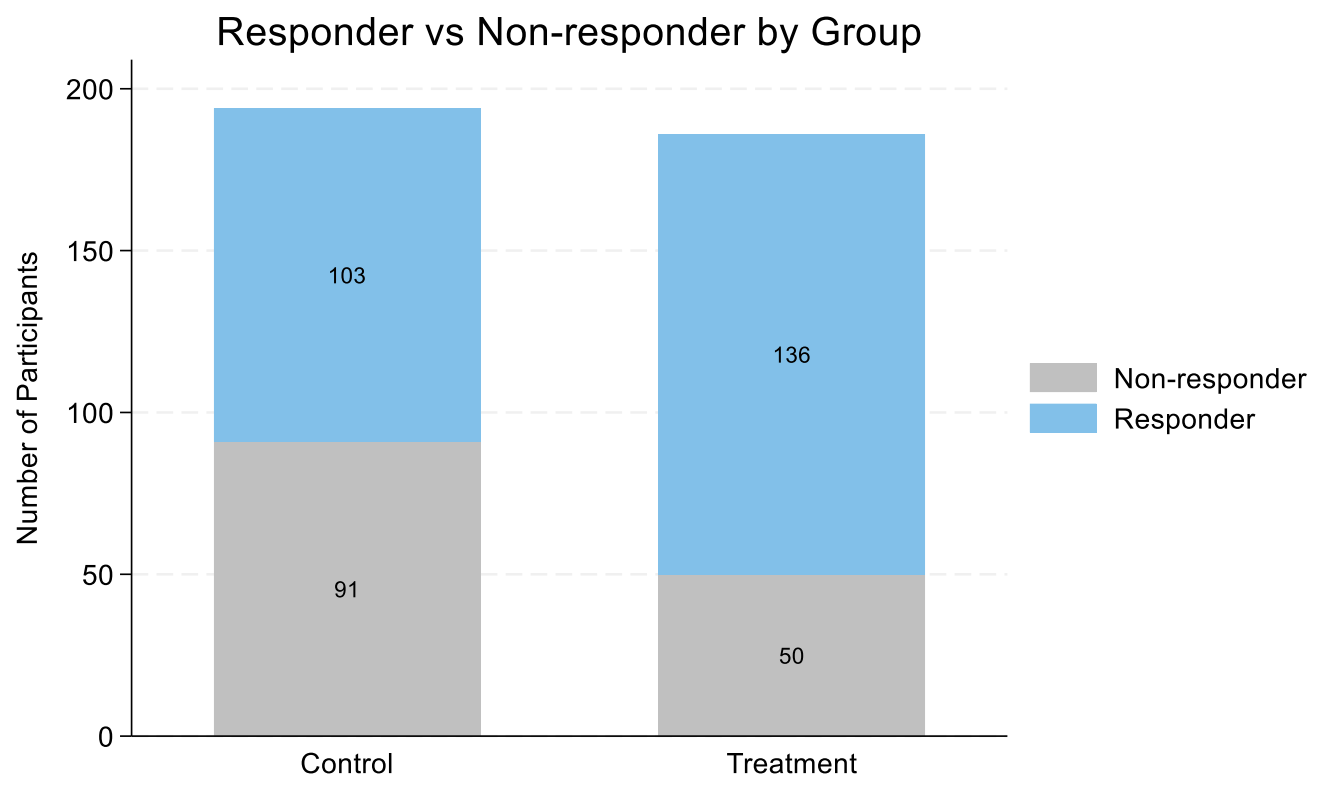

Appendix 20: Analysis of participant shifts across GAD-7 severity categories

Intervention group versus control comparison at 12 weeks

Group	GAD-7 at Baseline	GAD-7 at 12 weeks				
		**Minimal (0–4)**	**Mild (5–9)**	**Moderate (10–14)**	**Severe (15–21)**	**Total**
**Intervention**	**Mild (5–9)**	42 (51.9%)	31 (38.3%)	3 (3.7%)	5 (6.2%)	81 (100.0%)
	**Moderate (10–14)**	24 (34.3%)	34 (48.6%)	10 (14.3%)	2 (2.9%	70 (100.0%)
	**Severe (15–21)**	8 (36.0%)	17 (34.0%)	17 (34.0%)	8 (16.0%)	50 (100.0%)
	**Total**	74	82	30	15	201
**Control**	**Mild (5–9)**	27 (34.6%)	35 (44.9%)	31 (16.7%)	3 (3.9%)	78(100.0%)
	**Moderate (10–14)**	6 (8.5%)	32 (45.1%)	19 (26.7%)	14 (19.7%	71(100.0%)
	**Severe (15–21)**	2 (4.17%)	14 (29.2%)	12 (25%)	20 (41.7%)	48(100.0%)
	**Total**	35	81	44	37	197

At 12 weeks, the severity distribution was materially more favourable in the intervention group than in the control group. Minimal severity (0-4) was observed in 74/201 participants (36.8%) in the intervention group versus 35/197 (17.8%) in the control group (absolute difference, +19.0 percentage points), while severe symptoms (15-21) occurred in 15/201 (7.5%) versus 37/197 (18.8%), respectively (absolute difference, -11.3 percentage points). Considering higher-severity categories together, the proportion with moderate or severe symptoms at 12 weeks was 45/201 (22.4%) in the intervention group compared with 81/197 (41.1%) in the control group (absolute difference, -18.7 percentage points). Transition (row) percentages further supported these findings. Among participants with mild symptoms at baseline (5-9), improvement to minimal severity occurred in 42/81 (51.9%) in the intervention group versus 27/78 (34.6%) in the control group. Among those with moderate symptoms at baseline (10-14), improvement to minimal or mild severity occurred in 58/70 (82.9%) in the intervention group compared with 38/71 (53.5%) in the control group, while worsening to severe symptoms was less frequent (2 of 70 [2.9%] vs 14 of 71 [19.7%]). Among participants with severe symptoms at baseline (15-21), remaining in the severe category at 12 weeks was less common in the intervention group (8 of 50 [16.0%]) than in the control group (20 of 48 [41.7%]), while improvement to minimal or mild severity was more frequent (25 of 50 [50.0%] vs 16 of 48 [33.3%]).

Intervention group versus control comparison at 24 weeks

Group	GAD-7 at Baseline	GAD-7 at 24 weeks				
		**Minimal (0–4)**	**Mild (5–9)**	**Moderate (10–14)**	**Severe (15–21)**	**Total**
**Intervention**	**Mild (5–9)**	40 (52.0%)	27 (35.1%)	5 (6.5%)	5 (6.5%)	77 (100.0%)
	**Moderate (10–14)**	20 (31.8%)	31 (49.2%)	9 (14.3%)	3 (4.8%)	63 (100.0%)
	**Severe (15–21)**	5 (10.9%)	19 (41.3%)	12 (26.1%)	10 (21.7%)	46 (100.0%)
	**Total**	65	77	26	18	186
**Control**	**Mild (5–9)**	28 (35.4%)	34 (43.0%)	13 (16.5%)	4 (5.1%)	79 (100.0%)
	**Moderate (10–14)**	9 (12.7%)	27 (38.0%)	26 (36.6%)	9 (12.7%	71 (100.0%)
	**Severe (15–21)**	1 (2.3%)	12 (27.3%)	12 (27.3%)	19 (43.2%)	44 (100.0%)
	**Total**	38	73	51	32	194

At 24 weeks, the GAD-7 severity distribution remains shifted in a more favourable direction in the intervention group than in the control group. The proportion with minimal symptoms was 65/186 (35.0%) in the intervention group versus 38/ 194 (19.6%) in the control group, while severe symptoms were observed in 18/ 186 (9.7%) versus 32/194 (16.5%), respectively. Consequently, the combined proportion with moderate or severe symptoms was 44/186 (23.7%) in the intervention group compared with 83/194 (42.8%) in the control group (absolute difference, −19.1 percentage points). Among participants with severe symptoms at baseline (15-21), remaining in the severe category at 24 weeks was less common in the intervention group (10 of 46 [21.7%]) than in the control group (19 of 44 [43.2%]), while improvement to minimal or mild severity was more frequent (24 of 46 [52.2%] vs 13 of 44 [29.5%]).

Appendix 21: Content of each session of iACT4CARERS

Session number	Main focus of the session
1	Learning what Acceptance and Commitment Therapy is. Learning to let go of the need to control painful emotions. Learning to embrace and allow painful emotions.
2	Leaning to clarify values (what is truly important in life) and identify committed actions (actions that reflect chosen values).
3	Learning to overcome external barriers (e.g., limited free time) using a three-step problem-solving approach.
4	Learning to overcome internal barriers (e.g., distressing thoughts) using cognitive fusion techniques (stepping back from internal struggles).
5	Learning to overcome internal barriers (e.g., worries about future or past) using present moment awareness techniques (focusing on the present).
6	Learning to treat oneself with kindness using self-compassion techniques.
7	Learning to break down an action into small steps to build a pattern of effective action that reflects values.
8	Revisiting all skills learned and preparing for the future.

Standard ACT exercises and metaphors were used, with all case examples reflecting common experiences of family carers of people living with dementia. Carers often face distinctive external barriers (e.g., physical demands and reduced free time) and internal barriers (e.g., caregiving-related beliefs and guilt shaped by cultural and societal expectations, including familism), which can limit engagement in value-based activities beyond caregiving. To enhance relevance and effectiveness in this population, iACT4CARERS incorporated selected standard exercises and metaphors deemed suitable for this group, including the values-based "Garden of Your Life" exercise, alongside self-regulation strategies from the Selective Optimisation with Compensation framework[3] to support flexible identification of values and engagement in valued-based activities across caregiving and personal domains. The intervention also integrated self-compassion exercises within ACT to help carers manage internal barriers and foster a more adaptive caregiving perspective. Session-by-session objectives and ACT exercises and metaphors are detailed in our feasibility trial appendix. [4]

The ACT adaptation was initially discussed with four public members (family carers with anxiety symptoms) and subsequently with six carers from underrepresented ethnic minority groups in the UK to ensure relevance and acceptability. The structure and content of the guided internet-delivered self-help ACT intervention were developed through consensus among ACT experts in the research team, and a written protocol was produced. An administrative staff member with no knowledge of any form of psychotherapy including ACT also reviewed the protocol to ensure the terms and descriptions used were clear for the public. The prototype of the online programme was piloted by two public members (family carers) to ensure its relevance and the user-friendliness of the online platform before the feasibility study.

Appendix 22: Treatment expectations and intervention preference questionnaires

1. How much do you expect your symptoms to improve if you receive internet-delivered Acceptance and Commitment Therapy?	☐ 0 (Not at all)<br>☐ 1<br>☐ 2<br>☐ 3<br>☐ 4 (completely)
2. How much do you expect your life to improve if you receive internet-delivered Acceptance and Commitment Therapy?	☐ 0 (Not at all)<br>☐ 1<br>☐ 2<br>☐ 3<br>☐ 4 (completely)
3. Although you will be chosen at random to have either internet-delivered Acceptance and Commitment Therapy or usual care alone, if you could choose what treatment you received, how much would you hope to receive internet-delivered Acceptance and Commitment Therapy plus usual care?	☐ 0 (Not at all)<br>☐ 1<br>☐ 2<br>☐ 3<br>☐ 4 (completely)
4. How much would you hope to receive usual care alone (i.e. no internet-delivered Acceptance and Commitment Therapy)?	☐ 0 (Not at all)<br>☐ 1<br>☐ 2<br>☐ 3<br>☐ 4 (completely)

Appendix 23: PRECIS-2 assessment of trial design

PRECIS-2 domain	Score	Justification
**Eligibility**	5 (Very pragmatic)	Eligibility criteria were broad and closely aligned with usual care. Participants were highly representative of those who would be eligible to receive the intervention in routine clinical practice.
**Recruitment**	1 (Very explanatory)	Recruitment was conducted outside usual care pathways to maximise community reach, including targeted outreach to community groups (e.g., ethnic minority groups). Recruitment relied on trial-specific procedures rather than routine care processes.
**Setting**	3 (Equally pragmatic and explanatory)	The intervention was delivered in local secondary care settings (usual care context). However, the online platform was centrally hosted, managed, and monitored by the trial team, introducing trial-specific infrastructure beyond routine care.
**Organisation**	3 (Equally pragmatic and explanatory)	Therapists received additional trial-specific training beyond routine practice. Technical support and participant assistance were provided centrally by the trial team rather than local services, reflecting partial centralisation of care organisation.
**Flexibility (delivery)**	2 (Rather explanatory)	Intervention delivery was protocol-driven with limited flexibility. Therapists were required to provide feedback within seven days, and adherence to delivery protocols was centrally monitored.
**Flexibility (adherence)**	3 (Equally pragmatic and explanatory)	Participant engagement was monitored, and non-engagement for several weeks triggered follow-up for safety reasons. These procedures were managed centrally rather than through local clinical services.
**Follow-up**	2 (Rather explanatory)	Follow-up involved scheduled data collection at two time points via direct participant contact, exceeding the level of follow-up typically expected in routine care.
**Primary outcome**	5 (Very pragmatic)	The primary outcome (anxiety) was directly relevant to participants and routine clinical practice, reflecting outcomes typically used in usual care decision-making.
**Primary analysis**	5 (Very pragmatic)	An intention-to-treat analysis was used, including all randomised participants regardless of adherence, consistent with pragmatic trial principles.

References for Appendices

1. Coope B, Ballard C, Saad K, et al. The prevalence of depression in the carers of dementia sufferers. *International Journal of Geriatric Psychiatry* 1995; **10**(3): 237-42
2. White IR, Royston P, Wood AM. Multiple imputation using chained equations: Issues and guidance for practice. *Statistics in Medicine* 2011; **30**(4): 377-99.
3. Baltes PB, Baltes MM. Psychological perspectives on successful aging: The model of selective optimization with compensation. In: Baltes PB, Baltes MM, eds. Successful aging: Perspectives from the behavioral sciences. New York, NY: Cambridge University Press; 1990: 1-34.
4. Kishita N, Gould RL, Farquhar M, et al. Internet-delivered guided self-help Acceptance and Commitment Therapy for family carers of people with dementia (iACT4CARERS): A feasibility study. *Aging and Mental Health* 2022; **26**(10): 1933-41.
