# Supplementary material for: Clinical effectiveness of internet-delivered self-help Aacceptance and Commitment Therapy for family carers of people with dementia (iACT4CARERS): a multicentre, parallel, randomised controlled trial
Source: Lancet Reg Health Eur. 2026 Jul 11;67:101766. doi: 10.1016/j.lanepe.2026.101766 (PMC13453953; doi:10.1016/j.lanepe.2026.101766)
Supplement: Supplementary file 2 [file mmc2.pdf]

iACT4CARERS

The clinical and cost effectiveness of internet-delivered self-help Acceptance and Commitment Therapy for family carers of people with dementia (iACT4CARERS): A randomised controlled trial with ethnically diverse family carers

Version	1.0
Date	21 December 2022
Sponsor	University of East Anglia
Trial registration	ISRCTN45995725
REC IRAS #	324157

Authorisation: Chief Investigator

Name	Associate Professor Naoko Kishita
Role	Chief Investigator
Signature	木下奈緒子
Date	21 December 2022

Authorisation: CTU Director or Representative

Name	Mr Matthew Hammond
Role	Norwich Clinical Trials Unit Deputy Director
Signature	
Date	03.01.2023

Authorisation: Trial Statistician

Name	Associate Professor Mizanur Khondoker
Role	Lead Statistician
Signature	
Date	03 January 2023

Table of Contents

1 Administrative information 1

1.1 Compliance 1

1.2 Sponsor 1

1.3 Structured trial summary 2

1.4 Roles and responsibilities 5

1.4.1 Protocol contributors 5

1.4.2 Role of trial sponsor and funders 5

1.4.3 Trial Team 6

1.4.4 Trial Management Group 6

1.4.5 Trial Steering Committee 7

1.4.6 Data Monitoring and Ethics Committee 8

2 Trial Diagram 9

3 Abbreviations 10

4 Glossary 10

5 Introduction 11

5.1 Background and Rationale 11

5.1.1 Explanation for choice of comparators 13

5.2 Objectives 13

5.3 Trial Design 13

5.3.1 Progression criteria 14

6 Methods 15

6.1 Site Selection 15

6.1.1 Study Setting 15

6.1.2 Site/Investigator Eligibility Criteria 15

6.1.2.1 Principal Investigator's (PI) Qualifications and Agreements 16

6.1.2.2 Resourcing at site 16

6.2 Site approval and activation 16

6.3 Participants 16

6.3.1 Eligibility Criteria 16

6.3.1.1 Participant selection 16

6.3.1.2 Participant Inclusion Criteria 17

6.3.1.3 Participant Exclusion Criteria 17

6.3.1.4 Eligibility Criteria for Individuals Performing the Interventions 18

6.3.1.5 Co-enrolment Guidance 18
6.3.1.6 Screening Procedures and Pre-randomisation Investigations 18
6.4 Interventions 18
6.4.1 Arm A (Intervention) 18
6.4.1.1 iACT4CARERS plus TAU 18
6.4.1.2 Treatment Schedule 19
6.4.2 Arm B (Control) 19
6.4.2.1 Treatment-as-Usual (TAU) 19
6.4.2.2 Treatment Schedule 19
6.4.3 Compliance and Adherence 19
6.4.4 Concomitant Care 20
6.4.5 Protocol Treatment Discontinuation 20
6.5 Outcomes 20
6.5.1 Primary Outcomes 20
6.5.2 Secondary Outcomes 21
6.5.3 Qualitative Study/Process Evaluation 21
6.6 Participant Timeline 23
6.6.1 Participant Assessments 24
6.6.1.1 Screening and Baseline 24
6.6.1.2 Intervention 24
6.6.1.2 12-week and 24-week Follow-Ups 24
6.6.2 Early Stopping of Follow-up 25
6.6.3 Participant Transfers 25
6.6.4 Loss to Follow-up 25
6.6.5 Trial Closure 26
6.7 Sample Size 26
6.8 Recruitment and Retention 26
6.8.1 Recruitment 26
6.8.2 Retention 27
6.9 Assignment of Intervention 28
6.9.1 Allocation 28
6.9.1.1 Sequence generation 28
6.9.1.2 Allocation concealment mechanism 28
6.9.1.3 Allocation Implementation 28
6.9.2 Blinding 28

6.9.3 Emergency Unblinding ... 28
6.10 Data Collection, Management and Analysis ... 28
6.10.1 Data Collection Methods ... 28
6.10.2 Data Management ... 29
6.10.3 Non-Adherence and Non-Retention ... 30
6.10.4 Statistical Methods ... 30
6.10.4.1 Outcomes ... 30
6.10.4.2 Statistical Analysis Plan ... 30
6.10.4.3 Additional Analyses ... 30
6.10.4.4 Analysis Population ... 30
6.10.4.5 Missing Data ... 30
6.10.5 Economic evaluations ... 31
6.10.5.1 Health Economic Analysis Plan ... 32
6.10.6 Analysis of Qualitative Information ... 32
6.11 Data Monitoring ... 32
6.11.1 Data Monitoring and Ethics Committee ... 32
6.11.2 Interim Analyses ... 32
6.11.3 Data Monitoring for Harm ... 32
6.11.3.1 Safety reporting ... 32
6.11.3.2 Other Notifiable Adverse Events ... 33
6.11.3.5 Investigator responsibilities relating to safety reporting ... 33
6.11.3.5.1 Seriousness assessment ... 33
6.11.3.5.2 Severity or grading of Adverse Events ... 33
6.11.3.5.3 Causality ... 34
6.11.3.5.4 Expectedness ... 34
6.11.3.6 Notifications ... 35
6.11.3.6.1 Notifications by the Investigator to NCTU ... 35
6.11.3.6.2 NCTU responsibilities ... 35
6.11.4 Quality Assurance and Control ... 35
6.11.4.1 Risk Assessment ... 35
6.11.4.2 Central Monitoring at NCTU ... 36
6.11.4.3 On-site Monitoring ... 36
6.11.4.3.1 Direct access to participant records ... 36
6.11.4.4 Trial Oversight ... 36
6.11.4.4.1 Trial Team ... 36

6.11.4.4.2 Trial Management Group.....36
6.11.4.4.3 Independent Trial Steering Committee.....36
6.11.4.4.4 Independent Data Monitoring and Ethics Committee.....37
6.11.4.4.5 Trial Sponsor.....37
7 Ethics and Dissemination.....38
7.1 Research Ethics and Health Research Authority Approval.....38
7.2 Other Approvals.....38
7.4 Amendments.....38
7.5 Consent or Assent.....38
7.5.1 Consent or Assent in Ancillary Studies.....39
7.6 Confidentiality.....39
7.7 Declaration of Interests.....39
7.8 Indemnity.....39
7.9 Finance.....39
7.10 Archiving.....40
7.11 Access to Data.....40
7.12 Ancillary and Post-trial Care.....40
7.13 Publication Policy.....40
7.13.1 Trial Results.....40
7.13.2 Authorship.....40
7.13.3 Reproducible Research.....40
8 Ancillary Studies.....41
9 Protocol Amendments.....42
10 References.....43
11 Appendices.....46
12 Principal Investigator compliance statement.....47

1 Administrative information

This document was constructed using the Norwich Clinical Trials Unit (NCTU) Protocol template Version 4. It describes the iACT4CARERS trial, sponsored by the University of East Anglia and co-ordinated by NCTU.

It provides information about procedures for entering participants into the trial, and provides sufficient detail to enable: an understanding of the background, rationale, objectives, trial population, intervention, methods, statistical analyses, ethical considerations, dissemination plans and administration of the trial; replication of key aspects of trial methods and conduct; and appraisal of the trial's scientific and ethical rigour from the time of ethics approval through to dissemination of the results. The protocol should not be used as an aide-memoire or guide for the treatment of other patients. Every care has been taken in drafting this protocol, but corrections or amendments may be necessary. These will be circulated to registered investigators in the trial. Sites entering participants for the first time should confirm they have the correct version through a member of the trial team at NCTU.

NCTU supports the commitment that its trials adhere to the SPIRIT guidelines. As such, the protocol template is based on the Standard Protocol Items: Recommendations for Interventional Trials (SPIRIT) 2012 Statement for protocols of clinical trials[1]. The SPIRIT Statement Explanation and Elaboration document[2] can be referred to, or a member of NCTU Protocol Review Committee can be contacted for further detail about specific items.

1.1 Compliance

The trial will be conducted in compliance with the approved protocol, the Declaration of Helsinki (2008), the principles of Good Clinical Practice (GCP) as laid down by the Commission Directive 2005/28/EC with implementation in national legislation in the UK by Statutory Instrument 2004/1031 and subsequent amendments, , the UK Data Protection Act, and the UK Policy Framework for Health and Social Care Research, the Mental Capacity Act 2005, and other national and local applicable regulations. Agreements that include detailed roles and responsibilities will be in place between participating sites and NCTU.

Participating sites will inform NCTU as soon as they are aware of a possible serious breach of compliance, so that NCTU can fulfil its requirement to report the breach if necessary within the timelines specified in the UK Clinical Trials Regulations (currently 7 days). For the purposes of this regulation a 'serious breach' is one that is likely to affect to a significant degree:

- The safety or physical or mental integrity of the subjects in the trial, or
- The scientific value of the trial.

1.2 Sponsor

The University of East Anglia is the trial sponsor and has delegated responsibility for the overall management of the iACT4CARERS trial to the Chief Investigator and NCTU. Queries relating to sponsorship of this trial should be addressed to the Chief Investigator or via the trial team.

1.3 Structured trial summary

Primary Registry and Trial Identifying Number	ISRCTN Registry ID: ISRCTN45995725
Date of Registration in Primary Registry	12/04/2023
Secondary Identifying Numbers	IRAS ID: 324157<br>Sponsor reference: R210595
Source of Monetary or Material Support	National Institute for Health and Care Research Health Technology Assessment (HTA) Programme: NIHR150071
Sponsor	University of East Anglia
Contact for Public Queries	iact4carers.study@uea.ac.uk
Contact for Scientific Queries	Dr Naoko Kishita<br>Associate Professor in Dementia and Complexity in Later Life<br>School of Health Sciences<br>University of East Anglia<br>Norwich Research Park<br>Norwich, NR4 7TJ<br>Email: N.Kishita@uea.ac.uk
Short Title or Acronym	A clinical and cost effectiveness randomised controlled trial of iACT4CARERS
Scientific Title	The clinical and cost effectiveness of internet-delivered self-help Acceptance and Commitment Therapy for family carers of people with dementia (iACT4CARERS): A randomised controlled trial with ethnically diverse family carers
Countries of Recruitment	England
Health Condition(s) or Problem(s) Studied	Family carers of people with dementia presenting with anxiety symptoms
Intervention(s)	**Intervention arm: iACT4CARERS plus treatment-as-usual (TAU)**<br>Internet-delivered self-help Acceptance and Commitment Therapy (ACT) for family carers of people with dementia (iACT4CARERS) consists of eight online sessions. Each session has three phases: self-learning, reflection and practice. The self-learning phase will guide participants through core ACT skills (OPEN, AWARE and ENGAGED) that together constitute psychological flexibility. The reflection phase encourages participants to reflect on learned skills and leave questions

	for their trial therapist if anything is unclear. Trial therapists will provide individually tailored written feedback to validate and normalise difficult thoughts and emotions participants are experiencing and encourage them to practice ACT skills they found helpful. The practice phase allows participants to set a goal and practice ACT skills offline between online sessions.<br><br>In addition to online sessions, two 30-min one-to-one sessions via telephone or video call will be offered to each participant by their trial therapist. These one-to-one sessions are optional and not signing up for these will not result in the withdrawal from iACT4CARERS. iAC4CARERS will be delivered in addition to TAU as described below.<br><br>**Control arm: TAU alone**<br>The control is treatment as usual (TAU) alone. Participants in either group will continue to receive any routine support, such as Admiral Nurse appointments, during the trial. They will not be discouraged from seeking treatment outside of the study for ethical reasons. As per NICE guideline [NG97], TAU for carers normally consists of information, brief education, practical advice, local carer support groups and/or respite care provided by health and social care services, or relevant charities based on the needs.
Key Inclusion and Exclusion Criteria	**Inclusion criteria**<br>1) Aged 18 years and over.<br>2) Caring for a family member diagnosed with dementia.<br>3) Presenting with anxiety symptoms as assessed by the General Anxiety Disorder-7 (GAD7).<br>4) Help-seeking (seeking help with their anxiety through iACT4CARERS).<br>5) Having access to internet through Wi-Fi or mobile data.<br><br>**Exclusion criteria**<br>1) Lacking capacity to provide fully informed written consent.<br>2) Currently receiving ongoing formal psychological therapy.<br>3) Experiencing disabling medical or mental health problems making participation inappropriate or impractical.<br>4) Expressing active suicidal intent.
Study Type	This study will be a multicentre, single-blind, parallel, 2-arm randomised controlled trial comparing iACT4CARERS plus

	TAU with TAU alone. Randomisation will be generated by a secure web-based system on a 1:1 basis with minimisation for anxiety severity range and ethnicity.
Date of First Enrolment	November 2023
Target Sample Size	496 participants (248 per arm)
Primary Outcome(s)	Anxiety symptoms will be evaluated using the General Anxiety Disorder-7 (GAD7) at baseline and 12- and 24-week post randomisation.
Key Secondary Outcomes	**Clinical outcomes**<br>• Depressive symptoms: Patient Health Questionnaire-9 (PHQ9)<br>• Psychological flexibility: Comprehensive Assessment of Acceptance and Commitment Therapy processes (CompACT)<br>• Experiential avoidance in caregiving: Experiential Avoidance in Caregiving Questionnaire (EACQ)<br><br>**Cost-effectiveness outcomes**<br>• Health-related quality of life: EQ-5D-5L<br>• Capability in older people: ICEpop CAPability measure for Older people (ICECAP-O)<br>• Health and social care service utilisation: Modified Client Service Receipt Inventory (modified CSRI)<br><br>**Treatment satisfaction and intervention fidelity measures will be collected only at 12-week post randomisation:**<br>• Satisfaction With Therapy and Therapist Scale-Revised (STTS-R)<br>• Intervention fidelity: ACT Fidelity Measure (ACT-FM)<br>• Therapist response time (the time gap between the participant leaving a comment and their therapist providing a response)

1.4 Roles and responsibilities

These membership lists are correct at the time of writing; please see terms of reference documentation in the Trial Master File (TMF) for current lists.

1.4.1 Protocol contributors

Name	Affiliation	Role
Dr Naoko Kishita	University of East Anglia	Chief Investigator
Professor Rebecca Gould	University College London	Co-Investigator
Professor Lance McCracken	Uppsala University	Co-Investigator
Professor Morag Farquhar	University of East Anglia	Co-Investigator
Dr Mizanur Khondoker	University of East Anglia	Co-Investigator, Lead Statistician
Mr David Turner	University of East Anglia	Co-Investigator, Lead Health Economist
Mr Matthew Hammond	University of East Anglia	Co-Investigator, Norwich CTU Deputy Director
Dr Erica Richmond	Norfolk and Suffolk NHS Foundation Trust	Co-Investigator
Dr Aditya Nautiyal	The Clarkson Surgery	Co-Investigator
Ms Barbara Czyznikowska	Centre for Ethnic Health Research	Co-Investigator, PPI Lead

1.4.2 Role of trial sponsor and funders

Name	Affiliation	Role
Ms Danelle Breach	University of East Anglia	Sponsor, Project Officer
Mr Alan Marshall	NIHR HTA	Funder, Research Manager

1.4.3 Trial Team

Name	Affiliation	Role and responsibilities
Dr Naoko Kishita	University of East Anglia	Chief Investigator
Dr Mizanur Khondoker	University of East Anglia	Co-Investigator, Lead Statistician
Dr Polly-Anna Ashford	University of East Anglia	CTU Research Lead
TBC	University of East Anglia	CTU Clinical Trial Manager
TBC	University of East Anglia	Research Associate
TBC	University of East Anglia	Research Associate

1.4.4 Trial Management Group

Name	Affiliation	Role and responsibilities
Dr Naoko Kishita	University of East Anglia	Chief Investigator
Professor Rebecca Gould	University College London	Co-Investigator
Professor Lance McCracken	Uppsala University	Co-Investigator
Professor Morag Farquhar	University of East Anglia	Co-Investigator
Dr Mizanur Khondoker	University of East Anglia	Co-Investigator, Lead Statistician
Mr David Turner	University of East Anglia	Co-Investigator, Lead Health Economist
Mr Matthew Hammond	University of East Anglia	Co-Investigator, Norwich CTU Deputy Director

Dr Erica Richmond	Norfolk and Suffolk NHS Foundation Trust	Co-Investigator
Dr Aditya Nautiyal	The Clarkson Surgery	Co-Investigator
Ms Barbara Czyznikowska	Centre for Ethnic Health Research	Co-Investigator, PPI Lead
Ms Ruby Ali-Strayton	PPI member	PPI Co-Investigator
Dr Polly-Anna Ashford	University of East Anglia	CTU Research Lead
TBC	University of East Anglia	CTU Clinical Trial Manager
TBC	University of East Anglia	Research Associate
TBC	University of East Anglia	Research Associate

1.4.5 Trial Steering Committee

Name	Affiliation	Role and responsibilities
Dr David Gillanders	University of Edinburgh	Chair
Professor Daniel Stahl	King's College London	Independent member, Statistician
Dr Anju Devianee Keetharuth	University of Sheffield	Independent member, Health economist
Dr Christopher Graham	University of Belfast	Independent member
Mr Peter Davis	NA	Independent PPI member
Mr Geoff Angel	NA	Independent PPI member
Dr Naoko Kishita	University of East Anglia	Non-independent member, Chief Investigator

Dr Mizanur Khondoker	University of East Anglia	Non-independent member, Co-Investigator, Lead Statistician

1.4.6 Data Monitoring and Ethics Committee

Name	Affiliation	Role and responsibilities
Dr Sam Norton	King's College London	Chair
Dr Matthew Bursnall	University of Sheffield	Independent member, Statistician
Professor Rosa Romero Moreno	University of Rey Juan Carlos	Independent member

2 Trial Diagram

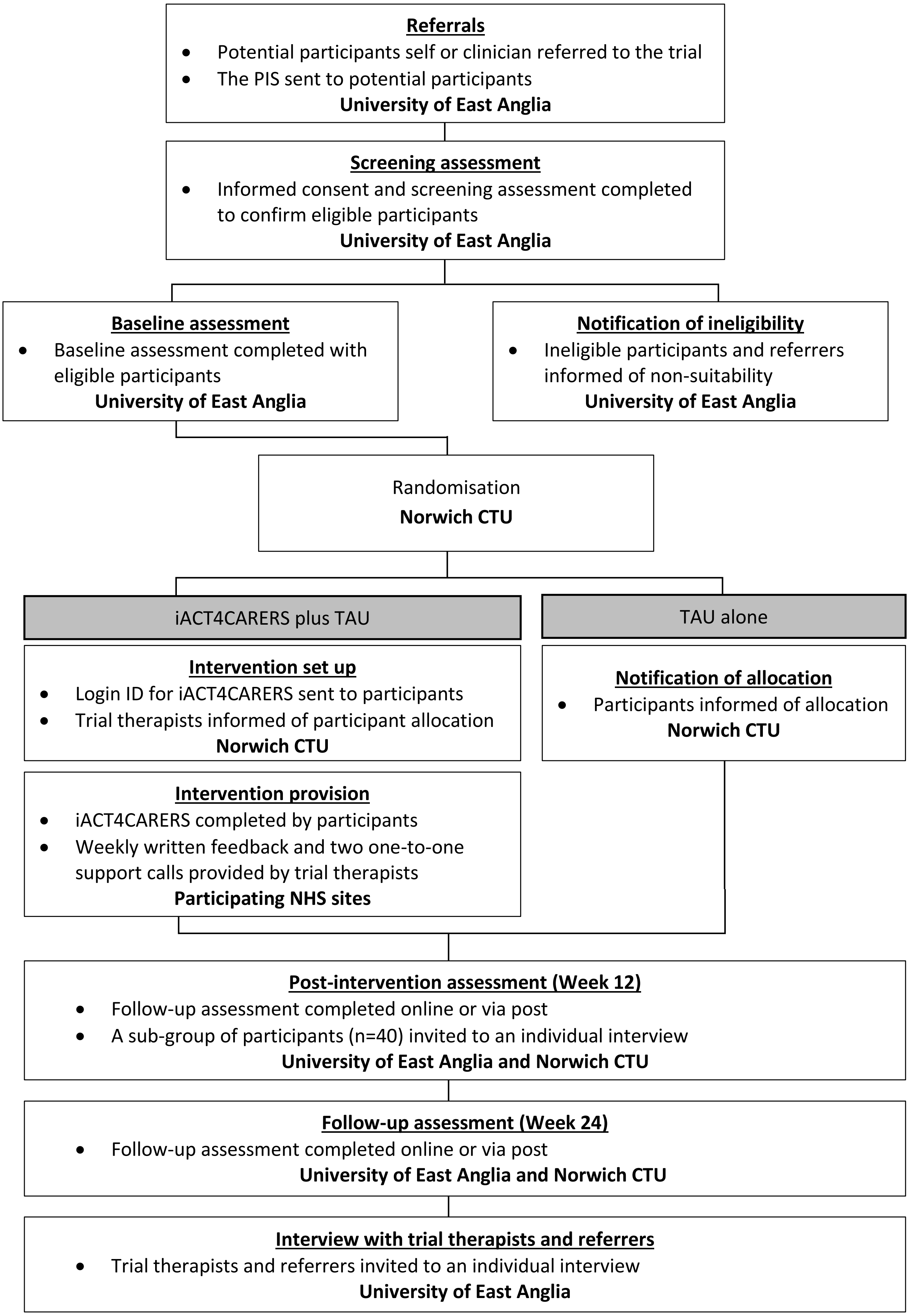

3 Abbreviations

ACT	Acceptance and Commitment Therapy
ACT-FM	Acceptance and Commitment Therapy Fidelity Measure
AE	Adverse Event
CRF	Case Report Form
CompACT	Comprehensive Assessment of Acceptance and Commitment Therapy Processes
CSRI	Client Service Receipt Inventory
DMEC	Data Monitoring and Ethics Committee
EACQ	Experiential Avoidance in Caregiving Questionnaire
EU	European Union
GAD7	General Anxiety Disorder-7
GCP	Good Clinical Practice
HEAP	Health Economics Analysis Plan
HRA	Health Research Authority
ICECAP-O	Icepop Capability Measure for Older People
ICH	International Conference on Harmonisation
ISF	Investigator Site File
ITT	Intention to Treat
NCTU	Norwich Clinical Trials Unit
PIC	Participant Identification Centre
PHQ9	Patient Health Questionnaire-9
PID	Participant Identification Number
PIS	Participant Information Sheet
PPI	Patient and Public Involvement
QA	Quality Assurance
QC	Quality Control
QMMP	Quality Management and Monitoring Plan
R&D	Research and Development
RCT	Randomised Controlled Trial
REC	Research Ethics Committee
SAE	Serious Adverse Event
SAP	Statistical Analysis Plan
SSA	Site Specific Approval
STTS-R	Satisfaction With Therapy and Therapist Scale-Revised
TAU	Treatment-as-Usual
TMF	Trial Master File
TMT	Trial Management Team
ToR	Terms of Reference
TSC	Trial Steering Committee
UEA	University of East Anglia

4 Glossary

None

5 Introduction

5.1 Background and Rationale

What is the problem?

Previous systematic reviews demonstrated that the prevalence of anxiety and depression in family carers of people with dementia is around 32%[3, 4]. These estimates are substantially higher than reported prevalence rates in the general population in the UK[5] indicating a clear need for support with their mental health. The current standard care pathway for mental health conditions in family carers of people with dementia includes self or GP referral to NHS psychological services[6]. However, many family carers are not able to access timely psychological support due to various barriers such as mobility issues, lack of respite care and a shortage of skilled therapists leading to long waiting lists[7]. One way to address the clear need for improved access, and also scalability, is to design a service that can be delivered remotely, accessed independently at home, and at times chosen by the carer. Online treatments can do this.

Psychological treatments for family carers of people with dementia, which have been previously tested such as the STrAtegies for RelaTives (START) intervention[8], are mainly delivered face-to-face and are based on conventional cognitive behaviour therapy (CBT). A recent review of in-person CBT for family carers of people with dementia demonstrated a small to medium overall effect on depression (d=0.34)[9]. A meta-analysis focusing solely on technology-based self-help CBT for family carers of people with dementia demonstrated a small overall effect on depression (d=0.27)[10]. No significant overall effect was observed for anxiety in either review, suggesting that conventional CBT may have a limited effect on carer anxiety. These results suggest a need for improvement in psychological therapies among this population. We particularly need to identify an intervention that can (1) be delivered in a self-help format so that it is accessible by all for the reasons noted above and (2) effectively target anxiety to overcome challenges observed in previous studies.

Why ACT?

Acceptance and Commitment Therapy (ACT) is a form of psychotherapy with a strong evidence base for improving outcomes such as mood and quality of life in various populations, including people with anxiety, depression, chronic pain and somatic health problems[11-13]. ACT does not aim to change thoughts and feelings but rather to reduce avoidance and enhance personally meaningful behaviour. ACT achieves this by promoting psychological flexibility through three sets of skills, including the ability to (1) “step back” from restricting thoughts and allow painful emotions (i.e. OPEN); (2) focus on the present, connected with what is going on around us in the moment (i.e. AWARE); and (3) clarify and act on what is most important to do and build increasing patterns of effective values-based actions (i.e. ENGAGED)[14].

This strategic focus is highly practical because it is easier to change what we do, and how we do it, than to stop or change what we think[14]. As such, it has been argued that ACT is particularly useful for those living with immutable and chronic conditions[15]. Since many family carers also face uncontrollable circumstances, ACT may be well suited to this population. Indeed, a recent systematic review on carer interventions demonstrated strong empirical support for face-to-face ACT in treating anxiety and depression in family carers of people with dementia[16].

Recent systematic reviews also suggest that ACT skills can be learnt online[17-19]. In the UK, feasibility studies of manualised ACT for people with complex chronic pain converted to an online

mode of delivery[20, 21] led to numerous enhancements of the treatment; the upgraded treatment was approved and implemented in the NHS just four years after the research began.

iACT4CARERS feasibility study

We received funding from the NIHR Research for Patient Benefit Programme (PB-PG-0418-20001) to (1) develop internet-delivered self-help ACT for family carers of people with dementia (iACT4CARERS), (2) explore whether iACT4CARERS is acceptable in this population and (3) test whether it is feasible to deliver iACT4CARERS within NHS services. iACT4CARERS was offered on a self-learning basis, with minimal contact with a non-expert, minimally trained therapist.

Pre-defined criteria for progression from the feasibility study to a full-scale trial required iACT4CARERS to be deemed acceptable to family carers as indicated by uptake and initial engagement (recruitment of 30 eligible carers over 6 months, with ≥70% completing at least two online sessions). These criteria were successfully met. Participant recruitment took place from August 2020 and January 2021 during the COVID-19 pandemic with 108 carers referred across three sites over six months. Thirty-three eligible family carers were recruited, with ≥91% completing more than two sessions, suggesting excellent acceptability[22]. There was preliminary evidence of improvements in anxiety, depression and psychological flexibility, particularly in anxiety, which demonstrated an average reduction of 26% on the GAD7 anxiety scale (a reduction of 20% on GAD7 is considered to be a Minimum Clinically Important Difference[23]). Therefore, randomised controlled trial (RCT) evidence on clinical- and cost-effectiveness of this new intervention is warranted.

NHS and social care need

Currently, family carers of people with dementia who receive the least support are those from ethnic minority groups. A recent systematic review of studies that included UK South Asian patients with dementia could not identify a single clinical trial of an intervention in this population, either for patients or carers[24]. An online approach such as iACT4CARERS has the potential to improve the psychological well-being of carers, cost effectiveness to NHS services, and importantly equity of access to care. Thus, a full-scale RCT to evaluate the effectiveness of iACT4CARERS in a diverse carer population is warranted.

This project is also timely and ethically justified from the perspective of the NHS and social care need. The NHS Long Term Plan (2019) sets the development of Integrated Care Systems to increasingly focus on population health as one of the top priorities[25]. This policy emphasises the need for preventative services, which individuals can access at a time and place that is convenient for them[26]. iACT4CARERS does not require highly trained expert therapists, and our feasibility study was successfully delivered within GP and NHS psychological services entirely remotely during the pandemic. This flexibility in delivery can address practical challenges currently faced by the NHS.

ACT with people from minority groups

A previous review, which explored cultural competence in ACT, suggested that ACT has been implemented across many countries including low-/middle-income countries such as India and South Africa[27]. A recent study, which demonstrated the effectiveness of guided self-help ACT among South Sudanese refugees in a large RCT (n=694)[28], led to the development of non-guided self-help ACT for coping with adversity during the pandemic, and is available on the website of the World Health Organisation in 11 different languages. A recent large trial (N=2,415), which involved 868

ethnic minority participants, also demonstrated that smartphone-delivered ACT was effective for smoking cessation[29]. These suggest that there is a strong potential for iACT4CARERS to be used with a diverse group of carers including those from ethnic minority groups.

5.1.1 Explanation for choice of comparators

The treatment-as-usual (TAU) condition will be used to evaluate if iACT4CARERS offers an improvement over current practice. Participants in either group will continue to receive any routine support, such as Admiral Nurse appointments, during the trial. They will not be discouraged from seeking treatment outside of the study for ethical reasons. TAU will be monitored, and we will undertake additional exploratory data analysis to assess the impact of contamination if a substantial proportion of participants have used pharmacological or psychological therapies (further described in the data analysis section).

5.2 Objectives

This study aims to undertake a definitive RCT with an internal pilot phase to determine the clinical and cost effectiveness of iACT4CARERS plus TAU compared to TAU alone in family carers of people with dementia who present with anxiety symptoms.

Primary hypothesis

- iACT4CARERS plus TAU will be superior to TAU alone in improving anxiety symptoms in family carers of people with dementia at 12 weeks post-randomisation.

Secondary hypotheses

- iACT4CARERS plus TAU will be superior to TAU alone in improving anxiety symptoms over a 24-week follow-up period.
- iACT4CARERS plus TAU will be superior to TAU alone in improving other mental health outcomes (e.g. psychological flexibility, depression) over 12-week and 24-week follow-up periods.
- iACT4CARERS plus TAU will be superior to TAU alone in terms of cost-effectiveness (cost per QALY).

This study also aims to assess fidelity and quality of implementation, establish causal mechanisms and identify contextual factors associated with variation in outcomes using the process evaluation approach.

Research questions for the process evaluation

- How is the delivery of iACT4CARERS achieved (fidelity and dose)?
- Are there any adaptations required to deliver iACT4CARERS as intended (adaptations)?
- To what extent can iACT4CARERS reach its intended service users (reach)?
- What are the mediating factors and context that may influence implementation and outcomes (mechanisms and context)?

5.3 Trial Design

This is a multi-site, single-blind, parallel, 2-arm RCT with ascertainment of clinical and cost-effectiveness of iACT4CARERS plus TAU delivered over a 12-week period compared to TAU alone on family carers of people with dementia presenting anxiety symptoms. Primary and secondary outcomes will be evaluated at 12 weeks and 24 weeks post randomisation.

5.3.1 Progression criteria

Progression criteria for the commencement of the trial

The red/amber/green system will be used to assess whether the trial can be delivered as planned and is feasible for the budget set.

Green: If the trial commences within two months of the planned start date (01 December 2023), we will continue the internal pilot as planned.

Amber: If the trial commences within three months of the planned start date, we will develop a mitigation plan to deliver the study as originally proposed. The plan will be reviewed by the Trial Steering Committee (TSC) and the NIHR Health Technology Assessment (HTA) team. They will determine whether it is considered feasible and acceptable to the HTA and whether the trial should continue with amendments.

Red: If the trial does not commence within three months after the planned start date, we will consider with the TSC and the NIHR HTA whether the trial should be terminated.

Progression criteria for the internal pilot

The RCT will contain a 10-month internal pilot to assess the feasibility of referral rates and acceptability of randomisation. At the end of the 10-month internal pilot phase, progress will be reviewed based on the pre-determined criteria (see Table 1) with the TSC. Green light will indicate support for the progression as planned. In the case of amber light, we will develop a mitigation plan to deliver the study as originally proposed. The plan will be reviewed by the TSC and the NIHR HTA team. They will determine whether it is considered feasible and acceptable to the NIHR HTA and whether the trial should continue with amendments. In the case of red light, we will consider with the TSC and the NIHR HTA whether the trial should be terminated.

We will also assess treatment adherence as part of the internal pilot. Based on our feasibility study[22], participants completing six or more online sessions will be considered treatment completers. We will evaluate treatment adherence data for those who have reached the end of the 12-week intervention phase during the internal pilot. As per Table 1, more than 70% of participants need to be identified as treatment completers to achieve Green (50-69% of participants will indicate Amber).

Table 1. Red/amber/green progression criteria

Internal pilot phase: 10 months

Progression criteria	Red	Amber	Green
% against the final recruitment target	<25%	25-49%	50%
% against the recruitment target for pilot	<50%	50-99%	100%
Recruitment rate/site/month	<1.2	1.20-1.69	1.7
Number of sites opened	<7	7-14	15
Total number of participants recruited	<124	124-247	248
Treatment adherence (% of completers)	<50%	50-69%	70%

6 Methods

6.1 Site Selection

The trial sponsor has overall responsibility for site and investigator selection and has delegated this role to the Chief Investigator and NCTU.

6.1.1 Study Setting

Potential participants will be identified through primary and secondary care services, including GP practices, Community Mental Health Teams and memory clinics. In addition, various methods (used successfully in our feasibility study) will be used in the community to reach potential participants. This includes advertisement through local newspapers (the most successful strategy in our feasibility study), Join Dementia Research and local community services, including dementia cafés.

Culturally acceptable recruitment strategies recommended by our PPI members will also be used to maximise recruitment of participants from ethnic minority groups. These include visiting places of worship, contacting local faith leaders and circulating the information through social media and WhatsApp befriending groups, which have become popular during the COVID-19 pandemic. Setting up these approaches will be supported by our collaborating partner, the Centre for Ethnic Health Research.

Ten NHS Participant Identification Centres (PICs), which are mainly likely to be GP practices, will be set up for only identifying potential participants. No further research activity will take place at PICs. In addition to 10 PICs, a maximum of 20 trial sites (36 therapists) will be identified and the requirements for these trial sites are described below.

6.1.2 Site/Investigator Eligibility Criteria

Once a site has been assessed as being suitable to participate in the trial, the trial team will provide them with a copy of this protocol.

To participate in the iACT4CARERS trial, investigators and trial sites must fulfil a set of criteria that have been agreed upon by the iACT4CARERS Trial Management Group (TMG) and that are defined below.

Eligibility criteria:

- A named clinician is willing and appropriate to take Principal Investigator responsibility;
- Healthcare staff having access to the target population are available to recruit participants (any healthcare staff will be eligible);
- Trial therapists meeting eligibility criteria are available to provide written feedback for online sessions completed by each participant and two supportive telephone or video calls to each participant.

Research Associates based at UEA supported by NCTU will be responsible for screening eligibility, obtaining consent and conducting research assessments. Thus, trial sites will not be required to undertake these activities.

Trial sites meeting eligibility criteria will be issued with the iACT4CARERS Investigator Site File (ISF) and a pack of documentation needed by the Research and Development Department (R&D) of their Trust to enable the Trust to provide confirmation of capacity and capability to undertake the study.

6.1.2.1 Principal Investigator's (PI) Qualifications and Agreements

The investigator(s) must be willing to sign an investigator statement to comply with the trial protocol (confirming their specific roles and responsibilities relating to the trial, and that their site is willing and able to comply with the requirements of the trial). This includes confirmation of appropriate qualifications and agreement to comply with the principles of GCP, to permit monitoring and audit as necessary at the site and to maintain documented evidence of all staff at the site who have been delegated significant trial-related duties.

6.1.2.2 Resourcing at site

The investigator(s) should be able to demonstrate a potential for recruiting the required number of suitable subjects within the agreed recruitment period (i.e. the investigator(s) regularly treat(s) the target population). They should also have an adequate number of qualified staff and facilities available for the foreseen duration of the trial to enable them to conduct the trial properly and safely. Sites will be expected to complete a delegation of responsibilities log and provide staff contact details.

6.2 Site approval and activation

On receipt of the signed investigator statement, approved delegation of responsibilities log and staff contact details, written confirmation will be sent to the site Principal Investigator. The Trial Manager or delegate will notify the Principal Investigator in writing of the plans for site initiation. The Principal Investigator is required to complete Good Clinical Practice training if they do not hold a current certificate. Sites will not be permitted to recruit any patients until a letter for activation has been issued. The Trial Manager or delegate will be responsible for issuing this after a green light to recruit process has been completed.

The site must conduct the trial in compliance with the protocol as agreed by the Sponsor and HRA, and which was given favourable opinion by the Research Ethics Committee (REC). The Principal Investigator or delegate must document and explain any deviation from the approved protocol and communicate this to the trial team at NCTU.

6.3 Participants

6.3.1 Eligibility Criteria

6.3.1.1 Participant selection

There will be **NO EXCEPTIONS** (waivers) to eligibility requirements at the time of randomisation. Questions about eligibility criteria should be addressed PRIOR to attempting to randomise the participant.

The eligibility criteria for this trial have been carefully considered and are the standards used to ensure that only clinically appropriate participants are entered. Participants not meeting the criteria should not be entered into the trial for their safety and to ensure that the trial results can be appropriately used to make future treatment decisions for other people with similar conditions. It is therefore vital that exceptions are not made to these eligibility criteria.

Participants will be considered eligible for enrolment in this trial if they fulfil all the inclusion criteria and none of the exclusion criteria as defined below.

6.3.1.2 Participant Inclusion Criteria

1) **Aged 18 years and over:** The content of iACT4CARERS is written for adults as the majority of family carers are in their 50s to 70s in the UK.
2) **Caring for a family member diagnosed with dementia:** Any family members including in-laws, such as daughters-in-law, will be eligible. The diagnosis of dementia will be based on the clinical information provided by the participant (carer). Participants will be asked to confirm whether the care recipient has received a formal diagnosis of dementia from a relevant healthcare professional. Participants will also be asked to confirm whether they provide regular care (self-defined) to the care recipient. No criteria will be set for frequency of care they provide, such as hours of caring per week, as the psychological impact of caring is related to multiple factors such as the relationship they have with the person with dementia. For example, a son caring for his mother living with dementia who can only visit a few hours per week due to work could still be feeling that he is providing regular care and experiencing a high level of anxiety. We will collect background and demographic data such as frequency of care they provide and their relationship to the care recipient.
3) **Presenting anxiety symptoms:** Participants will be asked to complete a measure of anxiety (General Anxiety Disorder-7: GAD7[30]) during the screening session. Those scoring in the clinical range of five or above will be eligible. Those scoring in the non-clinical range will be excluded to avoid a floor effect on the level of anxiety at baseline.
4) **Help-seeking:** A brief description of the intervention will be included in the information sheet. Participants will be asked if they would like to receive help with their anxiety through iACT4CARERS. Only those who want to receive help will be eligible.
5) **Having access to internet:** Participants will need to have access to a computer, tablet, or smartphone connected to the internet. iACT4CARERS is accessible from any platform on any device, except for those devices that use an old operating system no longer supported (e.g. Windows 7) as we cannot ensure the security of data. iACT4CARERS automatically blocks access from these unsupported devices.

6.3.1.3 Participant Exclusion Criteria

1) **Lacking capacity to provide fully informed written consent:** The capacity to consent to participate in the study will be assessed and recorded using a Mental Capacity ACT (2005) assessment tool developed by Norfolk and Suffolk NHS Foundation Trust. Participants will not be included in the study if they are unable to provide this.
2) **Currently receiving ongoing formal psychological therapy:** Participants will be asked to report any ongoing treatment they are receiving from healthcare services during the screening session. Participants currently receiving formal psychological therapy such as CBT, psychodynamic psychotherapy, systemic therapy and counselling will be excluded.
3) **Experiencing disabling medical or mental health problems making participation inappropriate or impractical:** Participants will be asked to report any ongoing untreated condition during the screening session. Following the baseline assessment, the participant’s GP will be informed of their involvement in the study, and any concerns raised by their GP regarding their involvement will be followed by a full discussion with the participant.
4) **Expressing active suicidal intent:** The risk of suicide will be assessed during the screening session. This will be assessed using the suicide risk assessment protocol co-developed with Norfolk and Suffolk NHS Foundation Trust. This protocol has been used across multiple studies run by our team. Those presenting suicidal ideation with active intent will be excluded.

Potential participants with insufficient understanding of English to complete screening measures and engage in the intervention will be encouraged to bring a family member or a friend who can support the participant and act as an interpreter. This approach was recommended by PPI members from ethnic minority groups. If this option is not available, a professional interpreter can be arranged through the third company, which UEA already has a contract in place. If the study team needs to directly contact another family member or an interpreter regarding the participant such as arranging the appointment, verbal consent will be obtained from the participant for doing so.

6.3.1.4 Eligibility Criteria for Individuals Performing the Interventions

Trial therapists will be mainly Band 4 NHS staff such as assistant practitioners, assistant psychologists, OT assistants, OT support workers and nursing associates. If Band 4 NHS staff are not available at the participating trial site, trial therapists can be Band 3 NHS staff such as social care support workers, Band 5 NHS staff such as social prescribers and psychological wellbeing practitioners or Band 6 NHS staff. Clinicians who hold a formal qualification in clinical psychology or CBT, such as Clinical Psychologists, CBT therapists and counselling therapists, will not be eligible.

Trial therapists will be required to attend a 2-day training workshop online, which has been tested in our previous feasibility study, prior to the start of the trial. Fortnightly drop-in supervision sessions led by clinical psychologists trained in ACT will be available via video call throughout the trial.

6.3.1.5 Co-enrolment Guidance

Those who participated in the Phase 1 study of the iACT4CARERS trial will not be eligible to participate in this study. Participants will not be permitted to enrol in this trial if they are currently enrolled in any other clinical trials of psychotherapy.

6.3.1.6 Screening Procedures and Pre-randomisation Investigations

Written informed consent to enter and be randomised into the trial must be obtained from participants after explanation of the aims, methods, benefits and potential hazards of the trial and **BEFORE** any trial-specific procedures. The only procedures that may be performed in advance of written informed consent being obtained are those that would be performed on all patients in the same situation as usual standard of care.

Following written informed consent being obtained, all participants will be asked to complete a screening assessment. The screening assessment includes:

- Eligibility checklist (a series of statements asking that they meet eligibility criteria);
- Demographic questionnaire;
- General Anxiety Disorder-7 (GAD7[30]).

Those who meet eligibility criteria will be asked to complete all baseline measures.

6.4 Interventions

6.4.1 Arm A (Intervention)

6.4.1.1 iACT4CARERS plus TAU

The iACT4CARERS intervention (https://iact4carers.com), which has been tested in our previous feasibility study will be used. iACT4CARERS will be delivered in addition to TAU as described in ARM B. iACT4CARERS consists of eight online sessions. Each session has three phases: self-learning,

reflection and practice. The self-learning phase will guide participants through core ACT skills (OPEN, AWARE and ENGAGED) that together constitute psychological flexibility. Interactive exercises to illustrate ACT skills will be presented using multiple modes (video/audio/text). The reflection phase encourages participants to reflect on learned skills and leave questions for their trial therapist if anything is unclear. Trial therapists will provide individually tailored written feedback to validate and normalise difficult thoughts and emotions participants are experiencing and encourage them to practice ACT skills they found helpful. The practice phase allows participants to set a goal and practice ACT skills offline between online sessions.

In addition to online sessions, two 30-min one-to-one sessions via telephone or video call will be offered to each participant by their trial therapist. These one-to-one sessions can be booked anytime while completing iACT4CARERS, but participants will be encouraged to book the first session at the start of the programme and the second session a few weeks after this. These sessions will focus on encouraging participants to: (1) express their feelings and emotional needs; (2) share their challenges and concerns regarding the use of technology; and (3) discuss the expectation for weekly reflection and online feedback from their trial therapist so that support can be tailored. These one-to-one sessions are optional and not signing up for these will not result in the withdrawal from iACT4CARERS.

6.4.1.2 Treatment Schedule

Participants will be asked to complete eight online sessions within 12 weeks. Each subsequent session will be made available to participants five days after the completion of the previous session, and participants will be encouraged to complete the next session within the week it is made available. Participants will be informed that access to iACT4CARERS will cease after 12 weeks if they have not completed all eight sessions during this period.

6.4.2 Arm B (Control)

6.4.2.1 Treatment-as-Usual (TAU)

The control is TAU alone. Participants in either group will continue to receive any routine support, such as Admiral Nurse appointments, during the trial. They will not be discouraged from seeking treatment outside of the study for ethical reasons. As per NICE guideline [NG97], TAU for carers normally consists of information, brief education, practical advice, local carer support groups and/or respite care provided by health and social care services, or relevant charities based on the needs. TAU will be monitored using a modified Client Service Receipt Inventory (modified CSRI[31]). We will not withdraw participants if they start receiving formal psychological therapy such as CBT, psychodynamic psychotherapy, systemic therapy and counselling during the trial, but this information will be monitored using the modified CSRI. We will undertake additional exploratory data analysis to assess the impact of contamination if a substantial proportion of participants have used pharmacological or psychological therapies.

6.4.2.2 Treatment Schedule

Participants will continue to receive any routine support over 12 weeks. They will receive no contact with trial therapists as described in ARM A.

6.4.3 Compliance and Adherence

To improve adherence to the intervention, the iACT4CARERS software will monitor participant progress. The iACT4CARERS software sends automatic multiple text/email reminders to participants

to prompt them to continue with the intervention (e.g. no completion of the session for 5 days, 10 days and 14 days). The iACT4CARERS software will also monitor therapist engagement and send automatic text/email reminders to therapists if they have not provided online feedback for a certain period (e.g. no feedback provided for 7 days, 10 days and 14 days). Both text and email reminders will be used to improve adherence, but participants have an option to unsubscribe from email reminders if they only wish to receive text reminders. If no actions are taken following multiple attempts, the unblinded Trial Manager or delegate at NCTU will make a follow-up phone call.

6.4.4 Concomitant Care

All participants will receive treatment as usual (routine support) for their caregiving role (e.g. Admiral Nurse appointments) regardless of randomisation into this trial.

6.4.5 Protocol Treatment Discontinuation

In consenting to the trial, participants are consenting to trial treatments, trial follow-up and data collection. However, an individual participant may stop treatment early or be stopped early for any of the following reasons:

- Adverse event;
- Inter-current illness that prevents further treatment;
- Any change in the participant’s condition that in the clinician’s opinion justifies the discontinuation of treatment;
- Death of the person with dementia;
- Withdrawal of consent for treatment by the participant.

As participation in the trial is entirely voluntary, the participant may choose to discontinue trial treatment at any time without penalty or loss of benefits to which they would otherwise be entitled. Although not obliged to give a reason for discontinuing their trial treatment, a reasonable effort should be made to establish this reason, whilst remaining fully respectful of the participant’s rights. The follow-up measure will include a question asking if the person with dementia has deceased to check for this information.

Participants who discontinue protocol treatment, for any of the above reasons, should remain in the trial for the purpose of follow-up and data analysis.

6.5 Outcomes

6.5.1 Primary Outcomes

The primary outcome is anxiety symptoms as assessed by the Generalized Anxiety Disorder-7 (GAD7[30]) at 12 weeks. Anxiety is equally or more prevalent than depression in family carers of people with dementia but is somewhat neglected in the current literature[3, 4]. A recent comprehensive meta-review of systematic reviews on nonpharmacological interventions (e.g. CBT, counselling) for dementia carers also demonstrated that the existing interventions are not effective for treating carer anxiety, while the efficacy of such interventions on carer depression is promising[32]. Our feasibility study demonstrated that iACT4CARERS is more beneficial in treating anxiety symptoms than depressive symptoms, suggesting that iACT4CARERS may have potential to overcome challenges observed in previous studies. Therefore, we will target anxiety symptoms as a primary outcome measure to fill the gap in the current literature.

6.5.2 Secondary Outcomes

Clinical outcome measures

- Patient Health Questionnaire-9 (PHQ9[33]): A 9-item self-report questionnaire assessing the severity of depressive symptoms. Both GAD7 and PHQ9 are used routinely in NHS psychological services for assessing recovery.
- Comprehensive Assessment of Acceptance and Commitment Therapy processes (CompACT[34]): A 23-item self-report questionnaire assessing psychological flexibility, which is commonly used in ACT studies.
- Experiential Avoidance in Caregiving Questionnaire (EACQ[35]): A 15-item self-report questionnaire assessing experiential avoidance (one component of psychological flexibility) in the caregiving context.
- Serious adverse events related or unrelated to the intervention: Any serious adverse events reported during the trial will be recorded.
- Satisfaction With Therapy and Therapist Scale-Revised (STTS-R[36]): A 12-item self-report questionnaire assessing satisfaction with therapy and satisfaction with the therapist.

Cost-effectiveness measures

- EQ-5D-5L[37]): A 5-item self-report questionnaire assessing health-related quality of life.
- ICEpop CAPability measure for Older people (ICECAP-O[38]): A 5-item questionnaire assessing capabilities that are required to have a high quality of life in older adults.
- Modified Client Service Receipt Inventory (modified CSRI[31]): An interview-based measure of health and social care service utilisation. This scale will be used to monitor TAU received in both arms during the trial and to calculate patient costs.

Measures of bias

Expectations about treatment and participants' intervention arm preference are potential sources of bias that can affect treatment outcomes. These measures will be collected during the baseline session prior to randomisation, after participants are given a rationale for iACT4CARERS.

- Expectations about treatment: Two questions asking how much participants expect their symptoms and life to improve if they receive iACT4CARERS will be used (collected on a five-point Likert scale from 0 to 4).
- Treatment preference: Two questions asking how much they hope to receive iACT4CARERS and TAU alone without iACT4CARERS will be used (collected on a five-point Likert scale from 0 to 4).

Intervention fidelity measures

- ACT Fidelity Measure (ACT-FM[39]): All written feedback provided online by therapists will be recorded and randomly selected scripts will be reviewed and rated by independent ACT experts using an adapted form of the ACT-FM to check for intervention fidelity.
- Therapist response time: The time gap between the participant leaving a comment and their therapist providing a response will be collected for each session. Therapists are directed to provide feedback within three working days, where possible, but no later than five working days (i.e. before the next session is made available).

6.5.3 Qualitative Study/Process Evaluation

Following the StaRI Statement[40], an evaluation of implementation, mechanism and context will be carried out to provide a clear understanding of why (un)intended outcomes occurred. The NHS Five Year Forward View emphasises investing in and promoting digital technology to improve mental health outcomes. However, we still lack understanding of how to improve digital intervention use and outcomes, particularly among those from ethnic minority groups. This process evaluation

component will enable us to evaluate the implementation and identify factors that are critical to successful implementation.

Data for evaluation of implementation

The following data will be gathered as part of the RCT to understand the process through which training and the intervention is delivered. These will be analysed descriptively by calculating total numbers, percentages and mean and standard deviation as appropriate.

- Trial therapist attendance at training and drop-in supervision sessions; scores for the intervention fidelity checklist completed by independent experts; participants' time spent for each online session (automatically tracked website logins); completion of goal setting each week (reflection section of the online programme); achievement of a goal each week (checklist embedded in the online programme); questions completed at the beginning of each online session assessing how much participants have used learnt skills between the sessions (Fidelity).
- Adaptations made to the format of training to respond to the needs of therapists with different backgrounds (Adaptations).
- Number of online sessions completed, number of weeks required to complete the whole online programme and number of optional one-to-one sessions signed up by participants; the number of sessions where participants completed the reflective comments and home practice (Dose).
- Diversity of participants in terms of age, gender, geographical location and ethnicity (Reach).

Data for evaluation of mechanisms of impact and context

Individual interviews will be conducted with participants via telephone/video call to understand the mediating factors and context that may influence implementation or outcomes. Subgroups of participants (completers, non-completers) from white, black and Asian ethnic groups will be purposefully sampled to achieve an approximate sample size of n=40, informed by the concept of Information Power[41]. All participants with insufficient understanding of English will also be invited to explore whether the use of interpreters had any impact on implementation or outcomes. Participants will be asked for their views on the strengths and limitations of iACT4CARERS and methods of delivery, the perceived impact of the intervention, internal and external barriers/facilitators for implementing the intervention in their everyday lives, the availability and competency of the therapist and any recommendations for revising the intervention.

All therapists who took part in the trial and referrers (clinicians who were involved in identifying and referring study participants at the trial sites) will be invited to be interviewed to explore factors which may influence implementation. The therapist version will explore their perceptions of delivering iACT4CARERS in practice (treatment fidelity, ease of supporting family carers online, internal and external barriers/facilitators for implementing the intervention). Qualifications and experiences of trial therapists, such as relevant degree, current job role within the NHS and previous experiences in working with people with dementia and their family carers, will also be recorded at the start of the trial. The referrer version will explore how participants were identified and referred (internal/external factors influencing recruitment).

6.6 Participant Timeline

Table 2. The schedule of enrolment, interventions and assessments

	Screening Baseline	Allocation	Intervention	Follow-up	
TIMEPOINT	-1 week	0 week	–	12 weeks[1]	24 weeks[1]
**ENROLMENT:**					
Eligibility screen	X				
Informed consent	X				
GAD7	X			X	X
Randomisation		X			
**INTERVENTIONS:**					
iACT4CARERS + TAU			←——→		
TAU alone			←——→		
**ASSESSMENTS:**					
Expectancy questionnaire	X				
Treatment preference	X				
PHQ9	X			X	X
CompACT	X			X	X
EACQ	X			X	X
STTS-R (treatment arm only)				X	
EQ-5D-5L	X			X	X
ICECAP-O	X			X	X
Modified CSRI	X			X	X
Qualitative interview				X	

[1] Follow-up assessments should be completed within two weeks of the scheduled date

6.6.1 Participant Assessments

6.6.1.1 Screening and Baseline

Upon receipt of clinician- and self-referrals (further details on the recruitment methods are provided in section 6.8.1 Recruitment), the Research Associate based at UEA will describe the study to potential participants and send the Participant Information Sheet (PIS) via email. The PIS can also be sent via post if preferred. If the post option is selected, a screening and baseline pack consisting of an information sheet, consent form, eligibility checklist, baseline questionnaires and a prepaid return envelope will be sent so that participants have access to a paper version of all documents required at the subsequent session.

Participants who opt to take part in the study will be asked to provide fully informed written consent in the presence of the Research Associate based at UEA. The Research Associate will be present via video call or phone call. If an online consent form is selected, a simple typewritten electronic signature will be used following the HRA guidance on eConsent. Participants will also be asked to provide multiple forms of contact including email, mobile, landline and postal address.

Once online written consent has been obtained, participants will be asked to complete the online screening assessment hosted by the central database (REDCap), stored on servers based at NCTU. The screening assessment includes an eligibility checklist (a series of statements asking that they meet the eligibility criteria), demographic questions and the measure of anxiety (GAD7[30]). Participants meeting eligibility criteria and scoring five or above on the GAD7 will be asked to continue completing all baseline measures. Participants will also be asked to indicate their preferred method for follow-up assessments (online or via post). To minimise missing data, participants will be unable to submit any online questionnaire with any missing fields. This will be clearly explained in the PIS. Participants can select to use the paper version via post if preferred.

If the post option is selected, participants will be asked to return the signed consent form and the completed eligibility checklist, the GAD7, socio-demographic questions and baseline measures using a prepaid envelope. The Research Associate based at UEA will manually enter the data into the central database upon the receipt of documents.

6.6.1.2 Intervention

The eligibility criteria will be fully checked within a week from the completion of baseline assessments, and eligible participants will be randomised to iACT4CARERS plus TAU or TAU alone by NCTU. Participants randomised to iACT4CARERS plus TAU will receive a link to the iACT4CARERS website and login details from the unblinded Trial Manager based at NCTU via email (and post if requested). Unauthorised access to the intervention will be prevented by providing participants with unique login details. The allocated trial therapist will also receive a notification from the unblinded Trial Manager based at NCTU via email.

6.6.1.2 12-week and 24-week Follow-Ups

Twelve and twenty-four weeks after randomisation, participants will receive an email to ask them to complete the follow-up measure online from NCTU, if they have requested this. The email will include a reminder, which reiterates the reasons why this follow-up data is important. If the follow-up measure is not completed within a week, the Research Associate based at UEA who is blind to treatment allocation will be informed by NCTU and will give a follow-up phone call to check if the participant needs additional support for completing the online assessments. If the participant

decides not to complete the assessments, the Research Associate will ask them to provide a reason if possible. Participants, who have indicated that they wish to receive the follow-up measure via post, will receive the follow-up assessments via post from NCTU and will be asked to return them to NCTU using a prepaid envelope. The unblinded Trial Manager based at NCTU will give a follow-up phone call a week after the assessments have been posted to ensure that participants have received the follow-up measure and check if additional support is needed for completion. The unblinded Trial Manager based at NCTU will manually enter the data into the central database upon the receipt of documents. Participants will be asked not to reveal their allocation to the blinded Research Associate during follow-up calls.

Selected participants from the intervention arm will be invited to an individual interview at a 12-week follow-up as part of the process evaluation. The interview is optional, and participants will receive a separate PIS and consent form for this part of the study if invited. Interviews will be conducted by the unblinded delegate based at NCTU via video call or telephone. Trial therapists and referrers will also be invited to interviews (see 6.5.3 Qualitative Study/Process Evaluation). If the interview is conducted by video call, the sound recording of the interview through the video conference software will be used to audio record the session. If the interview is conducted by telephone, a digital voice recorder will be used. An audio file of interview will be uploaded to a secure server at UEA immediately after the interview, and the original recording will be completely deleted from the software or voice recorder. The recording will be transcribed as soon as possible using software approved by UEA (e.g. EDCaption) or the external transcribing company. The audio files uploaded to the secure server at UEA will be deleted as soon as the accuracy of the transcripts has been confirmed.

6.6.2 Early Stopping of Follow-up

If a participant chooses to discontinue their trial treatment, they should continue to be followed up as closely as possible to the follow-up schedule defined in the protocol, providing they are willing. They should be encouraged and facilitated not to leave the whole trial, even though they no longer take the trial treatment. If, however, the participant exercises the view that they no longer wish to be followed up, this view must be respected, and the participant withdrawn entirely from the trial. NCTU should be informed of the withdrawal in writing using the appropriate iACT4CARERS trial documentation. Data already collected will be kept and included in analyses according to the intention-to-treat principle for all participants who stop follow-up early.

Participants who stop trial follow-up early will not be replaced.

6.6.3 Participant Transfers

UEA will obtain written consent from all participants, and UEA and NCTU will conduct all assessments remotely. Therefore, participants moving to a different area of England during the trial will not affect data collection. Participant transfers will not occur during the trial.

6.6.4 Loss to Follow-up

Multiple methods of contact will be collected before the screening session (email, mobile/landline, post) to enable us to send multiple follow-up reminders.

6.6.5 Trial Closure

The end of the trial is defined as four months following the last follow-up visit of the last participant randomised, to allow for data entry and data cleaning activities to be completed.

6.7 Sample Size

Participants in our feasibility study had an average reduction of 26% on GAD7. A reduction of 20% on GAD7 is considered to be a Minimum Clinically Important Difference[23]. Using a conservative reduction of 20% from the mean baseline GAD7 score, and using the variability estimated from our feasibility study, a clinically-meaningful effect size is estimated to be $d = 0.55$ for our sample. Recent systematic reviews of RCTs of self-help ACT and mindfulness-based interventions (mainly for individuals with a mental or physical health condition) demonstrated a pooled effect size of $d = 0.35$ for anxiety[42, 43]. One of these reviews, which solely focused on RCTs of self-help ACT[42] demonstrated that when analysis was limited to studies that utilised guided self-help ACT, the pooled effect size increased to $d = 0.61$, while limiting analysis to studies that used an unguided approach decreased the pooled effect size to $d = 0.16$. The effect size calculated from our feasibility study of iACT4CARERS, which utilised a guided approach, is in line with the effect size reported in previous reviews.

To ensure adequate statistical power, we have decided to take the most conservative approach possible and have used an effect size of $d = 0.35$ in our sample size calculation. To detect the effect size of $d = 0.35$ between the two trial arms at a two-tailed significant level of 5% with 90% statistical power, each arm requires 173 participants (i.e. 346 in total). Twenty seven percent of carers were lost to follow-up in our feasibility study. Assuming a dropout rate of 30%, a total of 496 carers will be recruited over 20 months (i.e. 24.8 participants per month).

6.8 Recruitment and Retention

6.8.1 Recruitment

In our feasibility study, 76% of participants who received iACT4CARERS had self-referred from the community, and 24% of participants were referred from NHS services[22]. Therefore, multiple recruitment strategies will be used to reach out to potential participants, and both clinician and self-referrals will be accepted.

- Recruitment through NHS PICs: PICs are most likely to be primary carer services (GPs). PICs will search their database to identify potentially eligible participants. An invitation letter, including PIS, an expression of interest card and a pre-paid return envelope, will be sent to potential participants from PICs. If interested, participants will be asked to return the expression of interest card, providing their preferred contact detail, to the study team at UEA using the return envelope. Carer-focused publicity materials (study flyer and video-recorded study advertisement) will also be made available in waiting areas of the services and on their social media platforms (e.g. twitter, Facebook, website) to promote the study.
- Recruitment through NHS trial sites: Potential participants will be identified through trial sites. These services will include Community Mental Health Teams and memory clinics. Clinicians will approach potentially eligible participants and will seek verbal consent for Research Associates based at UEA to contact the participant with further information about the study if they express interest. A nominated individual at trial sites will share contact detail of potential participants (name, telephone number and email) with UEA via the central database (REDCap), stored on

servers based at NCTU. The central database will also ask the nominated individual to indicate that they have checked the basic eligibility criteria (being a family carer, interested in online psychological support and having access to internet) and have obtained verbal consent. Research Associates will then arrange a screening appointment. Carer-focused publicity materials (study flyer and video-recorded study advertisement) will also be made available in waiting areas of the services and on their social media platforms (e.g. twitter, Facebook, website) for those who may wish to self-refer.

- Recruitment within the community: Various methods (used successfully in the feasibility study) will be used in the community to reach potential participants. This includes advertisement through local newspapers (the most successful strategy in our feasibility study) and Join Dementia Research. The study will also be advertised through relevant charities (e.g. Alzheimer's Society) and local community services (e.g. dementia cafés). Carer-focused publicity materials (study flyer and video-recorded study advertisement) will be made available through the relevant resources such as their newsletters and social media platforms (e.g. twitter, Facebook, website). These approaches will allow potential participants to self-refer to the trial via telephone, email, or the iACT4CARERS website.
- Culturally acceptable recruitment strategies: Multiple strategies recommended by our PPI members will be used to maximise the recruitment of participants from ethnic minority groups. These include advertising on TV programmes that target ethnic minority groups, such as Colors and Start Plus, visiting places of worship, contacting local faith leaders, and circulating the information through relevant social media (e.g. Facebook, twitter) and WhatsApp befriending groups which have become popular during the COVID-19 pandemic. Setting up these approaches will be supported by the collaborating partner, the Centre for Ethnic Health Research. A video-recorded study advertisement in three different languages will be produced by the Centre for Ethnic Health Research. Potential participants will be able to self-refer to the trial via telephone, email, or the iACT4CARERS website.

6.8.2 Retention

We will minimise loss to follow-up in a number of ways. Our feasibility study demonstrated that the content of iACT4CARERS was acceptable and positively received by family carers[44], although 27% of carers were lost to follow-up[22]. Thus, we have inflated our sample size estimate to allow for a dropout rate of 30%. We will include detailed explanations in the information sheet to explain to participants why data completion at follow-up is critical. Multiple methods of contact will be collected before the screening session (email, mobile/landline, post) to enable us to send multiple follow-up reminders. Furthermore, we will use various strategies to maintain study participation and encourage participants to complete outcome measures such as: use of incentives (participants will be entered into a prize draw to win one of 50 £20 gift vouchers for completing follow-up assessments), contacting people prior to outcome assessments, sending greetings cards, and maintaining contact through study newsletters. The retention will be reviewed an ongoing basis by the Trial Management Group (TMG) and the Data Monitoring and Ethics Committee (DMEC) to flag a dropout rate of >30% and if mitigating steps need to be taken.

6.9 Assignment of Intervention

6.9.1 Allocation

6.9.1.1 Sequence generation

Following baseline assessments, participants will be randomised on a 1:1 basis between iACT4CARERS plus TAU or TAU alone. A computerised centralised randomisation system managed by NCTU will be used. The allocation will be generated via computer written code using minimisation. Minimisation will be performed with the factors collected at baseline: GAD7 severity range (mild, moderate, severe) and ethnicity (Asian, Black, White, Mixed or Other ethnic groups).

6.9.1.2 Allocation concealment mechanism

Allocation will be computer-generated by a centralised system managed by NCTU ensuring concealment prior to randomisation. The sequence will be hidden from users of the database. Once allocated, a secure email will be sent to the Trial Manager and Research Lead at NCTU who will be unblinded. An additional email with blinded notification of randomisation will be sent to the Research Associates (outcome assessors) and the Principal Investigator. The allocation will not be exposed to any other users of the database.

6.9.1.3 Allocation Implementation

The unblinded Trial Manager based at NCTU will be informed of the allocation. The Trial Manager will set up the account for the online intervention for participants randomised to iACT4CARERS plus TAU and will send an email containing a link to the iACT4CARERS website and unique login details to them. Participants recruited at trial sites will be assigned to the trial therapist from the same site. Participants recruited from other resources (e.g. self-referral from the community) will be randomly assigned to one of the trial therapists across trial sites. The allocated trial therapist will also receive a notification from the unblinded Trial Manager via email. Research Associates (outcome assessors) will not have access to the allocation at any time during the study.

6.9.2 Blinding

All unblinded study procedures will be conducted by the unblinded Trial Manager, CTU Research Lead or the authorised delegate based at NCTU. Research Associates based elsewhere at UEA collecting baseline and follow-up data will be blinded to group allocation. Outside of the assessments, Research Associates will be shielded from discussion of participants where the possibility of determining the allocation group of the participants could occur. A system of web-based central data entry will ensure that Research Associates will not have access to information in the database that would reveal the allocation group.

6.9.3 Emergency Unblinding

As the intervention and control are delivered unblinded to the Trial Manager, CTU Research Lead and Chief Investigator, and trial therapists are aware as to participants randomised to intervention, no emergency unblinding procedures are required.

6.10 Data Collection, Management and Analysis

6.10.1 Data Collection Methods

Each participant will be given a unique trial Participant IDentification Number (PID). Data will be collected at the time-points indicated in the Trial Schedule (see Table 2, section 6.6).

Since all questionnaires are self-reported, the preferred method of data collection is direct online entry of data onto the central database, stored on servers based at NCTU by participants using an online version of Case Record Forms (CRFs). If participants request paper CRFs, these will be sent via post with a prepaid return envelope by Research Associates based at UEA for baseline and by the unblinded Trial Manager based at NCTU for follow-ups. Research Associates or the Trial Manager will then manually enter the data into the database. The relevant individuals will receive training on data collection and use of the online system. Paper CRFs will be kept at UEA (baseline) and NCTU (follow-ups) in a locked cabinet within a secured room.

Data collection, data entry and queries raised by a member of the iACT4CARERS trial team will be conducted in line with the NCTU and trial specific Data Management Standard Operating Procedure. Screening and enrolment logs that do not contain participant identifiable data (e.g. data of consent, date of screening, eligibility for enrolment) will be entered to the central database stored (REDCap) on the servers based at NCTU by Research Associates and will be kept electronically.

A participant database, linking participant identifiable data to the pseudo anonymised PID, will be password protected and stored on servers based at NCTU. The participant database will be updated and maintained by Research Associates and Trial Manager. Access to the participant database will be limited to the central trial team for the purpose of contacting participants and sending questionnaires and newsletters during the trial. There will be a clear logical separation of participant identifiable data from the trial data. Trial team members will receive trial protocol training. All data will be handled in accordance with the Data Protection Act 2018.

6.10.2 Data Management

Data will be entered under the participants PID number onto the central database stored on the servers based at NCTU. Access to the database will be via unique, individually assigned (i.e. not generic) usernames and passwords, and only accessible to members of the iACT4CARERS trial team at NCTU, and external regulators if requested. The servers are protected by firewalls and are patched and maintained according to best practice. The physical location of the servers is protected physically and environmentally in accordance with University of East Anglia's General Information Security Policy 3 (GISP3: Physical and environmental security).

The database and associated code have been developed by NCTU Data Management, in conjunction with the iACT4CARERS trial team. The database software provides a number of features to help maintain data quality, including; maintaining an audit trail, allowing custom validations on all data, allowing users to raise data query requests, and search facilities to identify validation failure/ missing data.

After completion of the trial the database will be retained on the servers of NCTU for on-going analysis of secondary outcomes.

The screening log and enrolment log that do not contain participant identifiable data will be kept electronically in the central database stored on servers based at NCTU. A participant database, linking participant identifiable data to the pseudo anonymised PID, will be password protected and stored on servers based at NCTU separately. After completion of the trial the screening and enrolment logs will be stored securely by NCTU for 10 years.

6.10.3 Non-Adherence and Non-Retention

All data will be recorded irrespective of participant adherence. Should a participant withdraw consent or be lost to follow-up, all data will be included in the study dataset up to the point of consent withdrawal or loss to follow-up and will be included in the study database. Reason for withdrawal will be recorded, if given, as will loss to follow-up.

6.10.4 Statistical Methods

6.10.4.1 Outcomes

In the primary analysis, which will be by intention to treat, linear mixed models with random intercepts for site and therapist will be used to estimate group differences in total score on GAD7. Score at 12- and 24-week follow-ups will be used as the dependent variables. The treatment group will be used as the independent variable. Baseline outcome score, the research site and ethnicity will be used as the covariates. Sensitivity analyses will be used to identify any variations or methods (e.g. outliers, therapists, the number of sessions completed) that may influence the findings. Secondary outcome measures will be analysed in a similar fashion to the primary outcome measure. The impact of contamination due to non-compliance as well as participants receiving treatments that are not part of the trial (e.g. psychological therapy, pharmacological therapy) will be assessed by using CACE analysis where information on any extra-trial treatments will be incorporated as covariates in the CACE approach.

Statistical significance will be set at the conventional (2-tailed) 5% level and all parameter estimates will be presented with 95% confidence intervals. Analyses will be carried out by the trial statistician blinded to group allocation.

6.10.4.2 Statistical Analysis Plan

A statistical analysis plan (SAP) will be developed between the trial statistician and the Chief Investigator and agree with the independent Data Monitoring and Ethics Committee (DMEC) and Trial Steering Committee (TSC). This will be prior to database lock and any data analysis.

6.10.4.3 Additional Analyses

No subgroup analyses are planned. During the trial, specific sub-groups may be suggested possibly as the result of new information becoming available, but any analyses will be agreed and stated in the SAP.

6.10.4.4 Analysis Population

The analyses population are defined as:

- Intention to treat (ITT): All randomised individuals regardless of adherence. We will adopt appropriate techniques for handling missing data depending on the mechanism of missingness as described in section 6.10.4.5 Missing data.
- If compliance is less than 80%, a CACE analysis will be carried out defining compliance as participant in the treatment arm completing at least six or more sessions.

6.10.4.5 Missing Data

Participants will be unable to submit any questionnaire with any missing fields when an online version of CRFs is used. Therefore, there will be no missing data from questionnaires completed online. This will be clearly explained in the information sheet. There may be some missing data when

participants choose to use paper CRFs. Also, some missing data may result from loss to follow-up. A small percentage (5% or less) of missing data is generally inconsequential[45] in which case we will carry out analysis of available data without imputation. If more than 5% of data is missing, we will adopt appropriate technique for handling missing data depending on the mechanism of missingness. Specifically, if the mechanism of missing data is found to be missing at random (MAR), i.e., missingness is predicted by observed covariates, we will employ multiple imputation to handle missing data[46], where the imputation model will include observed predictors of missing data. The estimates obtained from the analysis of imputed datasets will be combined using Rubin's rule[47].

6.10.5 Economic evaluations

The trial will include an economic evaluation from an NHS and social care perspective, as recommended by NICE[48]. Both costs and outcomes will relate to the carer. The main outcome measure would be cost per QALY where QALYs are measured using the EQ-5D-5L, this would constitute a cost-utility analysis. The EQ-5D-5L will be scored using the recommend scoring algorithm at the time of analysis, currently NICE recommends the use of the 'cross-walk' algorithm[49]. The EQ-5D is recommended for use in economic evaluations by NICE[48] and it has been used in a number of studies to estimate HRQoL for carers[50]. Sensitivity analyses would include using the ICECAP-O to generate an alternative preference-based outcome measure, and also conducting a cost-effectiveness study using the study primary outcome measure (cost per point change in GAD7).

A variety of costs will be estimated as part of the study. Firstly, it is important to estimate the cost of providing the intervention. This will include resources required to provide the online sessions and would include set up cost as well as any costs for maintaining or updating the site. However, it is likely that costs associated with providing this service in the current trial would differ to how this service would be provided in future clinical practice. This could include changes in how the service is provided, who provides it, and how many clients it is offered to. For these reasons, a sensitivity analysis will be conducted exploring the effect of a variety of different assumptions on the costs of providing iACT4CARERS. We will also record resources required to provide therapist support. These will include the cost of providing required training (2-day workshop), time required to provide supervision by the clinical psychologist, the number of direct contact sessions provided to participants and time spend responding to any participant questions. Secondly, we will record resources associated with health and social care received by the participant (carer). These will be obtained using a modified CSRI, administered at baseline, 12, and 24 weeks. To facilitate recall over the 12-week recall period we will provide participants with simple diary sheets to record health care events to act as a mnemonic. All resources identified will be costed using appropriate unit cost data[51]. As the follow-up in this study is 24-weeks we will not discount costs or benefits.

As part of the analysis process the degree of missingness of the data will be assessed. If there are low levels of missing data, then a complete case analysis may be appropriate. If, however, missingness is deemed to be a potential problem then appropriate methods for dealing with these will be employed[52]. *A priori*, we would expect that this would involve multiple imputation techniques. We would expect to use regression-based methods to analyse costs and effects to allow for differences in baseline characteristics between groups. Non-parametric bootstrapping will be employed to allow for uncertainty. We will estimate cost-effectiveness acceptability curves to estimate the probability that the intervention is cost-effective at different willingness to pay for a

unit of the outcome measure. As part of the analysis process, we would also conduct appropriate sensitivity analysis to estimate the effects of any key assumptions made. Any such sensitivity analysis will be pre-specified in a health economics analysis plan (HEAP).

6.10.5.1 Health Economic Analysis Plan

Prior to analysis, the lead health economist will develop the HEAP pre-specifying the intended analysis. This will be agreed with the Chief Investigator and study statistician. It will be shared with the DMEC and TSC for comments.

6.10.6 Analysis of Qualitative Information

Interviews will be transcribed verbatim, checked, and anonymised. Participant, therapist and referrer transcripts will be analysed separately using a focused thematic analysis[53]. Three coders will independently read through transcripts, separate the data into meaningful fragments and label emerging themes with codes. Coding strategies will be compared with instances of disagreement discussed until a provisional conceptual framework is developed around internal and external factors influencing the outcomes and implementation. The analytical framework will be applied to the remaining transcripts, with themes and subthemes refined as necessary. The computer programme NVivo will facilitate analysis, enabling coding and retrieval of a large volume of narrative data.

6.11 Data Monitoring

6.11.1 Data Monitoring and Ethics Committee

The Data Monitoring and Ethics Committee (DMEC) consisting of independent experts will have access to trial data and meet bi-annually or annually as required to review safety data. Further details of the roles and responsibilities of the Data Monitoring and Ethics Committee (DMEC), including membership, relationships with other committees, decision making processes, and the timing and frequency of interim analyses (and description of stopping rules and/or guidelines where applicable) are described in detail in the iACT4CARERS DMEC Terms of Reference (ToR).

6.11.2 Interim Analyses

No efficacy interim analyses are planned. However, analysis of recruitment, withdrawal and adherence rates will be conducted at intervals during the trial.

6.11.3 Data Monitoring for Harm

6.11.3.1 Safety reporting

Definitions of harm of the EU Directive 2001/20/EC Article 2 based on the principles of ICH GCP apply to this trial.

This is a low-risk intervention. No specific risks, or adverse events were reported during feasibility testing of the intervention[22]. Any non-serious untoward incidents occurring during this trial (e.g. clear evidence of high levels of distress making participants difficult to continue with the study) will be recorded in an incident report form which will be monitored for frequency and severity. The participant's GP will also be informed to contact NCTU if they become aware of any adverse event during the study such as significant deterioration in mental health and they feel that the participant should no longer be taking part in the study. Serious adverse events (SAE) will be collected using an SAE form.

Table 3: Serious Adverse Event Definitions

**Serious Adverse Event (SAE)**	Any adverse event that:<br>• results in death;<br>• is life threatening*;<br>• requires hospitalisation or prolongs existing hospitalisation**;<br>• results in persistent or significant disability or incapacity.<br><br>The most likely SAE to occur in this study is new reports of active suicidal ideation with active suicidal behaviours/plans and imminent intent.
* The term life threatening here refers to an event in which the patient is at risk of death at the time of the event; it does not refer to an event that might hypothetically cause death if it was more severe (e.g. a silent myocardial infarction).<br><br>** Hospitalisation is defined as an in-patient admission, regardless of length of stay, even if the hospitalisation is a precautionary measure for continued observation. Hospitalisation for pre-existing conditions (including elective procedures that have not worsened) do not constitute an SAE.	

Serious adverse events do NOT include:

- Pre-existing disease or a physical health condition present before trial treatment;
- An exacerbation of such pre-existing physical illness (e.g. cancer) during the trial;
- A physical health condition that is detected after trial treatment.

6.11.3.2 Other Notifiable Adverse Events
None

6.11.3.5 Investigator responsibilities relating to safety reporting
All non-serious untoward incidents should be reported to NCTU and recorded in the database within seven days. SAEs should be notified to NCTU immediately the investigator becomes aware of the event (in no circumstance should this notification take longer than one working day).

6.11.3.5.1 Seriousness assessment
When an AE occurs, the individual responsible for the care of the participant (Research Associate or trial therapist) must first assess whether or not the event is serious in conjunction with the Chief Investigator (if AE is identified by Research Associate) or the Principal Investigator (if AE is identified by trial therapist) using the definition given in Table 3. If the event is classified as 'serious' then an SAE form must be completed, and the Trial Manager notified within one working day.

6.11.3.5.2 Severity or grading of Adverse Events
The severity of all AEs (serious and non-serious) will not be graded in this trial.

6.11.3.5.3 Causality

The individual responsible for the care of the participant (Research Associate or trial therapist) must assess the causality of all serious events in relation to the trial therapy in conjunction with the Chief Investigator or the Principal Investigator using the definitions in Table 4.

Table 4: Causality definitions

Relationship	Description	Event type
Unrelated	There is no evidence of any causal relationship.	Unrelated SAE
Unlikely to be related	There is little evidence to suggest that there is a causal relationship (e.g. the event did not occur within a reasonable time after administration of the trial intervention). There is another reasonable explanation for the event (e.g. the participant's clinical condition or other concomitant treatment).	Unrelated SAE
Possibly related	There is some evidence to suggest a causal relationship (e.g. because the event occurs within a reasonable time after administration of the trial intervention). However, the influence of other factors may have contributed to the event (e.g. the participant's clinical condition or other concomitant treatment).	Related SAE
Probably related	There is evidence to suggest a causal relationship and the influence of other factors is unlikely.	Related SAE
Definitely related	There is clear evidence to suggest a causal relationship and other possible contributing factors can be ruled out.	Related SAE

6.11.3.5.4 Expectedness

If there is at least a possible involvement of the trial procedures (including any comparators), the Chief Investigator, the Principal Investigator and sponsor must assess the expectedness of the event.

6.11.3.6 Notifications

6.11.3.6.1 Notifications by the Investigator to NCTU

NCTU must be notified of all SAEs within one working day of a responsible individual (Research Associate or trial therapist) becoming aware of the event. The responsible individual should notify NCTU of any SAEs occurring from the time of randomisation until the last follow-up assessment.

The SAE form must be completed by the responsible individual (Research Associate or trial therapist) with attention paid to the causality and expectedness of the event. The responsible investigator (the Chief Investigator for Research Associates and the Principal Investigator for trial therapists) should check the SAE form at the earliest opportunity, make any changes necessary, sign and then email to the Trial Manager at NCTU within one working day for review.

The Trial Manager will review the SAE form and disseminate to the Chief Investigator and sponsor representative within 72 hours of being informed to assess relatedness. The Trial Manager will enter the report into the database. The DMEC will be informed of SAEs by the Trial Manager periodically unless the Chief Investigator or sponsor representative escalates the SAE or deems necessary. This is a non-CTIMP study and only SAEs that are related and unexpected will be reported to REC within 15 days of the Chief Investigator becoming aware of the event.

The minimum criteria required for reporting an SAE are the trial number and date of birth, name of reporting investigator and sufficient information on the event to confirm seriousness. Any further information regarding the event that is unavailable at the time of the first report should be sent as soon as it becomes available.

Follow-up SAE forms (clearly marked as follow-up) should be completed and emailed to NCTU, as per above procedure. Additional information and/or copies of test results etc may be provided separately. The participant must be identified by trial number, date of birth and initials only. The participant's name should not be used on any correspondence and should not be used on any correspondence.

6.11.3.6.2 NCTU responsibilities

Medically qualified staff at NCTU and/or the Chief Investigator will review all SAE reports received. In the event of disagreement between the causality assessment given by the Principal Investigator and the Chief Investigator, both opinions and any justifications will be provided in subsequent reports.

NCTU will keep investigators informed of any safety issues that arise during the course of the trial. NCTU will also submit annual reports to the REC.

6.11.4 Quality Assurance and Control

6.11.4.1 Risk Assessment

The Quality Assurance (QA) and Quality Control (QC) considerations for the iACT4CARERS trial are based on the standard NCTU Quality Management Policy that includes a formal Risk Assessment, and that acknowledges the risks associated with the conduct of the trial and proposals of how to mitigate them through appropriate QA and QC processes. Risks are defined in terms of their impact on: the rights and safety of participants; project concept including trial design, reliability of results and institutional risk; project management; and other considerations.

QA is defined as all the planned and systematic actions established to ensure the trial is performed and data generated, documented and/or recorded and reported in compliance with the principles of

GCP and applicable regulatory requirements. QC is defined as the operational techniques and activities performed within the QA system to verify that the requirements for quality of the trial related activities are fulfilled.

6.11.4.2 Central Monitoring at NCTU

NCTU staff will review Case Report Form (CRF) data for errors and missing key data points. The trial database will also be programmed to generate reports on errors and error rates. Essential trial issues, events and outputs, including defined key data points, will be detailed in the iACT4CARERS trial Data Management Plan.

6.11.4.3 On-site Monitoring

The frequency, type and intensity of routine and triggered on-site monitoring will be detailed in the iACT4CARERS Quality Management and Monitoring Plan (QMMP). The QMMP will also detail the procedures for review and sign-off of monitoring reports. In the event of a request for a trial site inspection by any regulatory authority, NCTU must be notified as soon as possible.

6.11.4.3.1 Direct access to participant records

Participating investigators must agree to allow trial related monitoring, including audits, REC review and regulatory inspections, by providing access to source data and other trial related documentation as required.

6.11.4.4 Trial Oversight

Trial oversight is intended to preserve the integrity of the trial by independently verifying a variety of processes and prompting corrective action where necessary. The processes reviewed relate to participant enrolment, consent, eligibility, and allocation to trial groups; adherence to trial interventions and policies to protect participants, including reporting of harms; completeness, accuracy and timeliness of data collection; and will verify adherence to applicable policies detailed in the Compliance section of the protocol. Independent trial oversight complies with the NCTU trial oversight policy.

In multi-centre trials this oversight is considered and described both overall and for each recruiting centre by exploring the trial dataset or performing site visits as described in the iACT4CARERS Quality Management and Monitoring Plan.

6.11.4.4.1 Trial Team

The Trial Team will be set up to assist with developing the design, co-ordination and day to day operational issues in the management of the trial, including budget management. The membership, frequency of meetings, activity (including trial conduct and data review) and authority will be covered in the Trial Team terms of reference.

6.11.4.4.2 Trial Management Group

A Trial Management Group (TMG) will be set up to assist with developing the design, co-ordination and strategic management of the trial. The membership, frequency of meetings, activity (including trial conduct and data review) and authority will be covered in the TMG terms of reference.

6.11.4.4.3 Independent Trial Steering Committee

The Independent Trial Steering Committee (TSC) is the independent group responsible for oversight of the trial in order to safeguard the interests of trial participants. The TSC provides advice to the Chief

Investigator, NCTU, the funder and sponsor on all aspects of the trial through its independent Chair. The membership, frequency of meetings, activity (including trial conduct and data review) and authority will be covered in the TSC terms of reference.

6.11.4.4.4 Independent Data Monitoring and Ethics Committee

The Independent Data Monitoring and Ethics Committee (DMEC) is the only oversight body that has access to unblinded accumulating comparative data. The DMEC is responsible for safeguarding the interests of trial participants, monitoring the accumulating data and making recommendations to the TSC on whether the trial should continue as planned. The membership, frequency of meetings, activity (including review of trial conduct and data) and authority will be covered in the DMEC terms of reference. The DMEC will consider data in accordance with the statistical analysis plan and will advise the TSC through its Chair.

6.11.4.4.5 Trial Sponsor

The University of East Anglia is the trial sponsor. The role of the sponsor is to take on responsibility for securing the arrangements to initiate, manage and finance the trial. The Sponsor is responsible for ensuring that the study meets the relevant standards and makes sure that arrangements are put and kept in place for management, monitoring and reporting. The University of East Anglia has delegated responsibility for the overall management of the iACT4CARERS trial to the Chief Investigator and NCTU.

7 Ethics and Dissemination

7.1 Research Ethics and Health Research Authority Approval

Before initiation of the trial at any clinical site, the protocol, all informed consent forms and any material to be given to the prospective participant will be submitted to the Health Research Authority (HRA) and Research Ethics Committee for approval. Any subsequent amendments to these documents will be submitted for further approval.

The rights of the participant to refuse to participate in the trial without giving a reason must be respected. Participants in either group will continue to receive any routine support, such as Admiral Nurse appointments, during the trial. They will not be discouraged from seeking treatment outside of the study for ethical reasons. Following the baseline assessment, the participant's GP will also be informed of their involvement in the study, and any concerns raised by their GP regarding their involvement will be followed by a full discussion with the participant. After randomisation the participant must remain within the trial for the purpose of follow-up and data analysis according to the treatment option to which they have been allocated. However, the participant remains free to change their mind at any time about the protocol treatment and follow-up without giving a reason and without prejudicing their further treatment.

7.2 Other Approvals

Documentation will need to be submitted to the R&D Department at each NHS Site in order to gain confirmation of capacity and capability prior to the study being initiated at that site. Confirmation from the site will take the form of a site agreement signed by both NCTU and the relevant site.

The protocol has received formal approval and methodological, statistical, clinical and operational input from the NCTU Protocol Review Committee.

7.4 Amendments

Amendments to the Protocol and other documents (e.g. changes to eligibility criteria, outcomes, sample size calculations, analyses) will be agreed by the TMG. Such amendments will be forwarded to the Sponsor for confirmation as to whether it is either substantial or non-substantial and will then be submitted to the Health Research Authority (HRA) or Ethics Committee for categorisation and approval. Once the amendment has been categorised it will be sent to relevant sites for consideration in accordance with standard HRA processes and timescales. Amendments must not be implemented until HRA approval is received and sites have either confirmed acceptance or, no objection has been received within the defined timescale. Notification will be sent by NCTU to trial personnel to confirm when an amendment can be implemented.

7.5 Consent or Assent

Patients will be provided with a Participant Information Sheet (PIS) and given time to read it fully. Following a discussion with a Research Associate or suitable trained and authorised delegate, any questions will be satisfactorily answered and if the participant is willing to participate, written informed consent will be obtained electronically or via post. During the consent process it will be made completely and unambiguously clear that the participant is free to refuse to participate in all or any aspect of the trial, at any time and for any reason, without incurring any penalty or affecting their treatment.

Consent will be re-sought if new information becomes available that affects the participant's consent in any way. This will be documented in a revision to the PIS and the participant will be asked to sign an updated consent form. These will be approved by the ethics committee prior to their use.

A copy of the approved consent form is available from the NCTU trial team.

7.5.1 Consent or Assent in Ancillary Studies

Participants may be invited to other research activities that are not part of this trial in the future such as extended follow-up studies. These studies will be discussed and consented to separately on a project-by-project basis. Participants will have the option to indicate whether they would be willing to be informed about additional research activities in the future on the consent form.

Anonymised data may be shared with other researchers to support other research in the future. This is clearly stated in the PIS and consent form. Similar adherence to the Data Protection Act is required from all collaborators.

7.6 Confidentiality

Any paper copies of personal trial data will be kept at UEA in a secure location with restricted access. Following consent, identifiable data will be kept on the participant database to allow authorised members of the trial team to contact participants in order to arrange appointments/assessments. Only authorised trial team members will have password access to this part of the database. This information (personal data such as contact details) will be securely destroyed 5 years after the end of the trial.

Confidentiality of participant's personal data is ensured by not collecting participant names on CRFs and limiting access to personal information held on the database at NCTU. At trial enrolment the participant will be issued a participant identification number, and this will be the primary identifier for the participant, with secondary identifiers of month and year of birth and initials.

The participant's consent form will carry their name and signature. These will be kept at UEA, with a copy sent to NCTU upon request for monitoring purposes. This copy will be destroyed once checks are complete. Consent forms will not be kept with any additional participant data.

7.7 Declaration of Interests

The investigators named on the protocol have no financial or other competing interests that impact on their responsibilities towards the scientific value or potential publishing activities associated with the trial.

7.8 Indemnity

The sponsor (UEA) has appropriate insurance policies in place to provide professional indemnity and public liability cover.

7.9 Finance

iACT4CARERS is fully funded by an NIHR Health Technology Assessments grant number NIHR150071. It is not expected that any further external funding will be sought.

7.10 Archiving

The investigators agree to archive and/or arrange for secure storage of iACT4CARERS trial materials and records for 10 years after the close of the trial unless otherwise advised by the NCTU.

7.11 Access to Data

Requests for access to trial data will be considered, and approved in writing where appropriate, after formal application to the TMG and TSC. Considerations for approving access are documented in the TMG and TSC Terms of Reference.

7.12 Ancillary and Post-trial Care

The sponsor does not intend to provide any interventions or other care to participants after trial completion.

7.13 Publication Policy

7.13.1 Trial Results

The results of the trial will be disseminated regardless of the direction of effect.

7.13.2 Authorship

Ownership of the data arising from the study resides with the sponsor. The publication policy will be in line with rules of the International Committee of Medical Journal Editors. The TMG will decide on authorship with any difficulties being resolved by the TSC.

7.13.3 Reproducible Research

The iACT4CARERS Trial Protocol will be published and made available for public access.

8 Ancillary Studies

No ancillary studies are currently planned. Participants may be invited to other research activities that are not part of this trial in the future such as extended follow-up studies. These studies will be discussed and consented to separately on a project-by-project basis. Participants will have the option to indicate whether they would be willing to be informed about additional research activities in the future on the consent form.

9 Protocol Amendments

This is the first version of the protocol and no amendments have yet been made to it.

10 References

1. Chan, A.W., et al., *SPIRIT 2013 statement: defining standard protocol items for clinical trials.* Annals of Internal Medicine, 2013. **158**(3): p. 200-207.
2. Chan, A.W., et al., *SPIRIT 2013 explanation and elaboration: guidance for protocols of clinical trials.* BMJ : British Medical Journal, 2013. **346**: p. e7586.
3. Collins, R. and N. Kishita, *Prevalence of depression and burden among informal caregivers of people with dementia: A meta-analysis.* Ageing and Society, 2020. **40**(11): p. 2355-2392.
4. Kaddour, L. and N. Kishita, *Anxiety in informal dementia caregivers: a meta-analysis of prevalence.* Journal of Geriatric Psychiatry and Neurology, 2020. **33**(3): p. 161-172.
5. McManus, S., et al., eds. *Mental health and wellbeing in England: Adult psychiatric morbidity survey 2014*. 2016, NHS Digital: Leeds, UK.
6. National Institute for Health and Care Excellence, *NICE guideline [NG97] Dementia: assessment, management and support for people living with dementia and their carers*. 2018, NICE: London, UK.
7. Alzheimer's Society, *Dementia 2014: Opportunity for change*. 2014, Alzheimer's Society: London, UK.
8. Livingston, G., et al., *Clinical effectiveness of a manual based coping strategy programme (START, STrAtegies for RelaTives) in promoting the mental health of carers of family members with dementia: pragmatic randomised controlled trial.* BMJ (Clinical Research Ed.), 2013. **347**: p. f6276-f6276.
9. Hopkinson, M.D., et al., *Cognitive behavioral therapy for depression, anxiety, and stress in caregivers of dementia patients: A systematic review and meta-analysis.* The Gerontologist, 2019. **59**(4): p. e343-e362.
10. Scott, J.L., et al., *Caring for the carer: a systematic review of pure technology-based cognitive behavioral therapy (TB-CBT) interventions for dementia carers.* Aging & Mental Health, 2016. **20**(8): p. 793-803.
11. A-Tjak, J.G.L., et al., *A meta-analysis of the efficacy of acceptance and commitment therapy for clinically relevant mental and physical health problems.* Psychotherapy and Psychosomatics, 2015. **84**(1): p. 30-36.
12. Hann, K.E.J. and L.M. McCracken, *A systematic review of randomized controlled trials of Acceptance and Commitment Therapy for adults with chronic pain: Outcome domains, design quality, and efficacy.* Journal of Contextual Behavioral Science, 2014. **3**(4): p. 217-227.
13. Gloster, A.T., et al., *The empirical status of acceptance and commitment therapy: A review of meta-analyses.* Journal of Contextual Behavioral Science, 2020. **18**: p. 181-192.
14. Hayes, S.C., K.D. Strosahl, and K.G. Wilson, *Acceptance and commitment therapy: An experiential approach to behavior change*. 1999, New York, NY, US: Guilford Press.
15. McCracken, L.M. and R. Jones, *Treatment for Chronic Pain for Adults in the Seventh and Eighth Decades of Life: A Preliminary Study of Acceptance and Commitment Therapy (ACT).* Pain Medicine, 2012. **13**(7): p. 860-867.
16. Kishita, N., et al., *Which intervention work for dementia family carers?: An updated systematic review of randomised controlled trials of carer interventions.* International Psychogeriatrics, 2018. **30**(11): p. 1679-1696.
17. Thompson, E.M., et al., *Internet-based acceptance and commitment therapy: A transdiagnostic systematic review and meta-analysis for mental health outcomes.* Behavior Therapy, 2021. **52**(2): p. 492-507.
18. Brown, M., et al., *Effectiveness of web-delivered Acceptance and Commitment Therapy in relation to mental health and well-being: A systematic review and meta-analysis.* Journal of medical Internet research, 2016. **18**(8): p. e221.
19. Trindade, I.A., et al., *Efficacy of online-based Acceptance and Commitment Therapy for chronic pain: A systematic review and meta-analysis.* Journal of Pain, 2021. **22**(11): p. 1328-1342.

20. Scott, W., et al., *Feasibility randomized-controlled trial of online Acceptance and Commitment Therapy for patients with complex chronic pain in the United Kingdom.* European Journal of Pain 2018. **22**(8): p. 1473-1484.
21. Scott, W., et al., *Feasibility randomized-controlled trial of online acceptance and commitment therapy for painful peripheral neuropathy in people living with HIV: The OPEN study.* European Journal of Pain, 2021. **25**(7): p. 1493-1507.
22. Kishita, N., et al., *Internet-delivered guided self-help Acceptance and Commitment Therapy for family carers of people with dementia (iACT4CARERS): A feasibility study.* Aging and Mental Health, 2021. **26**(10): p. 1933-1941.
23. Kounali, D., et al., *How much change is enough? Evidence from a longitudinal study on depression in UK primary care.* Psychological Medicine, 2020. **52**(10): p. 1-8.
24. Blakemore, A., et al., *Dementia in UK South Asians: a scoping review of the literature.* BMJ Open, 2018. **8**(4): p. e020290.
25. NHS England, *The NHS Long Term Plan*. 2019, NHS England: London, UK.
26. NHS England, *Integrating care: Next steps to building strong and effective integrated care systems across England*. 2020, NHS England: London, UK.
27. Woidneck, M.R., et al., *Exploring cultural competence in acceptance and commitment therapy outcomes.* Professional Psychology: Research and Practice, 2012. **43**(3): p. 227-233.
28. Tol, W.A., et al., *Guided self-help to reduce psychological distress in South Sudanese female refugees in Uganda: a cluster randomised trial.* The Lancet Global Health, 2020. **8**(2): p. e254-e263.
29. Bricker, J.B., et al., *Efficacy of smartphone applications for smoking cessation: A randomized clinical trial.* JAMA Internal Medicine, 2020. **180**(11): p. 1472-1480.
30. Spitzer, R.L., et al., *A brief measure for assessing generalized anxiety disorder: the GAD-7.* Archives of Internal Medicine, 2006. **166**(10): p. 1092-1097.
31. Beecham, J. and M. Knapp, *Costing psychiatric interventions*, in *Measuring Mental Health Needs* G. Thornicroft, Editor. 2001, Gaskell: London. p. 200-224.
32. Cheng, S.-T. and F. Zhang, *A comprehensive meta-review of systematic reviews and meta-analyses on nonpharmacological interventions for informal dementia caregivers.* BMC Geriatrics, 2020. **20**(1): p. 137.
33. Kroenke, K., R.L. Spitzer, and J.B. Williams, *The PHQ-9: validity of a brief depression severity measure.* Journal of General Internal Medicine, 2001. **16**(9): p. 606-613.
34. Francis, A.W., D.L. Dawson, and N. Golijani-Moghaddam, *The development and validation of the Comprehensive assessment of Acceptance and Commitment Therapy processes (CompACT).* Journal of Contextual Behavioral Science, 2016. **5**(3): p. 134-145.
35. Losada, A., et al., *Development and validation of the Experiential Avoidance in Caregiving Questionnaire (EACQ).* Aging & Mental Health, 2014. **18**(7): p. 897-904.
36. Oei, T.P.S. and A.L. Green, *The Satisfaction With Therapy and Therapist Scale-Revised (STTS-R) for group psychotherapy: Psychometric properties and confirmatory factor analysis.* Professional Psychology: Research and Practice, 2008. **39**(4): p. 435-442.
37. Herdman, M., et al., *Development and preliminary testing of the new five-level version of EQ-5D (EQ-5D-5L).* Quality of Life Research, 2011. **20**(10): p. 1727-1736.
38. Grewal, I., et al., *Developing attributes for a generic quality of life measure for older people: Preferences or capabilities?* Social Science & Medicine, 2006. **62**(8): p. 1891-1901.
39. O'Neill, L., et al., *The development of the Acceptance and Commitment Therapy Fidelity Measure (ACT-FM): A delphi study and field test.* Journal of Contextual Behavioral Science, 2019. **14**: p. 111-118.
40. Pinnock, H., et al., *Standards for Reporting Implementation Studies (StaRI) Statement.* BMJ, 2017. **356**: p. i6795.
41. Malterud, K., V.D. Siersma, and A.D. Guassora, *Sample size in qualitative interview studies: Guided by information power.* Qualitative Health Research, 2015. **26**(13): p. 1753-1760.

42. French, K., N. Golijani-Moghaddam, and T. Schröder, *What is the evidence for the efficacy of self-help acceptance and commitment therapy? A systematic review and meta-analysis.* Journal of Contextual Behavioral Science, 2017. **6**(4): p. 360-374.
43. Cavanagh, K., et al., *Can mindfulness and acceptance be learnt by self-help?: A systematic review and meta-analysis of mindfulness and acceptance-based self-help interventions.* Clinical Psychology Review, 2014. **34**(2): p. 118-129.
44. Contreras, M.L., et al., *Internet-delivered guided self-help Acceptance and Commitment Therapy for family carers of people with dementia (iACT4CARERS): A qualitative study of carer views and acceptability.* 2021. **17**: p. 1.
45. Schafer, J.L., *Multiple imputation: a primer.* Statistical Methods in Medical Research, 1999. **8**(1): p. 3-15.
46. Jakobsen, J.C., et al., *When and how should multiple imputation be used for handling missing data in randomised clinical trials – a practical guide with flowcharts.* BMC Medical Research Methodology, 2017. **17**(1): p. 162.
47. Donald, B.R., *Multiple imputation for nonresponse in surveys*. 1987, New York: John Wiley & Sons, Inc.
48. National Institute for Health and Care Excellence, *Guide to the methods of technology appraisal 2013: Process and methods*. 2013, NICE: London, UK.
49. van Hout, B., et al., *Interim Scoring for the EQ-5D-5L: Mapping the EQ-5D-5L to EQ-5D-3L Value Sets.* Value in Health, 2012. **15**(5): p. 708-715.
50. Jones, C., R.T. Edwards, and B. Hounsome, *Health economics research into supporting carers of people with dementia: A systematic review of outcome measures.* Health and Quality of Life Outcomes, 2012. **10**(1): p. 142.
51. Curtis, L.A. and A. Burns, *Unit costs of health and social care 2019*. 2019, PSSRU: Kent, UK.
52. Faria, R., et al., *A guide to handling missing data in cost-effectiveness analysis conducted within randomised controlled trials.* PharmacoEconomics, 2014. **32**(12): p. 1157-1170.
53. Braun, V. and V. Clarke, *Using thematic analysis in psychology.* Qualitative Research in Psychology, 2006. **3**(2): p. 77-101.

11 Appendices

NA

12 Principal Investigator compliance statement

Principal Investigator agreement to confirm adherence to the protocol, the UK Policy Framework for Health and Social Care Research and GCP.

iACT4CARERS

The clinical and cost effectiveness of internet-delivered self-help Acceptance and Commitment Therapy for family carers of people with dementia (iACT4CARERS): A randomised controlled trial with ethnically diverse family carers

I, [Insert investigator name], confirm:

1. that [insert name of site] site is willing and able to comply with the requirements of the iACT4CARERS trial;
2. that I regularly treat the target population and believe the site has the potential for recruiting the required number of suitable subjects within the agreed recruitment period (figures included in the trial recruitment plan);
3. that I have sufficient time to properly conduct and complete the trial within the agreed trial period;
4. that I have supplied an up to date curriculum vitae, GCP certificate and/or other relevant documentation requested by NCTU, to demonstrate that I am qualified by education, training and experience to assume responsibility for the proper conduct of the trial at this study site;
5. that I have an adequate number of qualified staff and adequate facilities available for the foreseen duration of the trial to conduct the trial properly and safely;
6. that I will maintain a signature and delegation log of appropriately qualified persons to whom I have delegated trial related duties which includes confirmation that each member of staff is appropriately trained (including GCP) for the roles allocated to them, and will ensure this is made available to NCTU in a timely manner on request;
7. a research CV for each member of staff on the delegation log will be stored in the site file according to site policy;
8. that I take responsibility for ensuring all staff delegated trial related duties are adequately informed about the protocol and their trial related duties and functions, and that I will continue to take responsibility for regularly updating them as new information becomes available;
9. that I am aware of, and will comply with, the principles of GCP as given in the iACT4CARERS protocol compliance statement and the applicable regulatory requirements, and that a record of my GCP training is accessible and described on my current curriculum vitae;
10. that a record of GCP training is accessible for all staff delegated responsibilities in relation to the iACT4CARERS trial and who are named and approved on the site signature and

delegation of responsibilities log and that individual training evidence will be saved in the site file, for all staff, according to trust policies;

11. that I will permit routine and for-cause monitoring and auditing by NCTU, and inspection by the appropriate regulatory authorities, including the provision of direct access to source data and other participant notes and files as required; and
12. that I agree to archive and/or arrange for secure storage of iACT4CARERS trial materials and records for a minimum of 10 years after the close of the trial unless otherwise advised by the NCTU.

Agreement: Principal Investigator

Name	[insert name]
Signature	[insert wet signature]
Date	[insert date]

(Please return a copy of this signed agreement (only pages 47 and 48 to NCTU at *############@nctu.uea.ac.uk*.)
